# Safety and efficacy of the intranasal spray SARS-CoV-2 vaccine dNS1-RBD: a multicentre, randomised, double-blind, placebo-controlled, phase 3 trial

**DOI:** 10.1016/S2213-2600(23)00349-1

**Published:** 2023-12

**Authors:** Fengcai Zhu, Shoujie Huang, Xiaohui Liu, Qi Chen, Chunlan Zhuang, Hui Zhao, Jinle Han, Anjuli May Jaen, Thai Hung Do, Jonathan Grant Peter, Alexander Gonzalez Dorado, Louie S Tirador, Gelza Mae A Zabat, Ralph Elvi M Villalobos, Gemalyn Pineda Gueco, Lauren Livia Greta Botha, Shirley Patricia Iglesias Pertuz, Jiaxiang Tan, Kongxin Zhu, Jiali Quan, Hongyan Lin, Yue Huang, Jizong Jia, Xiafei Chu, Junyu Chen, Yixin Chen, Tianying Zhang, Yingying Su, Changgui Li, Xiangzhong Ye, Ting Wu, Jun Zhang, Ningshao Xia

**Affiliations:** a Jiangsu Provincial Center for Disease Control and Prevention, Public Health Research Institute of Jiangsu Province, Nanjing, China; b State Key Laboratory of Vaccines for Infectious Diseases, Xiang An Biomedicine Laboratory, Department of Laboratory Medicine, School of Public Health, Xiamen University, Xiamen, China; c National Institute of Diagnostics and Vaccine Development in Infectious Diseases, State Key Laboratory of Molecular Vaccinology and Molecular Diagnostics, Collaborative Innovation Center of Biologic Products, National Innovation Platform for Industry-Education Integration in Vaccine Research, Xiamen University, Xiamen, China; d National Institute for Food and Drug Control, Beijing, China; e Beijing Wantai Biological Pharmacy Enterprise, Beijing, China; f The Medical City Iloilo, Iloilo City, Philippines; g Pasteur Institute in Nha Trang, Nha Trang, Viet Nam; h University of Cape Town Lung Institute, Mowbray, South Africa; i Bluecare Salud S A S Centro Médico Integral, Bogota, Colombia; j St Paul's Hospital Iloilo, Iloilo City, Philippines; k Tropical Disease Foundation, Makati City, Philippines; l UP-Philippine General Hospital, University of the Philippines Manila, Manila, Philippines; m Angeles University Foundation Medical Center, Angeles City, Philippines; n REIMED Reiger Park, Johannesburg, South Africa; o Clínica de la Costa, Barranquilla, Colombia

## Abstract

**Background:**

The live-attenuated influenza virus vector-based intranasal SARS-CoV-2 vaccine (dNS1-RBD, Pneucolin; Beijing Wantai Biological Pharmacy Enterprise, Beijing, China) confers long-lasting and broad protection in animal models and is, to our knowledge, the first COVID-19 mucosal vaccine to enter into human trials, but its efficacy is still unknown. We aimed to assess the safety and efficacy (but not the immunogenicity) of dNS1-RBD against COVID-19.

**Methods:**

We did a multicentre, randomised, double-blind, placebo-controlled, adaptive design, phase 3 trial at 33 centres (private or public hospitals, clinical research centres, or Centre for Disease Control and Prevention) in four countries (Colombia, Philippines, South Africa, and Viet Nam). Men and non-pregnant women (aged ≥18 years) were eligible if they had never been infected with SARS-CoV-2, and if they did not have a SARS-CoV-2 vaccination history at screening or if they had received at least one dose of other SARS-CoV-2 vaccines 6 months or longer before enrolment. Eligible adults were randomly assigned (1:1) to receive two intranasal doses of dNS1-RBD or placebo administered 14 days apart (0·2 mL per dose; 0·1 mL per nasal cavity), with block randomisation via an interactive web-response system, stratified by centre, age group (18–59 years or ≥60 years), and SARS-CoV-2 vaccination history. All participants, investigators, and laboratory staff were masked to treatment allocation. The primary outcomes were safety of dNS1-RBD in the safety population (ie, those who had received at least one dose of dNS1-RBD or placebo) and efficacy against symptomatic SARS-CoV-2 infection confirmed by RT-PCR occurring 15 days or longer after the second dose in the per-protocol population (ie, those who received two doses, were followed up for 15 days or longer after the second dose, and had no major protocol deviations). The success criterion was predefined as vaccine efficacy of more than 30%. This trial is registered with the Chinese Clinical Trial Registry (ChiCTR2100051391) and is completed.

**Findings:**

Between Dec 16, 2021, and May 31, 2022, 41 620 participants were screened for eligibility and 31 038 participants were enrolled and randomly assigned (15 517 in the vaccine group and 15 521 in the placebo group). 30 990 participants who received at least one dose (15 496 vaccine and 15 494 placebo) were included in the safety analysis. The results showed a favourable safety profile, with the most common local adverse reaction being rhinorrhoea (578 [3·7%] of 15 500 vaccine recipients and 546 [3·5%] of 15 490 placebo recipients) and the most common systemic reaction being headache (829 [5·3%] vaccine recipients and 797 [5·1%] placebo recipients). We found no differences in the incidences of adverse reactions between participants in the vaccine and placebo groups. No vaccination-related serious adverse events or deaths were observed. Among 30 290 participants who received two doses, 25 742 were included in the per-protocol efficacy analysis (12 840 vaccine and 12 902 placebo). The incidence of confirmed symptomatic SARS-CoV-2 infection caused by omicron variants regardless of immunisation history was 1·6% in the vaccine group and 2·3% in the placebo group, resulting in an overall vaccine efficacy of 28·2% (95% CI 3·4–46·6), with a median follow-up duration of 161 days.

**Interpretation:**

Although this trial did not meet the predefined efficacy criteria for success, dNS1-RBD was well tolerated and protective against omicron variants, both as a primary immunisation and as a heterologous booster.

**Funding:**

Beijing Wantai Biological Pharmacy Enterprise, National Science and Technology Major Project, National Natural Science Foundation of China, Fujian Provincial Science and Technology Plan Project, Natural Science Foundation of Fujian Province, Xiamen Science and Technology Plan Special Project, Bill & Melinda Gates Foundation, the Ministry of Education of China, Xiamen University, and Fieldwork Funds of Xiamen University.

**Research in context**

**Evidence before this study**

We searched PubMed for clinical trials published from database inception to June 27, 2023, with the following terms: “(intranasal OR nasal OR mucosal OR aerosolized OR inhaled) AND (coronavirus OR COVID-19 OR SARS-CoV-2) AND (vaccine) AND (clinical trial)”. No language restrictions were applied. In addition to dNS1-RBD reported in this Article, the results associated with two other mucosal vaccines have been reported in peer-reviewed clinical trials, but without information on their efficacy. In an open-label phase 1 trial (NCT04816019), one of the two vaccines (ChAdOx1 nCoV-19, University of Oxford–AstraZeneca, Oxford, UK), which is an adenovirus-vectored SARS-CoV-2 vaccine administered intranasally, showed an acceptable tolerability profile but did not induce a consistent mucosal antibody response or a strong systemic response. The other vaccine (aerosolised Ad5-nCoV, Institute of Biotechnology–CanSino Biologics, Tianjin, China) is an aerosolised adenovirus type-5 vector-based SARS-CoV-2 vaccine, which was shown to be safe and elicited neutralising antibody responses in phase 1 and 2 clinical trials (NCT04552366 and NCT05043259). According to WHO's COVID-19 vaccine tracker and landscape for SARS-CoV-2 candidate vaccines (updated on March 30, 2023), 15 intranasal SARS-CoV-2 vaccines are being analysed in ongoing clinical trials, including eight viral vector vaccines, four protein subunit vaccines, two live-attenuated vaccines, and one inactivated vaccine; additionally, two inhaled or aerosolised viral vector SARS-CoV-2 vaccines are being analysed in clinical trials.

**Added value of the study**

To our knowledge, this study is the first to report efficacy data for an intranasal SARS-CoV-2 vaccine. We evaluated the safety and efficacy of dNS1-RBD with a two-dose regimen in this multicentre, randomised, double-blind, placebo-controlled, phase 3 trial. The results indicated that dNS1-RBD was well tolerated, with no vaccine-related serious adverse event reported. This trial did not meet the predefined efficacy criteria for success, but based on efficacy data, it can be preliminarily inferred that dNS1-RBD provides sustained protection without a rapid decline (≥15 days with a median follow-up duration of 161 days).

**Implications of all the available evidence**

There remains a need for clinical development of safe, broad-spectrum, and needle-free vaccines to protect against SARS-CoV-2 variants. As an intranasal SARS-CoV-2 vaccine developed using the ancestral SARS-CoV-2 strain, the available evidence suggests that the NS1-deleted and cold-adapted influenza virus vector-based SARS-CoV-2 vaccine is well tolerated and protects against COVID-19 caused by omicron variants. Our findings support the development of intranasal spray vaccines or other mucosal vaccines for respiratory infectious diseases and further investigation of the underlying protection mechanisms.

## Introduction

SARS-CoV-2 omicron sublineages have shown a strong ability to evade neutralising antibodies because of their continuous evolution.^1^ This unprecedented, rapidly shifting immune escape remains a global challenge in the control of the COVID-19 pandemic, which highlights the urgency for broad-spectrum SARS-CoV-2 vaccines.

Although evidence indicates that a booster dose of existing SARS-CoV-2 vaccines provides sustained protection against severe disease or hospitalisation caused by omicron variants, the effectiveness against symptomatic infections rapidly wanes within 3–6 months of vaccination for the primary series and booster dose.^2^ Additionally, the risk of post-acute sequelae might notably increase with SARS-CoV-2 reinfection.^3^ Nevertheless, the acceptance of regular booster doses by the general public, particularly vulnerable populations, is not optimal; a 2022 survey of SARS-CoV-2 vaccine acceptance across 23 countries showed that hesitancy increased in eight countries compared with vaccine acceptance in 2021, and almost 12% of vaccinated respondents were hesitant about booster doses.^4^ With the continuous emergence of multiple variants with substantially altered antigenicity and the scarcity of updated vaccination strategies, the prevention and control of COVID-19 has become more complicated.^5^, ^6^

The development of mucosal SARS-CoV-2 vaccines has been the subject of intense focus, owing to the advantage of inducing local immunity in the respiratory tract faster than effectors present in peripheral circulation.^7^, ^8^, ^9^ To date, many studies on intranasal SARS-CoV-2 vaccines are under way, yet the results of their pivotal trials have not been published so far (NCT05248373 and NCT05385991). The intranasal SARS-CoV-2 vaccine dNS1-RBD (Pneucolin, Beijing Wantai Biological Pharmacy Enterprise, Beijing, China) is manufactured with a cold-adapted, non-structural protein 1 (NS1)-deleted H1N1 influenza virus strain as the genetic backbone, into which a receptor-binding domain (RBD) from ancestral SARS-CoV-2 was inserted.^10^ In hamsters, dNS1-RBD has shown long-lasting, broad protection against various SARS-CoV-2 variants, and was associated with high RBD-specific T-cell responses in the respiratory tract, which were about 22 times stronger than those in peripheral blood; however, they were associated with weak serum antibody responses.^10^ Three early-phase clinical trials of dNS1-RBD have shown safety (at least one adverse reaction was reported in 19% of vaccine recipients and most reactions were mild; no vaccination-related serious adverse event was noted) and multiple immune responses in humans,^11^ with results similar to those observed in animal models.^10^

In December, 2022, dNS1-RBD obtained emergency use authorisation in China. We aimed to assess the safety and efficacy (but not the immunogenicity) of dNS1-RBD against COVID-19, representing a breakthrough in respiratory mucosal SARS-CoV-2 vaccine research.

## Methods

### Study design and participants

This multicentre, randomised, double-blind, placebo-controlled, case-driven, and adaptive design phase 3 trial was done at 33 sites (private or public hospitals, clinical research centres, or Center for Disease Control and Prevention) in Colombia, the Philippines, South Africa, and Viet Nam (appendix pp 2–3). Eligible participants were men and non-pregnant women aged 18 years or older without a history of SARS-CoV-2 infection, who had no SARS-CoV-2 vaccination history with a negative fingertip blood test result for SARS-CoV-2 antibodies (by commercial colloidal gold kits: WANTAI SARS-CoV-2 Ab Rapid Test [Beijing Wantai Biological Pharmacy Enterprise], Panbio COVID-19 IgG/IgM Rapid Test [Abbott Rapid Diagnostics, Jena, Germany] and Trueline COVID-19 IgG/IgM Rapid Test [Medicon, Hanoi, Viet Nam]) at screening, or who had received at least one dose of any other SARS-CoV-2 vaccines 6 months or more before signing informed consent for this study. Individuals with underlying, stable, chronic medical conditions were eligible for the trial. Full details of the inclusion and exclusion criteria are provided in the appendix (pp 3–5). In the Philippines, South Africa, and Viet Nam, information on sex was confirmed by their identification cards, whereas in Colombia, it was obtained from medical records. All participants provided written informed consent.

Participants in South Africa and Viet Nam were limited to those who had previously received one or more doses of SARS-CoV-2 vaccines due to the requirements of national regulatory authorities. On March, 2022, an addendum was added to the protocol (version 2.0, dated Aug 10, 2021) to include individuals in Viet Nam who had received the last vaccine dose 3–6 months before signing the informed consent, because a nationwide mass booster SARS-CoV-2 vaccination campaign was being carried out.

The trial protocol, the written informed consent form, and other materials related to the participants were approved by the ethics committees at all sites. The trial was done in accordance with the Declaration of Helsinki and the Good Clinical Practice guidelines. The study protocol is available in the appendix (pp 64–138).

### Randomisation and masking

All enrolled participants were randomly assigned (1:1) to receive two doses of either the dNS1-RBD vaccine or placebo. Block randomisation was done at each study centre by trained blinded investigators using an interactive web-response system, stratified according to centre, age group (18–59 years or ≥60 years), and the presence or absence of any previous SARS-CoV-2 vaccination. The unblinded statistician from the contract research organisation (Hangzhou Tigermed Consulting, Hangzhou, China) who designed the randomisation plan and generated the block randomisation codes was not involved further in the trial.

The vaccine and placebo were identical in appearance. To prevent cross-contamination between the vaccine and placebo groups during administration, which has been reported previously,^11^ all sites were required to set four separate rooms for vaccination, with two each for vaccine and placebo recipients respectively; additional measures taken to mitigate the risk of identifying the assignment are summarised in the appendix (pp 5–6). All participants, investigators, and laboratory staff were masked to treatment allocation. Project managers, research assistants, and professional inspection units regularly undertook inspections and no risk of unblinding was found.

### Procedures

The vaccine dNS1-RBD was a liquid preparation, containing 1 × 10^7^ cell culture infective dose 50% of dNS1-RBD per mL, whereas the placebo was composed of diluent without vaccine virus components. Both vaccines and placebo were supplied and stored at –15°C or lower. Participants received two doses of the vaccine or placebo (0·2 mL per dose; 0·1 mL per nasal cavity), 14 days apart, administered intranasally with a sprayer (NEST Biotechnology, Wuxi, China), which effectively atomises the liquid into a fine mist of droplets with a diameter of 10–70 μm.

All participants were observed for at least 30 min after vaccination for any acute reactions and were trained to record any local and systemic events using a diary card. Monitoring for adverse events included spontaneous reporting from participants and telephone contacts by investigators (once a week for 30 days after each dose for any adverse event, and at least once every 4 weeks after that for serious adverse events, medically attended adverse events, and adverse events of special interest). Definitions of these adverse events are provided in the appendix (pp 105–108).

After the first dose, all participants were monitored for suspected symptoms of COVID-19 by the investigators once per week for the entire efficacy observation period, via telephone or a visit within 30 days, and via telephone after 30 days. Suspected SARS-CoV-2 infection was defined as the presence of one or more of the following: (1) at least two symptoms, persisting for 2 days or longer, which included fever (oral temperature ≥38·0°C or axillary temperature ≥37·8°C), sore throat, generalised weakness or fatigue, rhinitis, myalgia, headache, lack of appetite, nausea or vomiting, diarrhoea, and mental status changes; changes in mental status were described as delirium (acute change in arousal and content), depression (chronic change in arousal), dementia (chronic change in arousal and content), and coma (dysfunction of arousal and content); (2) at least one respiratory sign or symptom, including cough (persisting for ≥2 days), loss of taste or smell (persisting for ≥2 days), and shortness of breath; or (3) clinical or imaging evidence of COVID-19. Once a suspected case was identified by a clinician or clinically qualified investigator, two nasopharyngeal swab samples were simultaneously collected (preferably within 72 h), with one swab sent to the central laboratory (Cerba Research, Paris, France) and the other to a local laboratory near each site or centre if in Colombia, South Africa, and Viet Nam, or to a local professional agency if in the Philippines (Detoxicare, Manila, Philippines). Clinical follow-up was guided by immediate RT-PCR results from the local laboratory. When the initial test result of the sample was negative but symptoms persisted, a second sampling was taken within 3–5 days. When the test result was positive, subsequent sampling occurred every 7–10 days until a negative result was obtained and symptoms resolved. We defined participants with a confirmed symptomatic SARS-CoV-2 infection as those with a suspected infection (according to the aforementioned criteria) who had at least one RT-PCR-positive nasopharyngeal swab tested by the central laboratory. Further details of the monitoring procedures, definition, and classification criteria of symptomatic SARS-CoV-2 infection according to WHO and China's National Health Commission are provided in the appendix (pp 6–9).

### Outcomes

The specific details regarding the safety and efficacy analyses were prespecified in the statistical analysis plan (version 2.0, dated Dec 6, 2022), which was finalised before data locking. The primary safety endpoints were solicited adverse events and reactions occurring within 7 days of either dose; adverse events and reactions occurring within 30 days of any dose; and serious adverse events, medically attended adverse events, and adverse events of special interest from the first dose (ie, day 0) to 12 months after the second dose.

Adverse events were graded according to the China National Medical Products Administration (NMPA) guidelines (appendix pp 130–133), with association between adverse events and vaccination determined by the investigators. Adverse reactions referred to any adverse event with at least a reasonable possibility of association with the vaccination in the trial (appendix pp 106, 108–109).

The primary efficacy endpoint was symptomatic SARS-CoV-2 infection confirmed by RT-PCR occurring 15 days or longer after the second dose. The secondary efficacy endpoints were virologically confirmed symptomatic SARS-CoV-2 infection of any severity in participants with or without a history of SARS-CoV-2 vaccination; in individuals of different age groups (18–59 years and ≥60 years); and in those with clear chronic disease; number of individuals with severe and critical COVID-19; and deaths from COVID-19. All secondary efficacy endpoints were assessed at 15 days or longer after the second vaccination.

Due to the low number of individuals with COVID-19 we observed and ongoing virological-related studies, some of the prespecified exploratory endpoints (appendix pp 85–86) could not be assessed or they will be reported elsewhere in the future.

### Statistical analysis

In accordance with the 2020 WHO guidelines,^12^ we calculated the sample size using the PASS software (version 11.0) on the basis of the following assumptions: a vaccine efficacy of 60% with a lower 95% CI of 30%, a 6-month infection rate of 0·85% in the placebo group, and an annual dropout rate of 20%. With a power of 90% and a one-sided α of 0·025, the trial was estimated to require 150 individuals for the primary efficacy endpoint and a sample size of 32 000–40 000 participants.

The originally planned interim analysis was scheduled to be done when the number of individuals for the primary efficacy endpoint reached 75 (appendix p 115). However, due to the unexpected rapid increase in the number of individuals with COVID-19, the number of adjudicated individuals for the primary efficacy endpoint in the per-protocol population (ie, those who received two doses, were followed up for 15 days or longer after the second dose, and had no major protocol deviations [appendix p 117]) had already exceeded 150 and triggered the final analysis before we could do the interim analysis.

The safety data were collected for 12 months after the final dose (ie, safety observation period; completed on June 30, 2023), whereas the efficacy analysis was case driven and the observation period for efficacy endpoints (ie, the primary efficacy analysis was triggered once the number of cases for the primary endpoint reached 150; appendix p 115) was concluded on July 31, 2022.

The safety analysis population comprised all participants who had received at least one dose of dNS1-RBD or placebo, and it was based on the actual administrations, correcting for vaccination errors. The primary efficacy analysis included the per-protocol population. Additional prespecified efficacy analyses were performed in the modified intention-to-treat population (ie, all randomly assigned patients who received two doses and were followed up for 15 days or longer after the second dose; listed in the protocol as mITT2).

The rate difference, defined as the difference in incidence of adverse events or reactions between the vaccine group and the placebo group, was used in the safety analysis to compare the safety profile of the vaccine and placebo. For each rate difference, we estimated the 95% CIs using the Wilson score method. We also did prespecified subgroup analyses of the safety endpoints by age group (18–59 years *vs* ≥60 years); underlying medical chronic conditions at baseline, defined as those that could increase the risk of SARS-CoV-2 infection (including hypertension, obesity, diabetes, thyroid disease, heart disease, and kidney disease); and underlying respiratory disease or nose-related diseases at baseline. A stratified Cox proportional hazards model was used to assess vaccine efficacy, with stratification by country, SARS-CoV-2 vaccination history, and age group, and with the efficacy estimated as 1 minus the hazard ratio (transformed to a percentage) and the 95% CIs similarly transformed. The success criterion required rejection of the null hypothesis (a vaccine efficacy of ≤30%) to show a vaccine efficacy that met the criteria for statistical significance (two-sided p<0·05). There was no allowance for multiplicity and all missing data were not imputed.

We also did analyses to assess vaccine efficacy in the short term (15–90 days; prespecified) and estimate the incidence of hospitalisation among participants who received at least one dose (post hoc). To further differentiate the severity of COVID-19 in non-hospitalised individuals or those with mild disease, we quantified disease severity according to the number of suspected symptoms in a post-hoc analysis, which was defined as omicron symptom index (appendix p 10). We did an exploratory analysis for efficacy against more typical symptomatic SARS-CoV-2 infections with three or more suspected symptoms persisting for 2 days or longer (ie, omicron symptom index ≥3) during the overall efficacy observation period and in the short term (15–90 days), in the per-protocol population, in the modified intention-to-treat population, and by vaccination history.

Independent statisticians from the contract research organisation (Hangzhou Tigermed Consulting) did all the analyses using SAS (version 9.4). An independent data monitoring committee was responsible for safeguarding trial participants, assessing safety during the trial period, and reviewing data after the final analysis. A blinded independent endpoint adjudication committee confirmed the endpoints and determined the severity of COVID-19 on the basis of clinical manifestation and related data. This study is registered with the Chinese Clinical Trial Registry (ChiCTR2100051391).

### Role of the funding source

The funders of the study had no role in study design, data collection, data analysis, data interpretation, or writing of the report.

## Results

**Figure 1:**
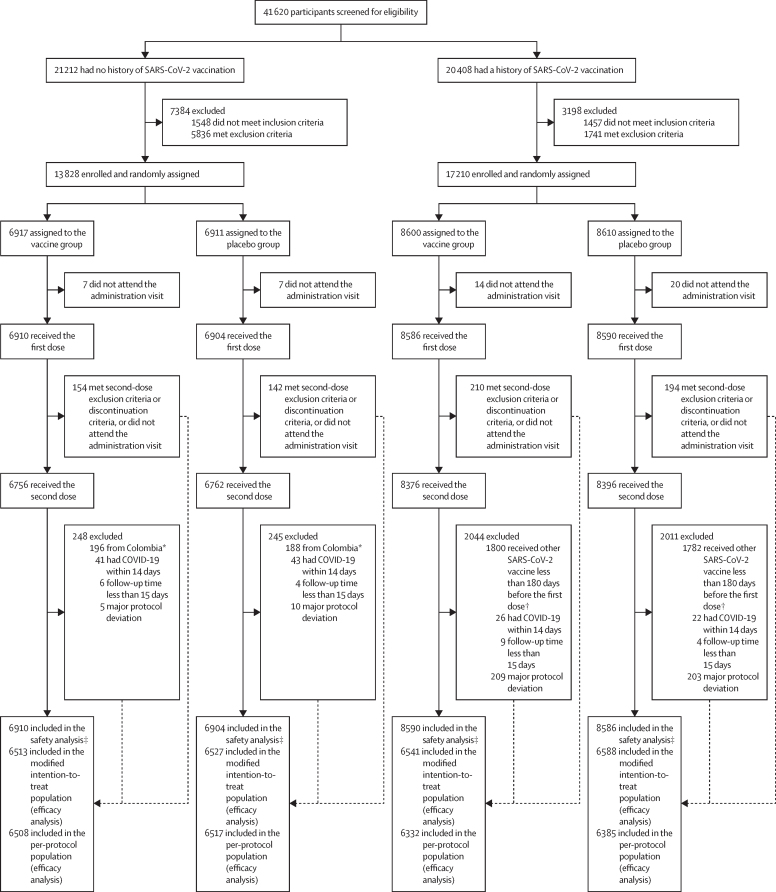
Trial profile *The 384 Colombian participants without previous SARS-CoV-2 vaccinations who received at least one dose were excluded from the primary efficacy analysis during the blind data review meeting for this trial because the sample size from each site was too small. †An addendum was made to the protocol to permit the enrolment of individuals in Viet Nam who received their last vaccine dose 3–6 months before signing the informed consent because of the ongoing national SARS-CoV-2 vaccine booster campaign, but these participants were not included in the primary efficacy analysis. ‡Eight participants had vaccination errors. Five participants (four with previous SARS-CoV-2 vaccinations and one without) were assigned to the placebo group but received at least one dose of dNS1-RBD, and were included in the vaccine group for the safety analysis; one participant without previous SARS-CoV-2 vaccinations who was assigned to the vaccine group but received two doses of placebo was included in the placebo group for the safety analysis; and two participants with previous SARS-CoV-2 vaccinations who were assigned to the vaccine group but received one dose of placebo and one dose of vaccine were included in the vaccine group for the safety analysis.

Between Dec 16, 2021, and May 31, 2022, 41 620 participants were screened for eligibility, of whom 31 038 were enrolled and randomly assigned (17 210 with a history of SARS-CoV-2 vaccination and 13 828 without; 15 517 in the vaccine group and 15 521 in the placebo group). 30 990 participants received at least one dose of the vaccine or placebo, including 17 176 participants with a history of SARS-CoV-2 vaccination and 13 814 participants without (figure 1; appendix pp 17–18). 30 290 participants received two doses of the vaccine or placebo (16 772 with a history of SARS-CoV-2 vaccination and 13 518 without).

Extreme difficulty in recruiting participants without previous SARS-CoV-2 vaccination in Colombia resulted in a smaller sample size than initially planned. 389 participants without a SARS-CoV-2 vaccination history were enrolled from eight centres (sample size range 4–159) who received at least one dose in this trial. A small sample size in each centre in Colombia might have resulted in additional uncertainties and confounding factors between treatment groups when the incidence of target disease is extremely high and fluctuates rapidly, and the independent randomisation done at each centre might lead to an uneven distribution of participant characteristics across the groups. In addition to the small sample size, there was also an increased possibility of false negative SARS-CoV-2 antibodies results at baseline due to an omicron (BA.1 and BA.2) outbreak in the country before enrolment. Because of the small sample size and possibility of false negatives, 389 Colombian participants (including eight participants with confirmed COVID-19) were excluded from all efficacy analyses (five of them were excluded firstly because they did not receive the second dose; appendix pp 12–13).

The median efficacy follow-up was 161 days (IQR 111–189), with an overall dropout rate of 2·2% (668/30 990) and no significant differences between the two groups (appendix p 19). Demographic and baseline characteristics of participants were well balanced between the vaccine and placebo groups. Among individuals receiving at least one dose, the median age was 38·0 years (IQR 27·0–52·0) in both the vaccine and placebo groups; across both groups, 4557 (14·7%) participants were aged 60 years or older, 15 299 (49·4%) were female, 15 690 (50·6%) were male, one (<0·1%) had undifferentiated sex, and 4441 (14·3%) had at least one underlying chronic disease (table 1). Of 17 176 participants with a SARS-CoV-2 vaccination history, 8766 (51·0%) tested positive for SARS-CoV-2 antibodies at baseline and 13 576 (79·0%) had received at least two doses of other SARS-CoV-2 vaccines (appendix p 22); the median interval between the last dose of a previous SARS-CoV-2 vaccine and the vaccination in this trial was 201 days (IQR 185–226). Demographic and baseline characteristics of participants in the per-protocol population are shown in the appendix (pp 20–21).

**Table 1:** Demographic and baseline characteristics in participants who received at least one dose*

		**Participants without a SARS-CoV-2 vaccination history**		**Participants with a SARS-CoV-2 vaccination history**		**Total**	
		Vaccine group (n=6910)	Placebo group (n=6904)	Vaccine group (n=8586)	Placebo group (n=8590)	Vaccine group (n=15 496)	Placebo group (n=15 494)
Age, years							
	Median (IQR)	33·0 (24·0–46·0)	34·0 (24·0–46·0)	42·0 (30·0–56·0)	42·0 (30·0–56·0)	38·0 (27·0–52·0)	38·0 (27·0–52·0)
Age group							
	18–59 years	6298 (91·1%)	6289 (91·1%)	6919 (80·6%)	6927 (80·6%)	13 217 (85·3%)	13 216 (85·3%)
	≥60 years	612 (8·9%)	615 (8·9%)	1667 (19·4%)	1663 (19·4%)	2279 (14·7%)	2278 (14·7%)
Sex							
	Male	3965 (57·4%)	3930 (56·9%)	3938 (45·9%)	3857 (44·9%)	7903 (51·0%)	7787 (50·3%)
	Female	2945 (42·6%)	2973 (43·1%)	4648 (54·1%)	4733 (55·1%)	7593 (49·0%)	7706 (49·7%)
	Undifferentiated†	0	1 (<0·1%)	0	0	0	1 (<0·1%)
Country							
	Colombia	198 (2·9%)	191 (2·8%)	1556 (18·1%)	1555 (18·1%)	1754 (11·3%)	1746 (11·3%)
	Philippines	6712 (97·1%)	6713 (97·2%)	3604 (42·0%)	3608 (42·0%)	10 316 (66·6%)	10 321 (66·6%)
	South Africa	0	0	1498 (17·4%)	1499 (17·5%)	1498 (9·7%)	1499 (9·7%)
	Viet Nam	0	0	1928 (22·5%)	1928 (22·4%)	1928 (12·4%)	1928 (12·4%)
Race and ethnicity							
	Asian	6712 (97·1%)	6713 (97·2%)	5532 (64·4%)	5536 (64·4%)	12 244 (79·0%)	12 249 (79·1%)
	White	0	0	23 (0·3%)	20 (0·2%)	23 (0·1%)	20 (0·1%)
	Black	0	0	1095 (12·8%)	1106 (12·9%)	1095 (7·1%)	1106 (7·1%)
	American Indian or Alaska Native	20 (0·3%)	23 (0·3%)	113 (1·3%)	126 (1·5%)	133 (0·9%)	149 (1·0%)
	Native Hawaiian or Other Pacific Islander	0	0	1 (<0·1%)	0	1 (<0·1%)	0
	Multiple	178 (2·6%)	168 (2·4%)	1822 (21·2%)	1802 (21·0%)	2000 (12·9%)	1970 (12·7%)
BMI, kg/m^2^		23·5 (4·6)	23·5 (4·5)	25·5 (5·7)	25·5 (5·7)	24·6 (5·3)	24·6 (5·3)
Underlying chronic condition‡							
	Yes	531 (7·7%)	529 (7·7%)	1720 (20·0%)	1661 (19·3%)	2251 (14·5%)	2190 (14·1%)
	No	6379 (92·3%)	6375 (92·3%)	6866 (80·0%)	6929 (80·7%)	13 245 (85·5%)	13 304 (85·9%)
SARS-CoV-2 IgG antibody status							
	Negative	6899 (99·8%)	6885 (99·7%)	4220 (49·2%)	4175 (48·6%)	11 119 (71·8%)	11 060 (71·4%)
	Positive	11 (0·2%)§	19 (0·3%)§	4358 (50·8%)	4408 (51·4%)	4369 (28·2%)	4427 (28·6%)
	Missing	0	0	8	7	8	7
Time since last priming dose of SARS-CoV-2 vaccine, days		..	..	201·0 (185·0–226·0)	201·0 (185·0–226·0)	..	..
SARS-CoV-2 vaccine types							
	Inactivated vaccine	..	..	2925 (34·1%)	2966 (34·5%)	..	..
	Adenovirus vector vaccine	..	..	2185 (25·4%)	2141 (24·9%)	..	..
	mRNA vaccine	..	..	2335 (27·2%)	2325 (27·1%)	..	..
	Recombinant subunit vaccine	..	..	170 (2·0%)	183 (2·1%)	..	..
	Mixed	..	..	970 (11·3%)	974 (11·3%)	..	..

Data are median (IQR) or n (%). Percentages might not total 100% because of rounding.

* Participants who received at least one dose of vaccine or placebo, based on randomised grouping information, not adjusted for actual administrations (ie, without correcting for vaccination errors).

† Undifferentiated sex means that sex cannot be determined on the basis of physiological characteristics.

‡ Underlying chronic conditions were those that were ongoing at baseline and could increase the risk of SARS-CoV-2 infection.

§ 30 participants had a positive test result for SARS-CoV-2 antibodies but were accidentally enrolled and categorised as participant without a SARS-CoV-2 vaccination history.

30 990 participants were included in the safety analysis set. Overall, adverse reactions including local and systemic were largely absent or mild (ie, about 96% of adverse reactions were grade 1 or 2; table 2), and the proportion of participants from either group who had any adverse events or reactions within 30 days of any dose was the same (adverse events: 2423 [15·6%] of 15 500 in the vaccine group *vs* 2416 [15·6%] of 15 490 in the placebo group; adverse reactions: 1924 [12·4%] of 15 500 in the vaccine group *vs* 1924 [12·4%] of 15 490 in the placebo group; table 2). 1895 (12·2%) vaccine recipients and 1887 (12·2%) placebo recipients had at least one solicited adverse reaction within 7 days of either dose. The incidence of medically attended adverse events, adverse events of special interest, and serious adverse events after any dose was similar in participants in the vaccine and placebo groups; no vaccine-related serious adverse events were reported (table 2; appendix pp 31–60).

**Table 2:** Adverse events and reactions that occurred after any dose in the safety population*

	**Participants without a SARS-CoV-2 vaccination history**				**Participants with a SARS-CoV-2 vaccination history**				**Total**			
	Vaccine group (n=6910)	Placebo group (n=6904)	Rate difference (95% CI)	p value	Vaccine group (n=8590)	Placebo group (n=8586)	Rate difference (95% CI)	p value	Vaccine group (n=15 500)	Placebo group (n=15 490)	Rate difference (95% CI)	p value
**Solicited adverse events within 7 days of either dose**												
Any	709 (10·3%)	725 (10·5%)	−0·24 (−1·40 to 0·92)	0·64	1262 (14·7%)	1242 (14·5%)	0·27 (−1·20 to 1·74)	0·67	1971 (12·7%)	1967 (12·7%)	0·03 (−1·69 to 1·76)	0·96
Grade ≥3	41 (0·6%)	38 (0·6%)	0·04 (−0·24 to 0·33)	0·74	45 (0·5%)	35 (0·4%)	0·14 (−0·15 to 0·43)	0·26	86 (0·6%)	73 (0·5%)	0·19 (−0·22 to 0·59)	0·30
**Solicited adverse reactions within 7 days of either dose**												
Any	675 (9·8%)	685 (9·9%)	−0·15 (−1·29 to 0·98)	0·76	1220 (14·2%)	1202 (14·0%)	0·20 (−0·99 to 1·39)	0·70	1895 (12·2%)	1887 (12·2%)	0·04 (−0·79 to 0·88)	0·91
Grade ≥3	41 (0·6%)	38 (0·6%)	0·04 (−0·24 to 0·33)	0·74	42 (0·5%)	31 (0·4%)	0·13 (−0·09 to 0·35)	0·20	83 (0·5%)	69 (0·4%)	0·09 (−0·09 to 0·27)	0·26
**Adverse events within 30 days after any dose**												
Any	898 (13·0%)	901 (13·1%)	−0·05 (−1·34 to 1·23)	0·92	1525 (17·8%)	1515 (17·6%)	0·11 (−1·20 to 1·41)	0·85	2423 (15·6%)	2416 (15·6%)	0·04 (−0·89 to 0·96)	0·93
Grade ≥3	53 (0·8%)	46 (0·7%)	0·1 (−0·22 to 0·42)	0·48	80 (0·9%)	66 (0·8%)	0·16 (−0·15 to 0·48)	0·25	133 (0·9%)	112 (0·7%)	0·14 (−0·09 to 0·36)	0·18
**Adverse reactions within 30 days after any dose**												
Any	685 (9·9%)	701 (10·2%)	−0·24 (−1·39 to 0·91)	0·64	1239 (14·4%)	1223 (14·2%)	0·18 (−1·02 to 1·38)	0·74	1924 (12·4%)	1924 (12·4%)	−0·01 (−0·85 to 0·83)	0·98
Grade ≥3	42 (0·6%)	39 (0·6%)	0·04 (−0·25 to 0·33)	0·74	43 (0·5%)	31 (0·4%)	0·14 (−0·08 to 0·36)	0·16	85 (0·5%)	70 (0·5%)	0·10 (−0·08 to 0·28)	0·23
**Medically attended adverse events during the safety observation period**												
Any	61 (0·9%)	45 (0·7%)	0·23 (−0·10 to 0·56)	0·12	359 (4·2%)	376 (4·4%)	−0·02 (−0·89 to 0·49)	0·52	420 (2·7%)	421 (2·7%)	−0·01 (−0·42 to 0·41)	0·96
Grade ≥3	27 (0·4%)	21 (0·3%)	0·09 (−0·14 to 0·31)	0·39	107 (1·2%)	104 (1·2%)	0·03 (−0·34 to 0·41)	0·84	134 (0·9%)	125 (0·8%)	0·06 (−0·17 to 0·29)	0·58
**Adverse events of special interest during the safety observation period**												
Any	43 (0·6%)	42 (0·6%)	0·01 (−0·28 to 0·31)	0·92	33 (0·4%)	36 (0·4%)	−0·04 (−0·25 to 0·18)	0·72	76 (0·5%)	78 (0·5%)	−0·01 (−0·19 to 0·17)	0·87
Grade ≥3	3 (<0·1%)	2 (<0·1%)	0·01 (−0·06 to 0·09)	>0·99	11 (0·1%)	9 (0·1%)	0·02 (−0·09 to 0·14)	0·66	14 (0·1%)	11 (0·1%)	0·02 (−0·05 to 0·09)	0·55
**Serious adverse events during the safety observation period**												
Any	40 (0·6%)	32 (0·5%)	0·12 (−0·16 to 0·39)	0·35	120 (1·4%)	122 (1·4%)	−0·02 (−0·43 to 0·38)	0·89	160 (1·0%)	154 (1·0%)	0·04 (−0·22 to 0·29)	0·74
Vaccination-related	0	0	NA	..	0	0	NA	..	0	0	NA	..

Data are n (%), unless otherwise stated. Any refers to all participants with any grade of adverse events or reactions. NA=not applicable.

* Participants who received at least one dose of vaccine or placebo, based on actual administrations.

**Figure 2:**
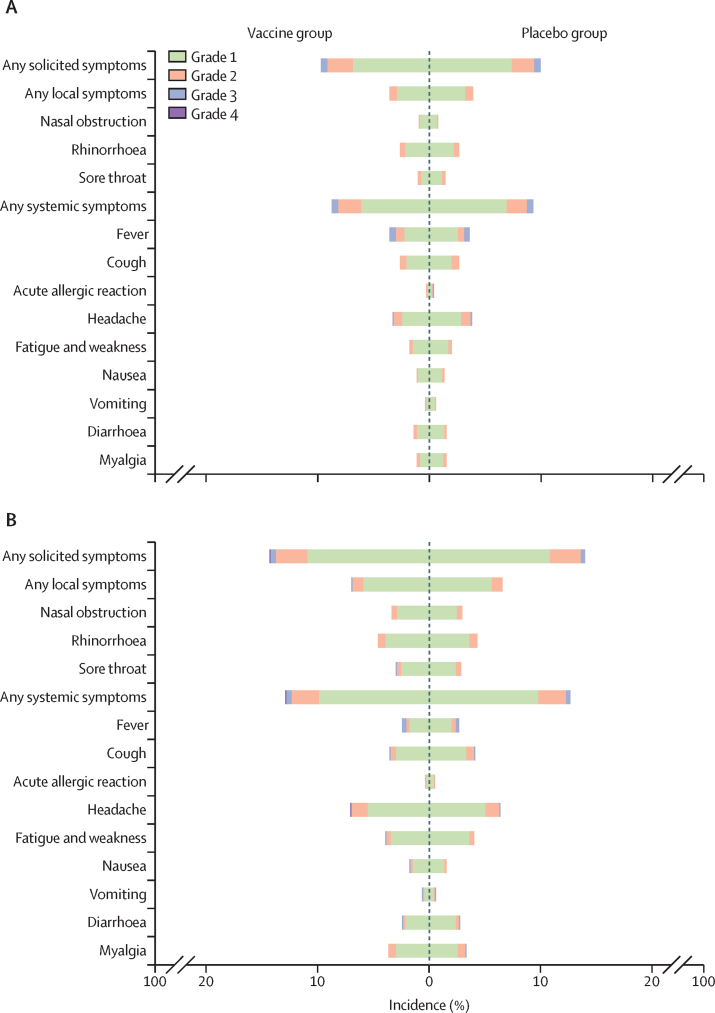
Solicited local and systemic adverse reactions that occurred within 7 days after any dose in the safety population* Incidence and severity of local and systemic adverse reactions in participants without a previous SARS-CoV-2 vaccination history (A) and in those with a SARS-CoV-2 vaccination history (B). All adverse reactions were graded according to the China National Medical Products Administration guidelines. *Participants who received at least one dose of vaccine or placebo.

Among all vaccine recipients, the most commonly reported local solicited adverse reactions were rhinorrhoea (578 [3·7%] of 15 500 participants in the vaccine group *vs* 546 [3·5%] of 15 490 in the placebo group), nasal obstruction (350 [2·3%] *vs* 297 [1·9%]), and sore throat (324 [2·1%] *vs* 337 [2·2%]); the most commonly reported systemic symptoms were headache (829 [5·3%] *vs* 797 [5·1%]), cough (486 [3·1%] *vs* 526 [3·4%]), fever (459 [3·0%] *vs* 474 [3·1%]), and fatigue and weakness (459 [3·0%] *vs* 473 [3·1%]; figure 2; appendix pp 23–24).

No difference was observed in the occurrence of adverse events and reactions between participants in the vaccine and placebo groups in all prespecified subgroups, including older recipients (aged ≥60 years) and participants with underlying chronic conditions at baseline (appendix pp 25–28). Although participants with underlying respiratory disease or nose-related diseases at baseline had higher incidence of adverse events and reactions compared with those in the overall safety population, we did not find differences between the vaccine and placebo groups in these two cohorts, apart from a larger number of serious adverse events (all vaccine-unrelated, as judged by the investigators and confirmed by the independent data monitoring committee) in participants in the vaccine group among participants with underlying respiratory diseases (eight [6·6%] *vs* one [0·9%]; appendix pp 29–30). Participants with underlying respiratory disease in the placebo group had higher numbers of systemic symptoms than those in the vaccine group. These differences are probably due to chance factors caused by the small number of events.

Among the 30 290 participants who received two doses, 25 742 were included in the per-protocol population for efficacy analysis (figure 1). During the efficacy observation period with a median duration of 161 days (IQR 111–189), 428 participants with confirmed symptomatic SARS-CoV-2 infection were identified, of whom 180 were included in the primary efficacy analysis (75 in the vaccine group and 105 in the placebo group). The reasons for exclusion are provided in the appendix (pp 11–12). All symptomatic SARS-CoV-2 infections were observed when different omicron variants were circulating globally, with the dominant sublineages including BA.1.1, BA.2, BA.4.1, BA.5.1, BA.5.2, and BA.5.6 in Colombia; BA.1, BA.2, and BA.5 in the Philippines; BA.2, BA.4, and BA.5 in South Africa, and BA.1.1, BA.2, BA.2.3, BA.2.3.2, BA.5.2, BA.5.2.1, and BE.1.1 in Viet Nam (appendix p 14).

**Figure 3:**
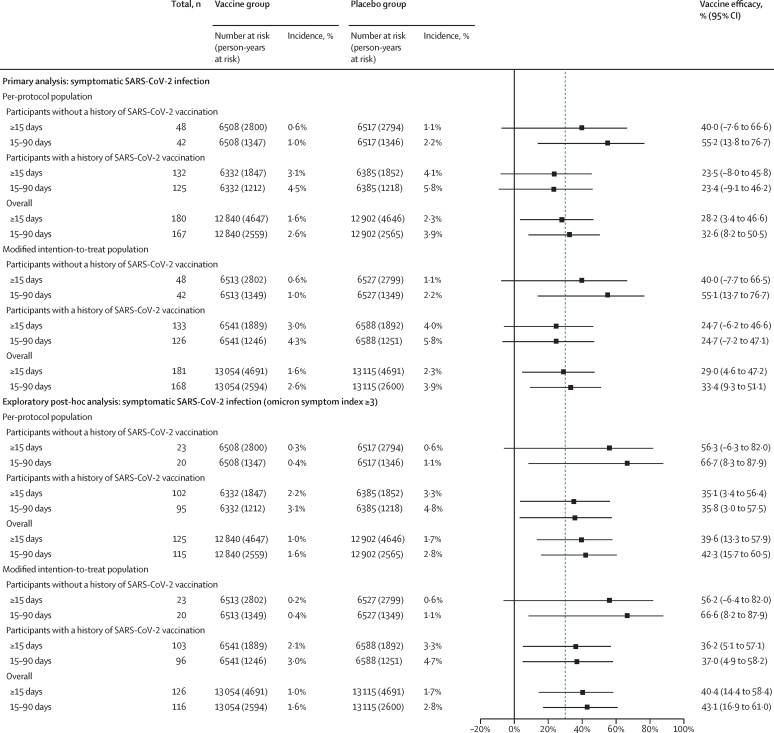
Vaccine efficacy 15 days or more after the second dose in the per-protocol population* and in the modified intention-to-treat population† The vertical line at 30% shows the prespecified vaccine efficacy target. *Participants who received two doses of vaccine or placebo, were followed up for 15 days or longer after the second dose, and had no major protocol deviations. †Participants who received two doses of vaccine or placebo and were followed up for 15 days or longer after the second dose.

Regarding the primary efficacy endpoint, regardless of baseline immunisation history, the two-dose vaccine efficacy in the per-protocol population against confirmed symptomatic SARS-CoV-2 infections during the efficacy observation period was 28·2% (95% CI 3·4 to 46·6; figure 3; appendix p 15), with a median follow-up duration of 161 days (IQR 111–189). Regarding the secondary efficacy endpoint, for the 13 025 participants without a history of SARS-CoV-2 vaccination included in the per-protocol population, the estimated two-dose vaccine efficacy against confirmed symptomatic SARS-CoV-2 infections was 40·0% (–7·6 to 66·6). For the 12 717 participants with a history of SARS-CoV-2 vaccination in the per-protocol population, the estimated two-dose vaccine efficacy was 23·5% (–8·0 to 45·8). Prespecified analyses showed that the short-term efficacy at 15–90 days was 32·6% (8·2 to 50·5) for participants in the per-protocol population regardless of vaccination history, 55·2% (13·8 to 76·7) for participants without previous SARS-CoV-2 vaccinations, and 23·4% (–9·1 to 46·2) for those with previous SARS-CoV-2 vaccinations. The analysis in the modified intention-to-treat population showed similar results (figure 3).

During the efficacy observation period, five participants in the placebo group (none in the vaccine group) were hospitalised (WHO score ≥4) with onset at any time after the first dose (appendix p 61). Three were categorised as having severe disease according to the NMPA criteria, one was categorised as general, and the other one as mild. No participant had critical COVID-19 or died in either group. Although severe SARS-CoV-2 infection was defined as one of the key secondary endpoints, the small number of participants with severe COVID-19 prevented the measurement of vaccine efficacy against severe disease. However, a post-hoc analysis suggested that the vaccine could provide 100% (95% CI –9·2 to 100·0) protection against hospitalisations caused by COVID-19, although this analysis was based on only the five participants who were hospitalised in this study (appendix p 16). The efficacy analysis results for age, country (an exploratory efficacy endpoint), and underlying chronic condition subgroups in the per-protocol and modified intention-to-treat populations showed similar efficacies to the two entire populations (appendix pp 62–63), but the lower 95% CIs for most efficacies were less than 30%, even less than 0%, because of the small number of cases within each subgroup.

In a post-hoc analysis of individuals with more typical symptomatic SARS-CoV-2 infections who reported three or more suspected symptoms (omicron symptom index ≥3), 125 participants were included (47 in the vaccine group and 78 in the placebo group). Among participants in the per-protocol population, the vaccine efficacy against typical symptomatic SARS-CoV-2 infections during the efficacy observation period (≥15 days) was 39·6% (95% CI 13·3 to 57·9), whereas the efficacy within 3 months (15–90 days) of the second dose was 42·3% (15·7 to 60·5). During the efficacy observation period, vaccine efficacy against typical infection was 56·3% (–6·3 to 82·0) in participants without a SARS-CoV-2 vaccination history and 35·1% (3·4 to 56·4) in those with a SARS-CoV-2 vaccination history (figure 3).

## Discussion

To our knowledge, this study is the first report on the efficacy of a mucosal SARS-CoV-2 vaccine in a large-scale phase 3 trial. The favourable safety profile observed in early phase clinical trials^11^ was also shown here, regardless of age, vaccination history, or underlying medical conditions. The efficacy data showed that the two-dose regimen of dNS1-RBD had an overall vaccine efficacy of 28·2% (95% CI 3·4–46·6) at 15 days or longer (with a median follow-up duration of 161 days [IQR 111–189]) against confirmed symptomatic SARS-CoV-2 infection regardless of baseline immunisation history, whereas the short-term efficacy at 15–90 days was 32·6% (8·2–50·5). The vaccine efficacy in participants without a history of SARS-CoV-2 vaccination at baseline was higher compared with the efficacy in participants who had a history of SARS-CoV-2 vaccination (40·0% *vs* 23·5%), which can be attributed to the fundamental disparity in pre-existing immune status between the two groups. Although this trial did not meet the predefined efficacy criteria for success (ie, a vaccine efficacy of >30%), based on efficacy data, it can be preliminarily inferred that dNS1-RBD provides sustained protection without a rapid decline (15–90 days *vs* ≥15 days with a median follow-up duration of 161 days).

Compared with parenteral injection, intranasal vaccination offers the advantages of being needle-free and non-invasive, thereby eliminating the pain and fear commonly associated with administration. Moreover, our study has shown the safety of the dNS1-RBD intranasal spray vaccine, including in older people (aged ≥60 years), in individuals with underlying medical conditions, and in those with respiratory or nose-related diseases. The widespread acceptance and ease of administration of intranasal vaccination hold promise for diminishing vaccine hesitancy and extending immunisation coverage during viral outbreaks, thereby alleviating disease burden, especially among vulnerable populations.

Although currently licensed SARS-CoV-2 vaccines might be effective against severe disease or death caused by some SARS-CoV-2 variants,^13^, ^14^ real-world studies have shown that the effectiveness against symptomatic infections caused by omicron variants wanes rapidly over 4–6 months, even after a booster dose.^2^, ^15^ In our trial, all SARS-CoV-2 infections occurred during the omicron-dominant period in all four countries studied (Colombia, March 22–July 31, 2022; the Philippines, Dec 16, 2021–July 31, 2022; South Africa, Feb 8–July 31, 2022; and Viet Nam, March 2–July 31, 2022). The data indicated that vaccinating adults with dNS1-RBD provides some protection against symptomatic SARS-CoV-2 infection caused by omicron (BA.2.3, BA.4.1, BA.2, BA.2.12.1, BA.5.2, BA.5.1, BA.4, XBB.1.2, etc), particularly against typical symptomatic infections with three or more COVID-19 symptoms as shown in a post-hoc analysis.

To date, efficacy data from randomised controlled clinical trials conducted during the omicron-dominant phase have rarely been reported, and those available mostly assessed vaccine efficacy in SARS-CoV-2 naive children (without infection or vaccination history). In the observer-blinded, placebo-controlled trial of mRNA-1273 (elasomeran, Spikevax; Moderna Biotech, Cambridge, MA, USA) published in 2022, the vaccine efficacy at around 70 days after the second dose against symptomatic SARS-CoV-2 infection caused by omicron (B.1.1.529) was 46·4% (95% CI 19·8 to 63·8) in children aged 2–5 years and 31·5% (–27·7 to 62·0) in children aged 6–23 months, which are lower than that against previously circulating variants of concern.^16^ Instead, the efficacy results of BNT162b2 (tozinameran, Comirnaty; Pfizer–BioNTech, New York, NY, USA), published in 2023, showed that with an average follow-up of less than 1·5 months after the third dose, the vaccine efficacy against symptomatic COVID-19 in children aged 6 months to 4 years was 73·2% (95% CI 43·8 to 87·6).^17^ However, according to data disclosed by the US Food and Drug Administration in June, 2022, the observed vaccine efficacy from 7 days after the second dose to administration of the third dose for BNT162b2 was 28·3% (95% CI 8·0 to 43·9) in children aged 6 months to 5 years.^18^ Given the same number of doses administered (two doses), we observed a similar efficacy of dNS1-RBD in our study during a longer follow-up period in adults to that observed with mRNA vaccines in children. Notably, a 2022 study highlighted the link between the maintenance of memory T cells in the respiratory tract and repeated antigen exposure.^19^ Considering results from the BNT162b2 studies,^17^, ^18^ the observed increase in efficacy with three doses versus two doses suggests that increasing the number of doses of dNS1-RBD could yield an improvement in efficacy, which warrants further investigation.

The most crucial challenge faced in the ongoing control of COVID-19 is the need for annual re-vaccination with updated formulations of the vaccines that often do not keep up with the rapid pace of viral variation.^20^ In real-world studies, the two bivalent mRNA vaccines approved for use in August, 2022 (Spikevax bivalent original/omicron BA.4–5, developed by Moderna Biotech, and Comirnaty original/omicron BA.4–5, developed by Pfizer), which were based on the ancestral SARS-CoV-2 and omicron BA.4 and BA.5 variants, have shown effectiveness of approximately 50% in terms of protection against hospitalisation or death.^21^, ^22^, ^23^ This lower than expected effectiveness has prompted a reconsideration of the future of SARS-CoV-2 vaccines by vaccine developers and the strategists for vaccine development and implementation, emphasising the need for the development of an ideal vaccine capable of inducing broad protective immunity. As a first-generation mucosal SARS-CoV-2 vaccine developed using the ancestral strain, dNS1-RBD has shown potential to be a broad-spectrum vaccine based on the different sublineages circulating during the trial. Additionally, preclinical studies have also shown that dNS1-RBD provides long-lasting (9 months), broad protection against at least ten SARS-CoV-2 variants in hamsters.^10^, ^24^ Consistently, several preclinical animal studies investigating other mucosal SARS-CoV-2 vaccines have reported that intranasal immunisation can confer durable protection in both the upper and lower respiratory tracts, targeting ancestral as well as emerging SARS-CoV-2 variants.^7^, ^25^, ^26^, ^27^ Currently, we are also actively involved in the development of the next generation of intranasal SARS-CoV-2 vaccines, focusing on updating the vaccine strains. However, the current ancestral strain-based intranasal vaccine has shown similar protective effects in hamsters challenged with the XBB and beta variants, compared with the vaccine candidate developed using the XBB strain (unpublished). Consequently, a straightforward replacement of the vaccine strain is not considered necessary at this stage, but new strategies might be explored in the future.

Nonetheless, the development of mucosal SARS-CoV-2 vaccines encounters challenges. First, the complexity of local mucosal immune responses poses a challenge in comprehending the mechanisms by which dNS1-RBD elicits broad protection. Second, the lack of validated sampling and detection methods for assessing cellular immunological markers in respiratory tracts has impeded the identification of immunological markers strongly associated with protection. In early human clinical trials,^11^ the weak neutralising antibody response in the mucosa and peripheral blood, induced by dNS1-RBD, is consistent with the findings from preclinical studies in mice.^10^ However, the T-cell immune response specific to the RBD induced in lung tissues of mice was about 22 times higher than that in peripheral blood,^10^ which is difficult to observe in humans because detecting T-cell immune responses requires lung tissue biopsies, which are hard to obtain from healthy individuals. Encouragingly, a cold-adapted, live-attenuated influenza intranasal vaccine (CAIV-T; Fluenz Tetra, AstraZeneca, London, UK), which was first licensed in 2003, conferred protection with weak or modest serum and mucosa antibody responses (the seroresponse rates of haemagglutination-inhibiting antibodies for influenza A/H1N1, A/H3N2, and B/Harbin were 23%, 33%, and 3%, and the response rates of IgA antibodies in nasal wash were 14%, 32%, and 18%, respectively).^28^ A human challenge trial^28^ and randomised controlled trial^29^ indicated that the response rates of haemagglutination-inhibiting antibodies in the serum of CAIV-T recipients were lower than those of the intramuscular trivalent inactivated influenza vaccine (TIV; seroresponse rates were 91% for A/H1N1, 76% for A/H3N2, and 76% for B/Harbin); however, the estimated efficacy of CAIV-T was higher than that of TIV (80% *vs* 60% for A/H1N1, 78% *vs* 67% for A/H3N2, and 100% *vs* 100% for B/Harbin).^28^

Evidence suggests that the first infection and replication of SARS-CoV-2 occurs in the nasal epithelium,^30^ and thus early control and prevention of transmission are heavily dependent on robust mucosal immune responses in the upper respiratory tract.^31^, ^32^ The local immune protective factors in the nasal epithelium offer a distinct advantage in terms of their proximity to potential viral entry points, compared with systemic immunity.^33^ Initiating antiviral effects within the nasal epithelium could effectively hinder progression of the disease at an early stage, which is crucial in preventing infections caused by omicron variants characterised by a short incubation period of 2–4 days. Although, thus far, high concentrations of specific secretory IgA antibodies in the mucosa and detectable neutralising antibodies in serum have not been observed after administration of dNS1-RBD,^10^, ^11^ current data indicate that the protection conferred by dNS1-RBD might be attributed to multiple host protective mechanisms covering the entire respiratory tract, such as the cellular immune response, mucosal antibody response, innate immunity including tissue-resident memory T cells, and the recently appreciated phenomenon of trained immunity (ie, innate immune memory effects achieved by reprogramming chromatin accessibility^34^). In contrast to traditional vaccines that aim to generate neutralising antibodies, which are prone to be escaped by SARS-CoV-2 spike protein variants,^35^ the protective immunity beyond antibody-mediated conferred by this intranasal vaccine might have a unique advantage against emerging variants.

This trial has several limitations. First, it was not powered to assess efficacy against severe disease due to the small number of participants with severe COVID-19. We did not anticipate that the omicron sublineages would have stronger transmissibility and immune escape ability, but substantially attenuated pathogenicity.^36^ Second, the colloidal gold reagents used for baseline SARS-CoV-2 antibodies screening were developed using ancestral SARS-CoV-2 and have decreased sensitivity in detecting antibodies induced by omicron infection, which might have resulted in individuals with an asymptomatic infection history being mistakenly enrolled into the population without a history of SARS-CoV-2 vaccination. Third, no immunological indicator was measured in this study, because no appropriate human immunological indicator directly related to the efficacy of dNS1-RBD was found in preclinical or early clinical trials. Finally, the status of influenza virus infection or anti-H1N1 pre-existing immunity was not detected at baseline. Although there was no evidence that pre-existing serum anti-H1N1 IgG antibodies had a negative effect on T-cell responses induced by dNS1-RBD in early clinical trials,^11^ the effect of pre-existing immunity against influenza virus on vaccine efficacy needs further verification in a large population.

In conclusion, although this clinical trial did not meet the predefined efficacy criteria for success, we have shown the potential of this intranasal spray influenza virus vector-based SARS-CoV-2 vaccine as a safe and broad-spectrum next-generation vaccine. In-depth studies, such as real-world studies and mechanistic research, are ongoing.

## Data sharing

The study protocol is available for review. The data in this Article will be available after publication and finalisation of the complete clinical study report for at least 6 months. Researchers who provide a scientifically sound proposal will be allowed to access the de-identified individual participant data. Proposals should be sent to the corresponding author TW (wuting@xmu.edu.cn). Proposals will be reviewed and approved by the sponsor, investigator, and collaborators. To gain access, data requestors will need to sign a data access agreement.

## Declaration of interests

JT, JJ, and XC were employees of Beijing Wantai Biological Pharmacy Enterprise during the conduct of the study. JH and XY are employees of and have stock options in Beijing Wantai Biological Pharmacy Enterprise. All other authors declare no competing interests.
