# Supplementary material for: Safety and efficacy of the intranasal spray SARS-CoV-2 vaccine dNS1-RBD: a multicentre, randomised, double-blind, placebo-controlled, phase 3 trial
Source: Lancet Respir Med. 2023 Dec;11(12):1075–88. doi: 10.1016/S2213-2600(23)00349-1 (PMC10682370; doi:10.1016/S2213-2600(23)00349-1)
Supplement: Supplementary appendix [file mmc1.pdf]

THE LANCET
Respiratory Medicine

Supplementary appendix

This appendix formed part of the original submission and has been peer reviewed. We post it as supplied by the authors.

Supplement to: Zhu F, Huang S, Liu X, et al. Safety and efficacy of the intranasal spray SARS-CoV-2 vaccine dNS1-RBD: a multicentre, randomised, double-blind, placebo-controlled, phase 3 trial. *Lancet Respir Med* 2023; published online Nov 15. https://doi.org/10.1016/S2213-2600(23)00349-1.

Supplementary Appendix

Contents

1. List of members of COVID-19-PRO-003 Study Team 1

2. Supplementary Method 2

2.1 Study sites 2

2.2 Inclusion and exclusion criteria for participants 3

2.3 Cross-contamination during administration 5

2.4 Monitoring Procedures of COVID-19 Cases 6

2.5 Follow-up of Confirmed COVID-19 Cases 7

2.6 Classification criteria of COVID-19 cases 8

2.7 Omicron symptom index (OSI) 10

3. Supplementary Figures 11

Figure S1. The prevalence of COVID-19 in the screening period. 11

Figure S2. 180 primary end point COVID-19 cases 12

Figure S3. The SARS-CoV-2 variant sublineages circulating in the study regions during the trial. 14

Figure S4. The cumulative Incidence of symptomatic SARS-CoV-2 infections in per-protocol population. 15

Figure S5. Incidence of hospitalized SARS-CoV-2 infection (WHO score ≥ 4) in the participants who received at least one dose. 16

4. Supplementary Tables 17

Table S1. The distribution of the prior COVID-19 vaccination history in participants who received at least one dose 17

Table S2. Number of participants who received at least one dose at each center in the four countries. 18

Table S3. The duration of follow-up and the dropout rate during the efficacy observation period in participants who received at least one dose. 19

Table S4. Demographics and baseline characteristics in per-protocol population 20

Table S5. The number of doses of prior COVID-19 vaccination. 22

Table S6. Local and systemic solicited adverse reactions that occurred within 7 days after any dose in safety analysis set. 23

Table S7. Adverse events or reactions that occurred after any dose in safety analysis set, stratified according to age group. 25

Table S8. Adverse events or reactions that occurred after any dose in safety analysis set, stratified according to the presence of underlying chronic condition* at baseline. 27

Table S9. Adverse events or reactions that occurred after any dose in participants with underlying respiratory disease or nose-related diseases at baseline in safety analysis set. 29

Table S10. Medically attended adverse events in safety analysis set throughout the observation period. 31

Table S11. Adverse events of special interest in safety analysis set throughout the observation period. 48

Table S12. List* of Adverse events of special interest in safety analysis set throughout the observation period. 49

Table S13. Serious adverse events in safety analysis set throughout the observation period. 50

Table S14. Demographics and clinical profiles of hospitalized SARS-CoV-2 infection (WHO score ≥ 4) in the participants who received at least one dose. 61

Table S15. Vaccine efficacy ≥ 15 days after the second dose against symptomatic COVID-19, subgroup analysis. 62

5. Protocol 64

1. List of members of COVID-19-PRO-003 Study Team

Name	Institute	Location
Investigators		
Abundio Balgos	The Health Centrum Hospital	Roxas City 5800, Philippines
Agatha Cathrine Wilhase	REIMED Welkom	Welkom 9459, South Africa
Aileen Zagala-Jazon	Premier Medical Center	Cabanatuan City 3100, Philippines
Carlos Enrique Ríos Sossa	Mediservis del Tolima IPS S.A.S	Ibagué 730017, Colombia
Erwin Pardo Iza	CAIMED Armenia S.A.S	Armenia 630008, Colombia
Evan Vista	St. Luke's Medical Center, Global City, St. Luke’s Medical Center and College of Medicine	Taguig City 1630, Philippines
Grace Pableo-Aquitania	Davao Medical School Foundation Hospital	Davao City 8000, Philippines
Gregorio Sánchez	Fundación Cardiomet Cequín, Universidad del Quindio, Hospital San Juan de Dios	Armenia 630008, Colombia
Ivan Darío Vélez B	Programa de estudios y control de enfermedades tropicales, Universidad de Antioquia	Medellín 050037, Colombia
Jeveren Reddy	Umzimkhulu Research Centre	Umzimkhulu 3297, South Africa
Joel Santiaguel	Quirino Memorial Medical Center	Quezon City 1109, Philippines
Johannes Breedt	EMMED Research	Waterkloof Ridge 0181, South Africa
Johannes Jurgens Lombaard	JOSHA Research	Bloemfontein 9301, South Africa
Jorge A. Egurrola Pedraza	Clinisalud del Sur S.A.S, Universidad del Magdalena	Envigado 055428, Colombia
Juan J Jaller-Raad	CIMEDICAL S.A.S	Barranquilla 080003, Colombia
Kathryn Theresen Mngadi	Aurum Tembisa	Tembisa 1632, South Africa
Loreta Zoleta	Silang Specialist Medical Center	Silang 4118, Philippines
Naomie Kupangi Kapanga	Progress Clinical Research Unit	Johannesburg 2040, South Africa
Ronald Allan Payumo	Mary Johnston Hospital	Manila 1012, Philippines

Herschel Don Go	UP-Philippine General Hospital, University of the Philippines Manila	Manila 1000, Philippines
Sponsor		
Xiaowen Hu	Xiamen University	Xiamen 361102, China
Xingcheng Huang	Xiamen University	Xiamen 361102, China
Jingjing Zhai	Beijing Wantai Biological Pharmacy Enterprise	Beijing 102206, China
Longjing Zhu	Beijing Wantai Biological Pharmacy Enterprise	Beijing 102206, China
Yuting Qi	Beijing Wantai Biological Pharmacy Enterprise	Beijing 102206, China

2. Supplementary Method

2.1 Study sites

No.	Principal Investigator	Location	Country
1	Joel Santiaguel	Quirino Memorial Medical Center	The Philippines
2	Anjuli May Jaen	The Medical City - Iloilo	The Philippines
3	Loreta Zoleta-De Jesus	Silang Specialist Medical Center	The Philippines
4	Louie S. Tirador	St. Paul's Hospital Iloilo	The Philippines
5	Ronald Allan Payumo	Mary Johnston Hospital	The Philippines
6	Abundio A. Balgos	The Health Centrum Hospital	The Philippines
7	Evan Glenn S. Vista	St. Lukes' Medical Center, Global City	The Philippines
8	Aileen Zagala-Jazon	Premier Medical Center	The Philippines
9	Gelza Mae A. Zabat	Tropical Disease Foundation, Inc	The Philippines
10	Ralph Elvi M. Villalobos	University of the Philippines - Philippine General Hospital	The Philippines
11	Gemalyn Pineda Gueco	Angeles University Foundation Medical Center	The Philippines
12	Grace Pableo-Aquitania	Davao Medical School Foundation Inc	The Philippines
13	Lauren Livia Greta Botha	REIMED Reiger Park	South Africa
14	Agatha Cathrine Wilhase	REIMED Welkom	South Africa
15	Johannes Breedt	EMMED Research	South Africa
16	Jeevren Reddy	Umzimkhulu Research Centre	South Africa
17	Kathryn Therese Mngadi	Aurum Tembisa	South Africa
18	Johannes Jurgens Lombaard	JOSHA Research	South Africa
19	Naomie Kupangi Kapanga	Progress Clinical Research Unit	South Africa
20	Jonathan Grant Peter	University of Cape Town Lung Institute (Pty) Ltd	South Africa
21	Gregorio Sánchez	Fundación Cardiomet Cequín	Colombia
22	Carlos Enrique Ríos Sossa	Mediservis del Tolima IPS SAS	Colombia
23	Erwin Pardo Iza	CAIMED Armenia S.A.S.	Colombia
24	Alexander Gonzalez Dorado	Bluecare Salud S.A.S. Centro Médico Integral	Colombia

25	Juan J Jaller-Raad	Centro de Investigación Médico Asistencial S.A.S. - CIMEDICAL S.A.S.	Colombia
26	Shirley Iglesias Pertuz	Clínica de la Costa Ltda.	Colombia
27	Ivan Darío Vélez B	Programa de estudios y control de enfermedades tropicales PECET. Universidad de Antioquia	Colombia
28	Jorge A. Egurrola Pedraza	Clinisalud del Sur S.A.S	Colombia
29	Do Thai Hung	Pasteur Institute Nha Trang	Vietnam
30		Ninh Hoa Medical Center	Vietnam
31		Dien An Commune Health Center	Vietnam
32		Dien Ban Town Medical Center	Vietnam
33		Quang Nam Center for Disease Control and Prevention	Vietnam

2.2 Inclusion and exclusion criteria for participants

Inclusion Criteria

Subjects participating in this study need to meet all the following criteria:

1) Aged ≥18 years old at the time of enrollment;

2) Be able to comply with the requirements of clinical study protocol and complete all trial procedures, and sign informed consent form;

3) Subjects who have not received any Covid-19 vaccine (marketed or investigational), those who have received at least one dose of other Covid-19 vaccines (marketed or investigational) with an interval of ≥ 6 months between the last dose and the date when the subjects sign the informed consent for this study;

4) Those who are negative for HIV screening (depending on the relevant policy of the country where the trial is conduct, if qualification for HIV testing is required in the country, this information will be obtained mainly by inquiry while protecting the subject's privacy);

5) Fertile males and females of childbearing potential who are willing to take appropriate contraceptive measures from signing ICF to 3 months after the last dose, including abstinence or effective contraceptive measures (e.g., intrauterine or implantable contraceptive devices, oral

contraception, combination of contraceptive diaphragm or condom with contraceptive gel); women of childbearing potential should be negative for pregnancy test on the day of vaccination (if applicable).

6) Healthy people or people with a mild underlying disease that has remained stable without exacerbation (not requiring hospitalization or without major modification of the treatment regimen) within at least 3 months prior to inclusion in the study.

Exclusion Criteria

Subjects who meet any one of the following criteria will be excluded from this study:

1) Prior history of Covid-19, or SARS-CoV-2 RT-PCR-positive at screening;

2) Positive test result of SARS-CoV-2-specific antibody at screening [Only applicable to subjects without vaccination history of Covid-19 vaccine (marketed or investigational)];

3) Pregnant or lactating women;

4) Fever on the day of vaccination or within 3 days prior to vaccination (oral temperature ≥37.5°C/ear temperature ≥ 37.5°C / axillary temperature ≥37.3°C);

5) Those who had any acute disease in the past 5 days that requires systemic antibiotic or antiviral treatment (including but not limited to the use of anti-influenza virus drugs such as Tamiflu, Relenza, Symmetrel or Flumadine);

6) Those who had low immune function caused by immunodeficiency diseases, diseases of important organs, cancer, and immune diseases (e.g., Guillain Barre syndrome, systemic lupus erythematosus, rheumatoid arthritis, alienia or splenectomy caused by any condition, and other immune disease that may affect immune response at the investigator's discretion);

7) Long-term use (defined as ≥14 days) of immunosuppressants or other immunomodulators (for glucocorticoids, e.g., ≥10 mg/day prednisone or equivalent dose; inhaled and topical steroids are

allowed) within 6 months prior to the first vaccination;

8) History of hemorrhagic diseases (e.g., factor deficiency, thrombocytopenia or other coagulation disorders), or hemorrhagic tendency, or continuous requirement of anticoagulants;

9) Having been injected with immunoglobulins and/or blood products within 3 months before receiving the investigational vaccine;

10) Received subunit or inactivated vaccine within 14 days before vaccination, or received live attenuated vaccine within 28 days before vaccination;

11) Participation in a clinical trial of another product within 1 month prior to vaccination, or planning to participate in a clinical trial of another product during the study;

12) Having a history of severe allergic reactions or severe adverse reactions from previous immunizations, or allergy to any component of the investigational vaccine;

13) Patients deemed by the investigator as unsuitable for using nasal spray (those with severe rhinitis or nasal deformities, etc.);

14) Planning to relocate permanently from the current area prior to the completion of the study or to leave the current area for a long period (preventing compliance with the prescribed visit schedule) during the study visits;

15) Other conditions that the investigators consider unsuitable for this clinical study.

2.3 Cross-contamination during administration

The cross-contamination during administration caused by the aerosol in the phase 1 and 2 clinical trials for this vaccine candidate has been reported previously. To avoid cross-contamination in this trial, all study sites were required to set four separate rooms for vaccination (rooms A, D, K and Y), with two each for vaccine and placebo recipients respectively. Each participant was assigned a computer-generated randomization code, a unique investigational product code, and a room code (A,

D, K and Y) by order of enrolment, and was assigned to the same treatment group for their second dose. The randomization code was always hidden during the trial, with only the unblinded statistician able to check it on the system. The room code was only accessible to the designated investigators for a limited time, and the documents involving the room code were sealed in a separate envelope after use.

2.4 Monitoring Procedures of COVID-19 Cases

All subjects will be followed up for suspected SARS-CoV-2 infection from after the first dose. Confirmed COVID-19 cases will be captured by both active and passive monitoring. All subjects will use diary cards to record/report suspected COVID-19 symptoms, and will receive a contact card containing the investigator's contact information and designated medical institutions.

Each study site will establish a 24/7 communication service to maintain contacts with the subjects. During the post-vaccination self-safety observation training process, subjects will be told to call the service in the event of any disease.

Through a diary card, the subject will record/report any symptoms that may be related to COVID-19 from after the first vaccination (V2) to V3 + 30 days (with a window period of +14 days), and the study staff will weekly make inquiries via telephone or other communication methods. After V3 + 30 days (with a window period of +14 days) until the unblinding in the site, the study staff will make inquiries for any related signs/symptoms every week. The window period for the above remote visits is ±3 days.

SARS-CoV-2 nucleic acid test (RT-PCR) will be triggered by any of the following during the study:

1. Any two or more of the following symptoms (persisting ≥ 2 days): fever (oral temperature≥ 38.0°C or ear temperature ≥ 38 °C or axillary temperature≥ 37.8°C); sore throat; weakness generalized/fatigue (if two symptoms appear simultaneously, only one symptom is counted); rhinitis; myalgia; headache; lack of appetite/nausea/vomiting (if three symptoms appear simultaneously, only one symptom is counted); diarrhea; mental status changes.

2. Any one or more of the following symptoms: cough (persisting≥2 days); loss of taste or smell (persisting≥2 days); dyspnea.
3. Clinical or imaging evidences of COVID-19 (if any).

For subjects who have the above suspected COVID-19 symptoms, nasopharyngeal swabs should in principle be collected in the designated hospital/health agency within 72 hours, but if it’s undoable, nasopharyngeal swabs should be collected timely upon the notice of such suspected cases; (If nasopharyngeal swabs cannot be collected, oropharyngeal swabs should be collected, or additional oropharyngeal swabs can be collected in the same collection tube based on local policy. If nasopharyngeal swabs are not collected, please note the reason), or by the investigator at his/her home or using other effective sampling methods at the investigator’s discretion. Two samples (one nasopharyngeal swab each in one nasal cavity) will be collected for SARS-CoV-2 RT-PCR test, one will be immediately tested by the local laboratory of the study institution for timely clinical diagnosis and treatment, the other will be sent to the central lab to confirm whether it is the end point case.

For tests performed at the local study institution’s laboratory, if the test result is negative but the symptoms persist, a second sampling test should be performed within 3 to 5 days. If the second sampling test result remains negative, the test will not be repeated. If the local test result is positive, it will be followed as COVID-19 confirmed cases (see section 1.3). The RT-PCR test results of the central laboratory will be regarded as the final results in this study.

2.5 Follow-up of Confirmed COVID-19 Cases

For subjects confirmed as COVID-19 cases, the date of onset of symptoms related to COVID-19 will be taken as the date of onset.

Subjects confirmed as COVID-19 cases will be managed and treated according to the local policy. After being confirmed with COVID-19, the subject will be followed up by the investigator every 7-10 days until RT-PCR is negative and the symptoms have resolved (If symptoms persist after two consecutive negative test results, investigators will decide whether to continue sampling for RT-PCR testing). The treatment status and medical history of the subjects will be collected. An independent

endpoint adjudication committee (EAC) whose members are unaware of the treatment allocation judge end points and determine the severity of COVID-19 on the basis of all data.

2.6 Classification criteria of COVID-19 cases

WHO Criteria for Grading of COVID-19 Cases [1]

Status:	Definition	Score
Uninfected	Not infected, nucleic acid negative	0
Mild	Asymptomatic, nucleic acid positive;	1
	Mild symptoms, not requiring treatment	2
	Mild symptoms, requiring treatment	3
General cases requiring hospitalization	Hospitalization; no oxygen therapy required*	4
	Hospitalization; oxygen therapy through a mask or nasal cavity	5
Severe cases requiring hospitalization	Treatment by non-invasive ventilation or high flow oxygen inhalation	6
	Intubation and mechanical ventilation, $pO_2/FiO_2 \geq 150$, or $SpO_2/FiO_2 \geq 200$	7
	Mechanical ventilation $pO_2/FiO_2 < 150$ ($SpO_2/FiO_2 < 200$) or use of vasopressors	8
	Mechanical ventilation $pO_2/FiO_2 < 150$ and use of vasopressors, dialysis, or extracorporeal membrane oxygenation (ECMO))	9
Death	Death	10

FiO_2=fraction of inspired oxygen, pO_2=partial pressure of oxygen. SpO_2=oxygen saturation. * If the hospitalization is due to quarantine alone, the status is to be recorded as a non-bedridden patient.

[1] Marshall JC, Murthy S, Diaz J, et al. A minimal common outcome measure set for COVID-19

clinical research. Lancet Infet Dis 2020;20:e192-97.

Clinical Classification of COVID-19 issued by National Health Commission of the People's Republic of China

(I) Mild

The clinical symptoms are mild, and there are no radiological findings of pneumonia.

(II) General

Showing fever and respiratory symptoms with radiological findings of pneumonia.

(III) Severe

Adult cases meeting any of the following criteria:

1) Respiratory distress, RR≥30/min;

2) Oxygen saturation ≤93% at resting;

3) Arterial partial pressure of oxygen (PaO2)/fraction of inspired oxygen (FiO2)≤ 300mmHg (1 mmHg=0.133kPa); in high-altitude areas (at an altitude of over 1,000 meters above sea level), PaO2/FiO2 shall be corrected by the following formula: PaO2/FiO2×[760/atmospheric pressure (mmHg)].

4) Cases with progressive severe clinical symptoms and chest imaging that shows obvious lesion progression within 24-48 hours >50%.

(IV) Critical

Cases meeting any of the following criteria:

1) Respiratory failure requiring mechanical ventilation;

2) Shock;

3) Organ failures requiring ICU care.

2.7 Omicron symptom index (OSI)

Calculation method of OSI:

In the confirmed end point case, if any of the following symptoms was present and persisted for ≥2 days (except for dyspnea, there was no persistence request for dyspnea), each symptom was calculated as 1 point:

fever (oral temperature≥ 38.0℃ or ear temperature ≥ 38 ℃ or axillary temperature≥ 37.8℃); sore throat; weakness generalized/fatigue (if two symptoms appear simultaneously, only one symptom is counted); rhinitis; myalgia; headache; lack of appetite/nausea/vomiting (if three symptoms appear simultaneously, only one symptom is counted); diarrhea; mental status changes; cough; loss of taste or smell; dyspnea. OSI was the total number of symptoms mentioned above with a range from 1 to 12 points.

3. Supplementary Figures

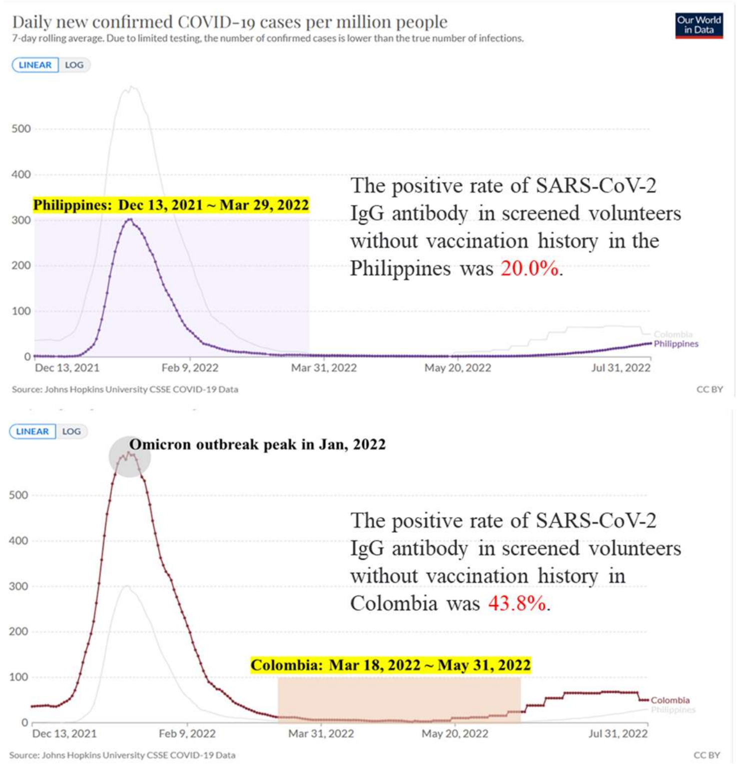

Data source (CC-BY-4.0): https://ourworldindata.org/

Figure S1. The prevalence of COVID-19 in the screening period.

Shown are the daily new confirmed COVID-19 cases per million people during the screening in the Philippines and Colombia, where the participants without COVID-19 vaccination history were allowed to participate in the trial by national regulators. The first Omicron epidemic in the Philippines occurred about half a month after screening was initiated (December 13, 2021), and the epidemic peaked in mid-January, ending more than a month before screening ended (March 29, 2022). The positive rate of SARS-CoV-2 antibodies in screened volunteers without vaccination history in the Philippines was 20.0%. Unlike the Philippines, Colombia had already experienced a very severe COVID-19 epidemic before the initiation of the trial, with a much higher peak than the Philippines. The screening in Colombia (March 18 to May 31, 2022) was initiated after the epidemic had almost completely ended, when it was already difficult to find uninfected individuals in Colombia, and the positive rate of SARS-CoV-2 antibodies in screening volunteers with no vaccination history was as high as 43.8%.

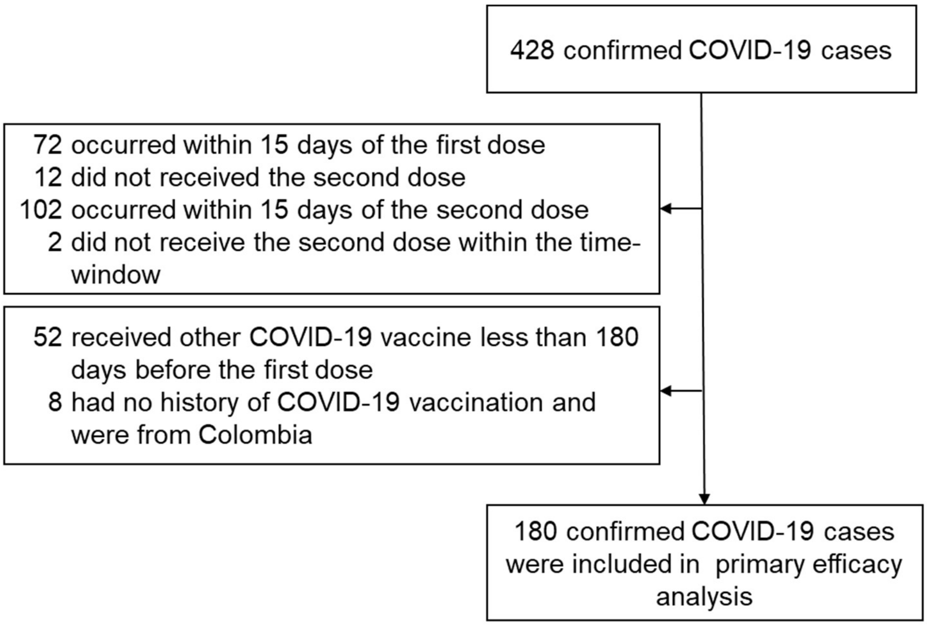

Figure S2. 180 primary end point COVID-19 cases.

During the efficacy observation period, a total of 428 COVID-19 cases were identified, of which 72 had an onset less than 15 days after the first dose, 12 did not receive the second dose, 102 had an onset less than 15 days after the second dose, 2 received the second dose outside the time-window specified in the protocol, 52 were from participants who received their last shot of other COVID-19 vaccine less than 180 days before the enrolment and 8 were participants without COVID-19 vaccination history from Colombia; there were 180 end point cases for the primary efficacy analysis. The exclusion of 240 confirmed cases followed the prespecified definition of per-protocol population or primary end point cases in the protocol, and 8 confirmed cases were excluded because the participants without COVID-19 vaccination history from Colombia were excluded from the per-protocol population, which were jointly determined by the investigators and the sponsor team based on the following reasons: 1) The very small sample size of such participants in Colombia (a total of 389 who received at least one dose (five of them did not receive the second dose), less than 3% of the total population without COVID-19 vaccination history in this trial, all the rest came from the Philippines). The 389 participants without COVID-19 vaccination history were enrolled from eight centers, with sample size ranged from 4 to 159. Based on our extensive clinical trial experience, we found that in situations where the incidence of target disease was extremely high and would fluctuate

violently but the trial sample size is small, there is a higher risk of additional uncertainties and confounding factors between the treatment groups. Furthermore, the independent randomization conducted at each center might lead to an uneven distribution of participant characteristics across the groups (vaccine or placebo) due to the limited sample sizes. 2) The possibility of false negative SARS-CoV-2 antibodies results at baseline. First, the existing SARS-CoV-2 antibody rapid screening reagents are developed based on the prototype virus, and have demonstrated decreased sensitivity in detecting antibodies induced by an Omicron infection. Second, there was a very huge Omicron outbreak peak shortly prior to the enrolment, which resulted in a high seroprevalence of SARS-CoV-2 antibodies during screening and a reduced negative predictive value of rapid antibody screening, i.e., a higher proportion of false-negative in those who tested negative. Taking all these factors into consideration, it was determined that these data may compromise the reliability of the analysis results. To strengthen the robustness of the findings, the decision was made to exclude these participants from primary efficacy analysis (i.e., out of a total of 389 Colombian enrolled participants, except for the exclusion of five who did not receive the second dose, the remaining 384 participants were also excluded from all efficacy analyses for the reasons mentioned above).

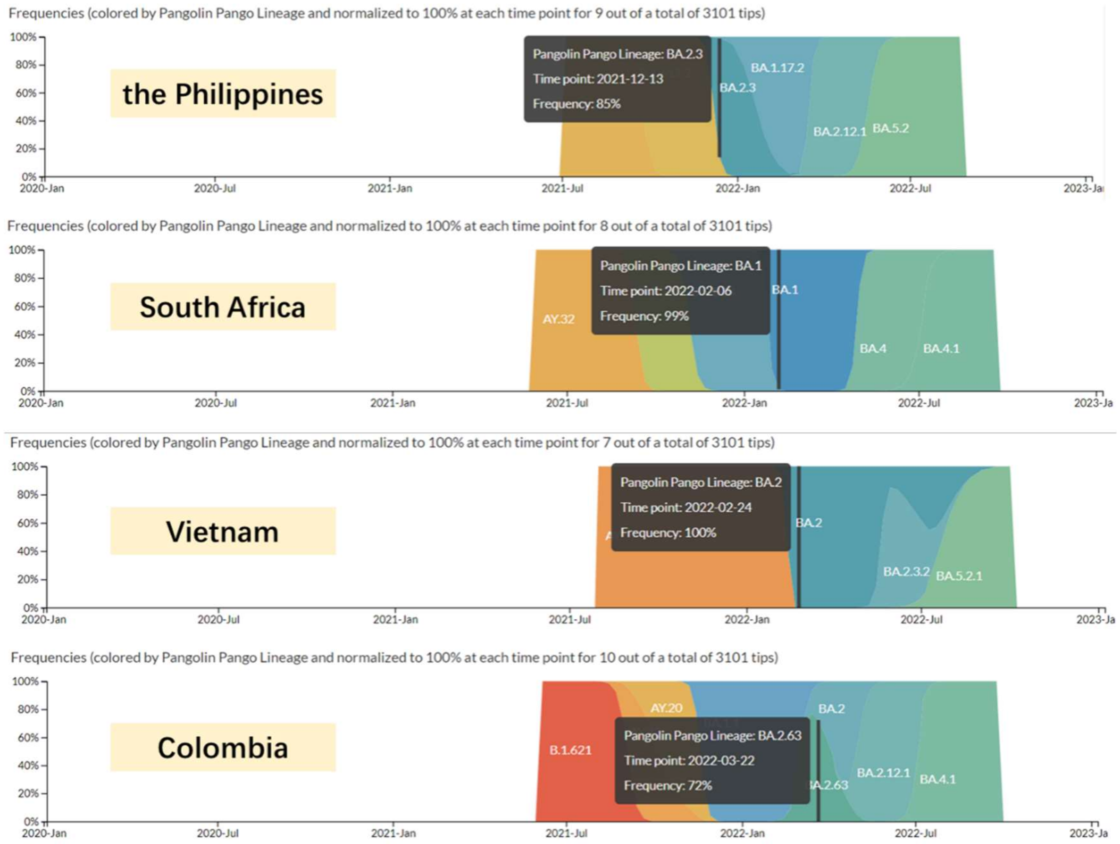

Data source (CC-BY-4.0): https://nextstrain.org/sars-cov-2/

Figure S3. The SARS-CoV-2 variant sublineages circulating in the study regions during the trial.

This phase 3 trial was conducted in the Philippines, South Africa, Vietnam and Colombia. At the launch of this trial (the dates of first-patient-in (FPI) for four countries were Dec 16, 2021, Feb 8, Mar 2 and Mar 22, 2022, respectively), these four countries were all dominated by omicron variant: more than 85% in the Philippines, and 100% in the other three countries. For the variant lineages, from the date of FPI to July 31, 2022, the dominant sublineages in the Philippines were BA.2.3, BA.1.17.2, BA.2.12.1 and BA.5.2, in South Africa were BA.1, BA.4 and BA.4.1, in Vietnam were BA.2, BA.2.3.2 and BA.5.2.1 and in Colombia were BA.2.63, BA.2, BA.2.12.1, and BA.4.1, respectively.

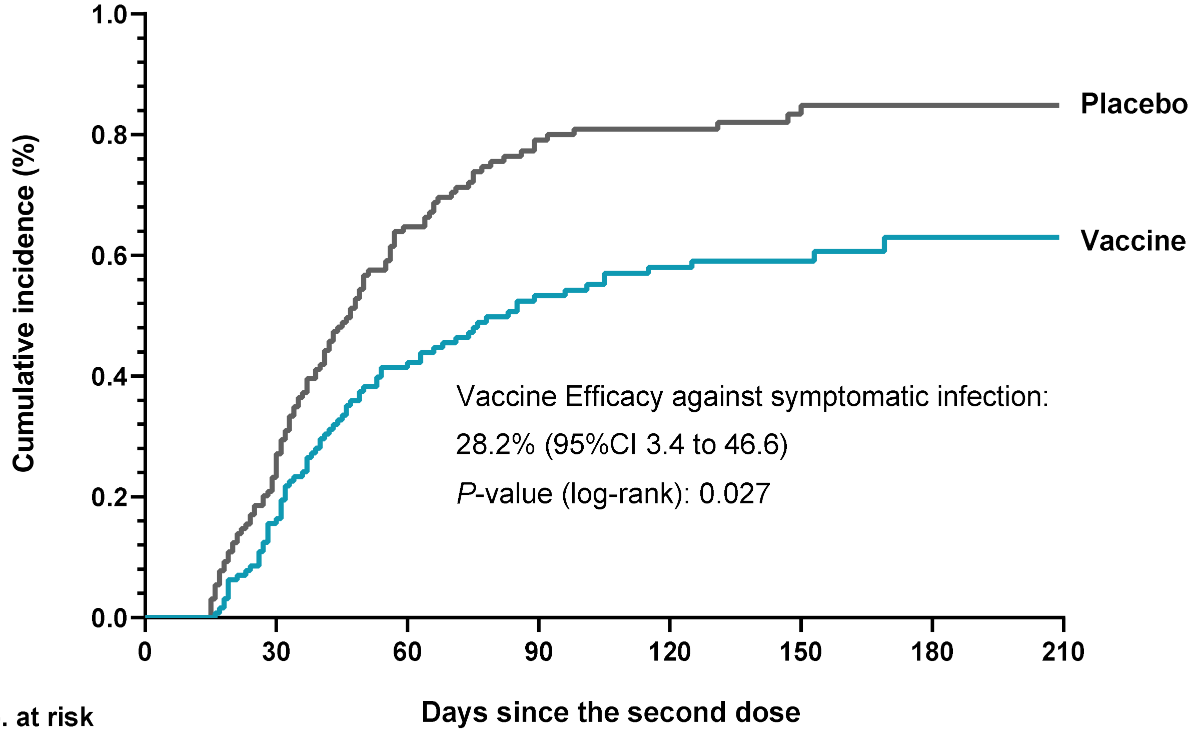

No. at risk

Placebo	12902	12861	12324	11009	10131	6881	2805
Vaccine	12840	12811	12294	11018	10146	6932	2810

Figure S4. The cumulative Incidence of symptomatic SARS-CoV-2 infections in per-protocol population.

Shown is the cumulative incidence of symptomatic SARS-CoV-2 infections during the efficacy observation period in per-protocol population.

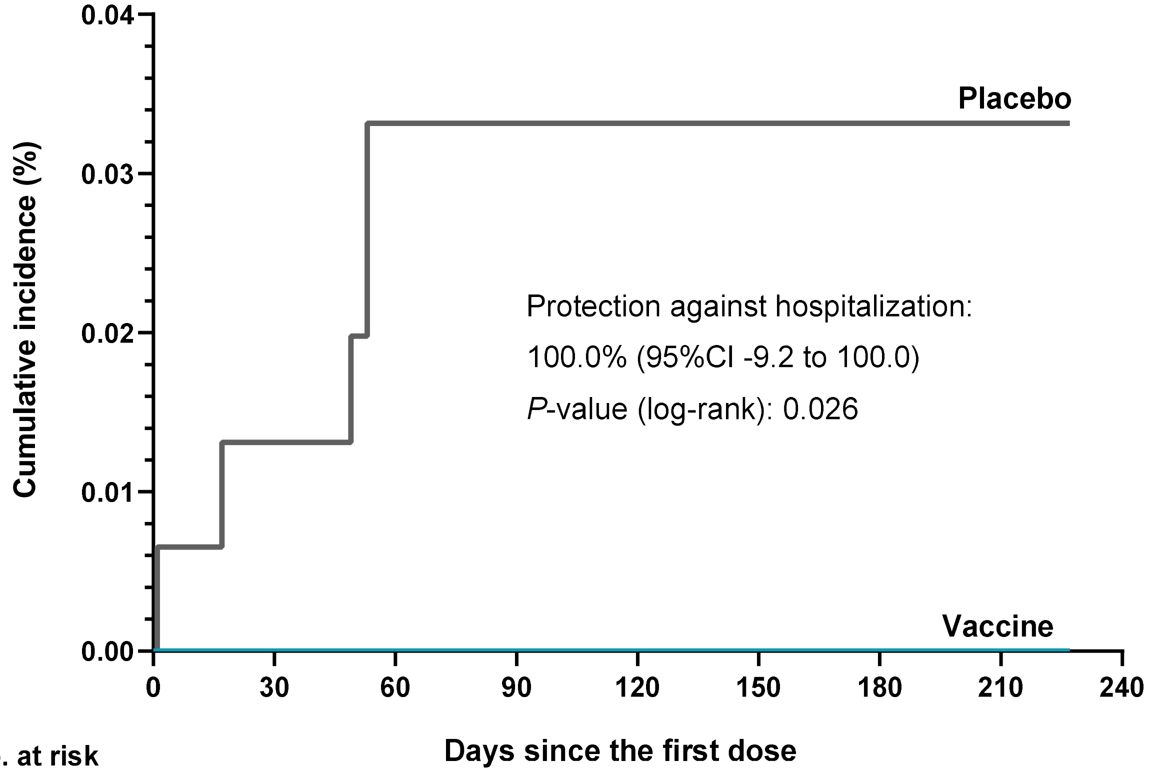

No. at risk

Placebo	15494	15266	15101	13913	11050	8974	5226	1812
Vaccine	15496	15233	15088	13877	11039	8998	5238	1829

Figure S5. Incidence of hospitalized SARS-CoV-2 infection (WHO score ≥ 4) in the participants who received at least one dose.

Shown is the cumulative incidence of hospitalized SARS-CoV-2 infection during the efficacy observation period with a WHO score of 4 or above (3 of which were severe cases according to NMPA criteria) judged and scored by the independent endpoint adjudication committee, in the participants who received at least one dose.

4. Supplementary Tables

Table S1. The distribution of the prior COVID-19 vaccination history in participants who received at least one dose.

	the Philippines	South Africa	Colombia	Vietnam	Total
Participants without COVID-19 vaccination history	13425	-*	389	-*	13814
Participants with COVID-19 vaccination history	7212	2997	3111	3856	17176
Inactivated vaccine	4612	0	531	748	5891
Adenovirus vector vaccine	1195	963	1407	761	4326
mRNA vaccine	1244	1846	1165	405	4660
Recombinant subunit vaccine	156	187	0	10	353
Mixed	3	1	8	1932	1944
Unknown	2	0	0	0	2
Total	20637	2997	3500	3856	30990

*South Africa and Vietnam only enrolled participants with COVID-19 vaccination history.

Table S2. Number of participants who received at least one dose at each center in the four countries.

Site Number	Vaccine Group		Placebo Group		Total	
	N	%	N	%	N	%
Philippines						
1201	425	2.7%	424	2.7%	849	2.7%
1202	1570	10.1%	1571	10.1%	3141	10.1%
1203	753	4.9%	755	4.9%	1508	4.9%
1204	1390	9.0%	1394	9.0%	2784	9.0%
1205	1002	6.5%	1000	6.5%	2002	6.5%
1206	342	2.2%	341	2.2%	683	2.2%
1207	875	5.6%	874	5.6%	1749	5.6%
1208	291	1.9%	293	1.9%	584	1.9%
1210	796	5.1%	798	5.2%	1594	5.1%
1211	1778	11.5%	1779	11.5%	3557	11.5%
1213	602	3.9%	599	3.9%	1201	3.9%
1214	492	3.2%	493	3.2%	985	3.2%
South Africa						
1301	347	2.2%	345	2.2%	692	2.2%
1303	248	1.6%	246	1.6%	494	1.6%
1304	116	0.7%	115	0.7%	231	0.7%
1305	0	0.0%	1	0.0%	1	0.0%
1306	24	0.2%	26	0.2%	50	0.2%
1307	307	2.0%	307	2.0%	614	2.0%
1308	203	1.3%	203	1.3%	406	1.3%
1309	253	1.6%	256	1.7%	509	1.6%
Colombia						
1901	150	1.0%	150	1.0%	300	1.0%
1902	64	0.4%	62	0.4%	126	0.4%
1903	41	0.3%	40	0.3%	81	0.3%
1904	152	1.0%	149	1.0%	301	1.0%
1905	534	3.4%	535	3.5%	1069	3.4%
1906	695	4.5%	692	4.5%	1387	4.5%
1907	48	0.3%	48	0.3%	96	0.3%
1908	70	0.5%	70	0.5%	140	0.5%
Vietnam						
2301	439	2.8%	436	2.8%	875	2.8%
2302	936	6.0%	941	6.1%	1877	6.1%
2303	69	0.4%	70	0.5%	139	0.4%
2304	4	0.0%	3	0.0%	7	0.0%
2305	480	3.1%	478	3.1%	958	3.1%
Total	15496	100.0%	15494	100.0%	30990	100.0%

Table S3. The duration of follow-up and the dropout rate during the efficacy observation period in participants who received at least one dose.

		Total	Vaccine group	Placebo group	P-value
Follow-up duration	N	30990	15496	15494	
	Mean (SD)	152.8 (47.84)	152.8 (48.04)	152.8 (47.65)	0.8682*
	range	1, 228	1, 228	1, 228	
	Median	161	161	161	
	IQR	111.0-189.0	111.0-189.0	111.0-189.0	
Dropout	n(%)	668 (2.2%)	337 (2.2%)	331 (2.1%)	0.8158#

N: the number of participants who received at least one dose of the vaccine or placebo. SD: standard deviation. IQR: interquartile range.

*The T-test was used to assess the difference between the vaccine group and the placebo group for each continuous variable.

Chi-square test was used to analyze the lost-follow-up rate of the two groups.

Table S4. Demographics and baseline characteristics in per-protocol population

		Vaccine group (n=12840)	Placebo group (n=12902)
Age (years)			
	Mean (SD)	39·7 (15·5)	39·8 (15·5)
	Median (IQR)	37·0 (27·0, 52·0)	37·0 (27·0, 51·0)
	Min, Max	18, 89	18, 91
Age group, n(%)			
	18-59 years	10930 (85·1)	10978 (85·1)
	>=60 years	1910 (14·9)	1924 (14·9)
Sex, n(%)			
	Male	6572 (51·2)	6508 (50·4)
	Female	6268 (48·8)	6393 (49·6)
	Undifferentiated	0	1 (0·0)
Country, n(%)			
	the Philippines	10014 (78.0)	10030 (77.7)
	South Africa	1295 (10.1)	1307 (10.1)
	Colombia	1465 (11.4)	1472 (11.4)
	Vietnam	66 (0.5)	93(0.7)
Race, n(%)			
	Asian	10080 (78·5)	10123 (78·5)
	White	20 (0·2)	18 (0·1)
	Black or African American	954 (7·4)	978 (7·6)
	American Indian or Alaska Native	107 (0·8)	120 (0·9)
	Multiple	1679 (13·1)	1663 (12·9)
BMI (kg/m^2)			
	Mean (SD)	24.8 (5·4)	24.8 (5·4)
Underlying chronic condition†, n(%)			
	Yes	1929 (15·0)	1885 (14·6)
	No	10911 (85·0)	11017 (85·4)
SARS-CoV-2 antibody status, n(%)			
	Negative	10143 (79·0)	10118 (78·4)
	Positive	2689 (20·9)	2777 (21·5)
	Missing data	8	7
COVID-19 vaccination history		6332	6385
Time since the last priming dose of other COVID-19 vaccine (Days)			
	Median (IQR)	209·0 (195·0, 235·0)	209·0 (195·0, 235·0)
COVID-19 vaccine types, n(%)			
	Inactivated vaccine	2518 (39·8)	2543 (39·8)
	Adenovirus vector vaccine	1719 (27·1)	1700 (26·6)
	mRNA vaccine	1922 (30·4)	1954 (30·6)
	Recombinant subunit vaccine	162 (2·6)	179 (2·8)
	Mixed	10 (0·2)	8 (0·1)

Percentages may not total 100 because of rounding. SD denotes standard deviation, IQR interquartile range, SARS-

CoV-2 severe acute respiratory syndrome coronavirus 2.

†Underlying chronic conditions were those that were ongoing at baseline and could increase the risk of SARS-CoV-2 infection.

Table S5. The number of doses of prior COVID-19 vaccination.

The number of doses		Vaccine group (N=8586)	Placebo group (N=8590)	Total (N=17176)
All participants with COVID-19 vaccination history				
	1	1787 (20.8)	1813 (21.1)	3600 (21.0)
	2	5301 (61.7)	5303 (61.7)	10604 (61.7)
	3	1496 (17.4)	1471 (17.1)	2967 (17.3)
	4	2 (0.0)	3 (0.0)	5 (0.0)
Vaccine type				
Inactivated vaccine				
	1	108 (1.3)	100 (1.2)	208 (1.2)
	2	2678 (31.2)	2693 (31.4)	5371 (31.3)
	3	139 (1.6)	173 (2.0)	312 (1.8)
	4	0	0	0
Adenovirus vector vaccine				
	1	1391 (16.2)	1398 (16.3)	2789 (16.2)
	2	525 (6.1)	483 (5.6)	1008 (5.9)
	3	268 (3.1)	259 (3.0)	527 (3.1)
	4	1 (0.0)	1 (0.0)	2 (0.0)
mRNA vaccine				
	1	204 (2.4)	222 (2.6)	426 (2.5)
	2	1961 (22.8)	1969 (22.9)	3930 (22.9)
	3	170 (2.0)	134 (1.6)	304 (1.8)
	4	0	0	0
Recombinant subunit vaccine				
	1	83 (1.0)	92 (1.1)	175 (1.0)
	2	80 (0.9)	87 (1.0)	167 (1.0)
	3	7 (0.1)	3 (0.0)	10 (0.1)
	4	0	1 (0.0)	1 (0.0)
Mixed				
	1	0	0	0
	2	57 (0.7)	71 (0.8)	128 (0.8)
	3	912 (10.6)	902 (10.5)	1814 (10.6)
	4	1 (0.0)	1 (0.0)	2 (0.0)

The analysis was based on the participants who received at least one dose, grouped according to randomization assignment. Percentages may not total 100 because of rounding.

Table S6. Local and systemic solicited adverse reactions that occurred within 7 days after any dose in safety analysis set.

Symptoms	Participants without COVID-19 vaccination history				Participants with COVID-19 vaccination history				Total			
	Vaccine group (n=6910)	Placebo group (n=6904)	Rate difference (95% CI)	*P* value	Vaccine group (n=8590)	Placebo group (n=8586)	Rate difference (95% CI)	*P* value	Vaccine group (n=15500)	Placebo group (n=15490)	Rate difference (95% CI)	*P* value
Local symptoms												
Any	251 (3.6)	265 (3.8)	-0.21 (-0.93, 0.52)	0.52	589 (6.9)	564 (6.6)	0.29 (-0.57, 1.14)	0.45	840 (5.4)	829 (5.4)	0.07 (-0.51, 0.64)	0.79
Nasal obstruction	61 (0.9)	50 (0.7)	0.16 (-0.18, 0.50)	0.30	289 (3.4)	247 (2.9)	0.49 (-0.11, 1.08)	0.07	350 (2.3)	297 (1.9)	0.34 (-0.02, 0.70)	0.04
Rhinorrhea	187 (2.7)	183 (2.7)	0.06 (-0.56, 0.67)	0.84	391 (4.6)	363 (4.2)	0.32 (-0.38, 1.02)	0.30	578 (3.7)	546 (3.5)	0.20 (-0.27, 0.68)	0.34
Sore throat	72 (1.0)	95 (1.4)	-0.33 (-0.75, 0.08)	0.07	252 (2.9)	242 (2.8)	0.12 (-0.46, 0.69)	0.65	324 (2.1)	337 (2.2)	-0.09 (-0.45, 0.28)	0.60
Systemic symptoms												
Any	612 (8.9)	642 (9.3)	-0.44 (-1.54, 0.65)	0.37	1099 (12.8)	1086 (12.6)	0.15 (-0.99, 1.29)	0.77	1711 (11.0)	1728 (11.2)	-0.12 (-0.92, 0.68)	0.74
Fever	248 (3.6)	247 (3.6)	0.01 (-0.70, 0.72)	0.97	211 (2.5)	227 (2.6)	-0.19 (-0.73, 0.35)	0.44	459 (3.0)	474 (3.1)	-0.10 (-0.53, 0.34)	0.61
Cough	188 (2.7)	184 (2.7)	0.06 (-0.56, 0.67)	0.84	298 (3.5)	342 (4.0)	-0.51 (-1.16, 0.13)	0.08	486 (3.1)	526 (3.4)	-0.26 (-0.71, 0.19)	0.20
Acute allergic reaction	20 (0.3)	19 (0.3)	0.01 (-0.19, 0.22)	0.87	33 (0.4)	35 (0.4)	-0.02 (-0.24, 0.19)	0.81	53 (0.3)	54 (0.3)	-0.01 (-0.16, 0.14)	0.92
Headache	228 (3.3)	255 (3.7)	-0.39 (-1.09, 0.31)	0.21	601 (7.0)	542 (6.3)	0.68 (-0.17, 1.54)	0.07	829 (5.3)	797 (5.1)	0.20 (-0.36, 0.77)	0.42
Fatigue and weakness	126 (1.8)	135 (2.0)	-0.13 (-0.65, 0.39)	0.57	333 (3.9)	338 (3.9)	-0.06 (-0.72, 0.60)	0.84	459 (3.0)	473 (3.1)	-0.09 (-0.53, 0.34)	0.63

Nausea	83 (1.2)	90 (1.3)	-0.1 (-0.53, 0.32)	0.59	142 (1.7)	130 (1.5)	0.14 (-0.29, 0.57)	0.47	225 (1.5)	220 (1.4)	0.03 (-0.27, 0.33)	0.82
Vomiting	29 (0.4)	37 (0.5)	-0.12 (-0.38, 0.15)	0.32	48 (0.6)	35 (0.4)	0.15 (-0.09, 0.39)	0.15	77 (0.5)	72 (0.5)	0.03 (-0.14, 0.21)	0.68
Diarrhea	102 (1.5)	101 (1.5)	0.01 (-0.45, 0.47)	0.95	202 (2.4)	224 (2.6)	-0.26 (-0.79, 0.27)	0.28	304 (2.0)	325 (2.1)	-0.14 (-0.50, 0.22)	0.39
Myalgia	83 (1.2)	102 (1.5)	-0.28 (-0.71, 0.16)	0.16	311 (3.6)	276 (3.2)	0.41 (-0.22, 1.03)	0.14	394 (2.5)	378 (2.4)	0.1 (-0.30, 0.50)	0.57

A total of 30,990 participants were included in the safety analysis set, among which 8 participants experienced vaccination errors, 5 of them who were randomly assigned to receive the placebo but instead received at least one dose of vaccine were included in the vaccine group, while one of them who was assigned to receive the vaccine but instead received two doses of placebo was included in the placebo group. Data of incidence are shown as n (%). n, number of participants; %, proportion of participants; any, all participants with any grade of adverse reactions.

Table S7. Adverse events or reactions that occurred after any dose in safety analysis set, stratified according to age group.

	Participants aged 18-59 years				Participants aged ≥ 60 years			
	Vaccine group (n=13219)	Placebo group (n=13214)	Rate difference (95% CI)	*P* value	Vaccine group (n=2281)	Placebo group (n=2276)	Rate difference (95% CI)	*P* value
Adverse events within 30 d, n(%)								
Any	2079 (15.7)	2109 (16.0)	-0.23 (-1.24, 0.77)	0.60	344 (15.1)	307 (13.5)	1.59 (-0.73, 3.92)	0.12
≥ Grade 3	111 (0.8)	100 (0.8)	0.08 (-0.16, 0.33)	0.45	22 (1.0)	12 (0.5)	0.44 (-0.13, 1.01)	0.09
Adverse reactions within 30 d, n(%)								
Any	1665 (12.6)	1700 (12.9)	-0.27 (-1.19, 0.65)	0.51	259 (11.4)	224 (9.8)	1.51 (-0.53, 3.56)	0.10
≥ Grade 3	74 (0.6)	66 (0.5)	0.06 (-0.14, 0.26)	0.50	11 (0.5)	4 (0.2)	0.31 (-0.07, 0.69)	0.07
Solicited adverse reactions within 7 d, n(%)								
Any	1644 (12.4)	1668 (12.6)	-0.19 (-1.10, 0.73)	0.65	251 (11.0)	219 (9.6)	1.38 (-0.64, 3.40)	0.13
≥ Grade 3	73 (0.6)	65 (0.5)	0.06 (-0.14, 0.26)	0.50	10 (0.4)	4 (0.2)	0.26 (-0.10, 0.63)	0.11
Local symptoms								
Any	738 (5.6)	739 (5.6)	-0.01 (-0.64, 0.62)	0.97	102 (4.5)	90 (4.0)	0.52 (-0.82, 1.85)	0.38
≥ Grade 3	0	0	NA	-	1 (0.0)	0	0.04 (-0.05, 0.14)	> 0.99
Systemic symptoms								
Any	1484 (11.2)	1527 (11.6)	-0.33 (-1.21, 0.55)	0.40	227 (10.0)	201 (8.8)	1.12 (-0.82, 3.06)	0.19
≥ Grade 3	73 (0.6)	65 (0.5)	0.06 (-0.14, 0.26)	0.50	10 (0.4)	4 (0.2)	0.26 (-0.10, 0.63)	0.11
Serious adverse events throughout the observation period, n(%)								
Any	116 (0.9)	107 (0.8)	0.07 (-0.18, 0.32)	0.55	44 (1.9)	47 (2.1)	-0.14 (-1.07, 0.79)	0.74
Vaccination-related	0	0	NA	-	0	0	NA	-
Medically attended adverse events, n(%)								
Any	344 (2.6)	337 (2.6)	0.05 (-0.38, 0.49)	0.79	76 (3.3)	84 (3.7)	-0.36 (-1.58, 0.86)	0.51

≥ Grade 3	101 (0.8)	94 (0.7)	0.05 (-0.18, 0.29)	0.62	33 (1.4)	31 (1.4)	0.08 (-0.07, 0.87)	0.81
Adverse events of special interest, n(%)								
Any	70 (0.5)	67 (0.5)	0.02 (-0.18, 0.22)	0.80	6 (0.3)	11 (0.5)	-0.22 (-0.63, 0.18)	0.22
≥ Grade 3	9 (0.1)	5 (0.0)	0.03 (-0.03, 0.09)	0.29	5 (0.2)	6 (0.3)	-0.04 (-0.37, 0.28)	0.76

The analysis was based on the participants in the safety analysis set, grouped according to actual intervention. Participants who received at least one dose of Pneucolin® were included in the vaccine group. Data of incidence are shown as n (%). n, number of participants; %, proportion of participants; any, all participants with any grade of adverse events or reactions.

Table S8. Adverse events or reactions that occurred after any dose in safety analysis set, stratified according to the presence of underlying chronic condition* at baseline.

	Participants with underlying chronic conditions				Participants without underlying chronic conditions			
	Vaccine group (n=2252)	Placebo group (n=2189)	Rate difference (95% CI)	*P* value	Vaccine group (n=13248)	Placebo group (n=13301)	Rate difference (95% CI)	*P* value
Adverse events within 30 d, n(%)								
Any	433 (19.2)	377 (17.2)	2.00 (-0.59, 4.60)	0.08	1990 (15.0)	2039 (15.3)	-0.31 (-1.30, 0.68)	0.48
≥ Grade 3	29 (1.3)	24 (1.1)	0.19 (-0.54, 0.92)	0.56	104 (0.8)	88 (0.7)	0.12 (-0.11, 0.36)	0.24
Adverse reactions within 30 d, n(%)								
Any	332 (14.7)	290 (13.2)	1.49 (-0.84, 3.83)	0.15	1592 (12.0)	1634 (12.3)	-0.27 (-1.17, 0.63)	0.50
≥ Grade 3	13 (0.6)	10 (0.5)	0.12 (-0.36, 0.60)	0.58	72 (0.5)	60 (0.5)	0.09 (-0.10, 0.29)	0.28
Solicited adverse reactions within 7 d, n(%)								
Any	324 (14.4)	284 (13.0)	1.41 (-0.90, 3.72)	0.17	1571 (11.9)	1603 (12.1)	-0.19 (-1.09, 0.70)	0.63
≥ Grade 3	13 (0.6)	10 (0.5)	0.12 (-0.36, 0.60)	0.58	70 (0.5)	59 (0.4)	0.08 (-0.11, 0.28)	0.32
Local symptoms								
Any	164 (7.3)	127 (5.8)	1.48 (-0.18, 3.14)	0.05	676 (5.1)	702 (5.3)	-0.18 (-0.79, 0.44)	0.52
≥ Grade 3	1 (0.0)	0	0.04 (-0.06, 0.14)	> 0.99	0	0	NA	-
Systemic symptoms								
Any	296 (13.1)	259 (11.8)	1.31 (-0.91, 3.53)	0.19	1415 (10.7)	1469 (11.0)	-0.36 (-1.22, 0.49)	0.34
≥ Grade 3	13 (0.6)	10 (0.5)	0.12 (-0.36, 0.60)	0.58	70 (0.5)	59 (0.4)	0.08 (-0.11, 0.28)	0.32
Serious adverse events throughout the observation period, n(%)								
Any	52 (2.3)	54 (2.5)	-0.16 (-1.19, 0.87)	0.73	108 (0.8)	100 (0.8)	0.06 (-0.18, 0.31)	0.56
Vaccination-related	0	0	NA	-	0	0	NA	-
Medically attended adverse events, n(%)								
Any	114 (5.1)	109 (5.0)	0.08 (-1.39, 1.55)	0.90	306 (2.3)	312 (2.3)	-0.04 (-0.45, 0.38)	0.85
≥ Grade 3	41 (1.8)	45 (2.1)	-0.24 (-1.16, 0.69)	0.57	93 (0.7)	80 (0.6)	0.10 (-0.12, 0.32)	0.31

Adverse events of special interest, n(%)								
Any	13 (0.6)	11 (0.5)	0.07 (-0.42, 0.57)	0.73	63 (0.5)	67 (0.5)	-0.03 (-0.22, 0.16)	0.74
≥ Grade 3	6 (0.3)	7 (0.3)	-0.05 (-0.42, 0.31)	0.74	8 (0.1)	4 (0.0)	0.03 (-0.03, 0.09)	0.25

The analysis was based on the participants in the safety analysis set, grouped according to actual intervention. Participants who received at least one dose of Pneucolin® were included in the vaccine group. Data of incidence are shown as n (%). n, number of participants; %, proportion of participants; any, all participants with any grade of adverse events or reactions. *Underlying chronic conditions were those that were ongoing at baseline and could increase the risk of SARS-CoV-2 infection.

Table S9. Adverse events or reactions that occurred after any dose in participants with underlying respiratory disease or nose-related diseases at baseline in safety analysis set.

	Participants with underlying respiratory diseases*				Participants with underlying nose-related diseases[†]			
	Vaccine group (n=121)	Placebo group (n=112)	Rate difference (95% CI)	*P* value	Vaccine group (n=80)	Placebo group (n=73)	Rate difference (95% CI)	*P* value
Adverse events within 30 d, n(%)								
Any	40 (33.1)	44 (39.3)	-6.23 (-20.33, 7.87)	0.32	41 (51.3)	44 (60.3)	-9.02 (-26.96, 8.91)	0.26
≥ Grade 3	4 (3.3)	0	3.31 (-0.34, 6.95)	0.12	0	2 (2.7)	-2.74 (-7.02, 1.54)	0.23
Adverse reactions within 30 d, n(%)								
Any	26 (21.5)	37 (33.0)	-11.55 (-24.56, 1.46)	0.05	33 (41.3)	36 (49.3)	-8.07 (-26.07, 9.94)	0.32
≥ Grade 3	0	0	NA	-	0	1 (1.4)	-1.37 (-4.42, 1.68)	0.48
Solicited adverse reactions within 7 d, n(%)								
Any	25 (20.7)	36 (32.1)	-11.48 (-24.36, 1.4)	0.05	33 (41.3)	36 (49.3)	-8.07 (-26.07, 9.94)	0.32
≥ Grade 3	0	0	NA	-	0	1 (1.4)	-1.37 (-4.42, 1.68)	0.48
Local symptoms								
Any	13 (10.7)	15 (13.4)	-2.65 (-12.23, 6.93)	0.53	20 (25.0)	26 (35.6)	-10.62 (-27.22, 5.98)	0.15
≥ Grade 3	0	0	NA	-	0	0	NA	-
Systemic symptoms								
Any	21 (17.4)	32 (28.6)	-11.22 (-23.51, 1.08)	0.04	25 (31.3)	31 (42.5)	-11.22 (-28.62, 6.19)	0.15
≥ Grade 3	0	0	NA	-	0	1 (1.4)	-1.37 (-4.42, 1.68)	0.48
Serious adverse events throughout the observation period, n(%)								
Any	8 (6.6)	1 (0.9)	5.72 (0.28, 11.16)	0.04	3 (3.8)	2 (2.7)	1.01 (-5.39, 7.41)	> 0.99
Vaccination-related	0	0	NA	-	0	0	NA	-
Medically attended adverse events, n(%)								
Any	20 (16.5)	10 (8.9)	7.60 (-2.08, 17.28)	0.08	7 (8.8)	11 (15.1)	-6.32 (-18.08, 5.44)	0.23
≥ Grade 3	7 (5.8)	1 (0.9)	4.89(-0.27, 10.05)	0.07	3 (3.8)	3 (4.1)	-0.36 (-7.42, 6.70)	> 0.99
Adverse events of special interest, n(%)								

Any	0	0	NA	-	0	2 (2.7)	-2.74 (-7.02, 1.54)	0.23
≥ Grade 3	0	0	NA	-	0	0	NA	-

Data of incidence are shown as n (%). n, number of participants; %, proportion of participants; any, all participants with any grade of adverse events or reactions. Adverse events or reactions are graded according to the relevant guidance of the China National Medical Products Administration (NMPA). * Underlying respiratory disease includes asthma, chronic obstructive pulmonary disease, sleep apnea syndrome, bronchitis, etc. † Underlying nose-related diseases includes rhinitis or allergic rhinitis, nasosinusitis, deviation of nasal septum, nasal polyp, etc.

Table S10. Medically attended adverse events in safety analysis set throughout the observation period.

Preferred Term	Vaccine group (n=15500) n(%) m	Placebo group (n=15490) n(%) m	Rate difference (95% CI)	*P* value
Type 2 diabetes mellitus	4 (0.03) 4	2 (0.01) 2	0.01 (-0.02, 0.05)	0.69
Insulin-requiring type 2 diabetes mellitus	0	1 (< 0.01) 1	-0.01 (-0.02, 0.01)	0.50
Hypoglycaemia	0	1 (< 0.01) 1	-0.01 (-0.02, 0.01)	0.50
Hypokalaemia	0	3 (0.02) 3	-0.02 (-0.04, 0.01)	0.12
Electrolyte imbalance	1 (< 0.01) 1	1 (< 0.01) 1	0.00 (-0.02, 0.02)	> 0.99
Gout	1 (< 0.01) 1	1 (< 0.01) 1	0.00 (-0.02, 0.02)	> 0.99
Diabetes mellitus	0	1 (< 0.01) 1	-0.01 (-0.02, 0.01)	0.50
Diabetic metabolic decompensation	0	2 (0.01) 3	-0.01 (-0.03, 0.01)	0.25
Dehydration	0	1 (< 0.01) 1	-0.01 (-0.02, 0.01)	0.50
Glucose tolerance impaired	0	2 (0.01) 2	-0.01 (-0.03, 0.01)	0.25
Dyslipidaemia	2 (0.01) 2	2 (0.01) 2	0.00 (-0.03, 0.03)	> 0.99
Decreased appetite	1 (< 0.01) 1	0	0.01 (-0.01, 0.02)	> 0.99
Hypertriglyceridaemia	1 (< 0.01) 1	0	0.01 (-0.01, 0.02)	> 0.99
Hyperlipidaemia	0	2 (0.01) 2	-0.01 (-0.03, 0.01)	0.25
Hyperglycaemia	1 (< 0.01) 1	0	0.01 (-0.01, 0.02)	> 0.99
Hypersensitivity	2 (0.01) 2	0	0.01 (-0.01, 0.03)	0.50
Food allergy	1 (< 0.01) 1	0	0.01 (-0.01, 0.02)	> 0.99
Discomfort	1 (< 0.01) 1	2 (0.01) 2	-0.01 (-0.03, 0.02)	0.62
Asthenia	2 (0.01) 2	2 (0.01) 2	0.00 (-0.03, 0.03)	> 0.99

Pyrexia	6 (0.04) 7	12 (0.08) 15	-0.04 (-0.1, 0.02)	0.16
Oedema peripheral	1 (< 0.01) 1	0	0.01 (-0.01, 0.02)	> 0.99
Multiple organ dysfunction syndrome	0	1 (< 0.01) 1	-0.01 (-0.02, 0.01)	0.50
Sudden cardiac death	1 (< 0.01) 1	0	0.01 (-0.01, 0.02)	> 0.99
Death	1 (< 0.01) 1	1 (< 0.01) 1	0.00 (-0.02, 0.02)	> 0.99
Influenza like illness	1 (< 0.01) 1	0	0.01 (-0.01, 0.02)	> 0.99
Inflammation	0	1 (< 0.01) 1	-0.01 (-0.02, 0.01)	0.50
Sudden death	0	1 (< 0.01) 1	-0.01 (-0.02, 0.01)	0.50
Fatigue	7 (0.05) 7	5 (0.03) 5	0.01 (-0.04, 0.06)	0.56
Pain	1 (< 0.01) 1	0	0.01 (-0.01, 0.02)	> 0.99
Chest pain	3 (0.02) 3	1 (< 0.01) 1	0.01 (-0.02, 0.04)	0.63
Chest discomfort	1 (< 0.01) 1	0	0.01 (-0.01, 0.02)	> 0.99
Malaise	2 (0.01) 2	2 (0.01) 2	0.00 (-0.03, 0.03)	> 0.99
Hypothyroidism	0	1 (< 0.01) 1	-0.01 (-0.02, 0.01)	0.50
Adrenal insufficiency	1 (< 0.01) 1	0	0.01 (-0.01, 0.02)	> 0.99
Trisomy 21	1 (< 0.01) 1	0	0.01 (-0.01, 0.02)	> 0.99
Type V hyperlipidaemia	0	2 (0.01) 2	-0.01 (-0.03, 0.01)	0.25
Prosthesis implantation	0	1 (< 0.01) 1	-0.01 (-0.02, 0.01)	0.50
Hysterectomy	1 (< 0.01) 1	0	0.01 (-0.01, 0.02)	> 0.99
Tooth extraction	1 (< 0.01) 1	0	0.01 (-0.01, 0.02)	> 0.99
Cataract operation	0	1 (< 0.01) 1	-0.01 (-0.02, 0.01)	0.50
Sterilisation	0	1 (< 0.01) 1	-0.01 (-0.02, 0.01)	0.50
Tumour excision	1 (< 0.01) 1	0	0.01 (-0.01, 0.02)	> 0.99

Cholecystectomy	1 (< 0.01) 1	0	0.01 (-0.01, 0.02)	> 0.99
Vasectomy	1 (< 0.01) 1	0	0.01 (-0.01, 0.02)	> 0.99
Joint effusion	0	1 (< 0.01) 1	-0.01 (-0.02, 0.01)	0.50
Arthritis	0	2 (0.01) 2	-0.01 (-0.03, 0.01)	0.25
Arthralgia	2 (0.01) 2	2 (0.01) 2	0.00 (-0.03, 0.03)	> 0.99
Joint swelling	0	1 (< 0.01) 1	-0.01 (-0.02, 0.01)	0.50
Intervertebral disc disorder	0	1 (< 0.01) 1	-0.01 (-0.02, 0.01)	0.50
Intervertebral disc displacement	0	2 (0.01) 2	-0.01 (-0.03, 0.01)	0.25
Intervertebral disc protrusion	0	1 (< 0.01) 1	-0.01 (-0.02, 0.01)	0.50
Rheumatoid arthritis	1 (< 0.01) 1	0	0.01 (-0.01, 0.02)	> 0.99
Costochondritis	2 (0.01) 3	0	0.01 (-0.01, 0.03)	0.50
Myopathy	1 (< 0.01) 1	0	0.01 (-0.01, 0.02)	> 0.99
Myalgia	8 (0.05) 10	10 (0.06) 10	-0.01 (-0.07, 0.05)	0.64
Muscle contracture	1 (< 0.01) 1	0	0.01 (-0.01, 0.02)	> 0.99
Muscle fatigue	0	1 (< 0.01) 1	-0.01 (-0.02, 0.01)	0.50
Rotator cuff syndrome	0	1 (< 0.01) 1	-0.01 (-0.02, 0.01)	0.50
Back pain	3 (0.02) 3	2 (0.01) 2	0.01 (-0.03, 0.04)	> 0.99
Spinal osteoarthritis	1 (< 0.01) 1	1 (< 0.01) 1	0.00 (-0.02, 0.02)	> 0.99
Neck pain	1 (< 0.01) 1	1 (< 0.01) 1	0.00 (-0.02, 0.02)	> 0.99
Osteoarthritis	0	1 (< 0.01) 1	-0.01 (-0.02, 0.01)	0.50
Human bite	1 (< 0.01) 1	0	0.01 (-0.01, 0.02)	> 0.99
Wound	4 (0.03) 4	3 (0.02) 3	0.01 (-0.03, 0.04)	> 0.99
Joint injury	1 (< 0.01) 1	2 (0.01) 2	-0.01 (-0.03, 0.02)	0.62

Joint dislocation	1 (< 0.01) 1	4 (0.03) 4	-0.02 (-0.05, 0.01)	0.22
Incision site inflammation	1 (< 0.01) 1	0	0.01 (-0.01, 0.02)	> 0.99
Animal bite	0	4 (0.03) 4	-0.03 (-0.05, 0)	0.06
Traumatic amputation	1 (< 0.01) 1	0	0.01 (-0.01, 0.02)	> 0.99
Multiple injuries	3 (0.02) 3	3 (0.02) 3	0.00 (-0.04, 0.04)	> 0.99
Multiple fractures	1 (< 0.01) 1	0	0.01 (-0.01, 0.02)	> 0.99
Head injury	1 (< 0.01) 1	1 (< 0.01) 1	0.00 (-0.02, 0.02)	> 0.99
Ulna fracture	1 (< 0.01) 1	1 (< 0.01) 1	0.00 (-0.02, 0.02)	> 0.99
Fractured coccyx	0	1 (< 0.01) 1	-0.01 (-0.02, 0.01)	0.50
Cardiac procedure complication	0	1 (< 0.01) 1	-0.01 (-0.02, 0.01)	0.50
Hand fracture	1 (< 0.01) 1	1 (< 0.01) 1	0.00 (-0.02, 0.02)	> 0.99
Contusion	1 (< 0.01) 1	1 (< 0.01) 1	0.00 (-0.02, 0.02)	> 0.99
Injury	0	2 (0.01) 2	-0.01 (-0.03, 0.01)	0.25
Gun shot wound	1 (< 0.01) 1	2 (0.01) 2	-0.01 (-0.03, 0.02)	0.62
Radius fracture	1 (< 0.01) 1	1 (< 0.01) 1	0.00 (-0.02, 0.02)	> 0.99
Spinal compression fracture	1 (< 0.01) 1	1 (< 0.01) 1	0.00 (-0.02, 0.02)	> 0.99
Thermal burn	0	1 (< 0.01) 1	-0.01 (-0.02, 0.01)	0.50
Overdose	1 (< 0.01) 1	0	0.01 (-0.01, 0.02)	> 0.99
Skin laceration	3 (0.02) 3	1 (< 0.01) 1	0.01 (-0.02, 0.04)	0.63
Skin abrasion	1 (< 0.01) 1	1 (< 0.01) 1	0.00 (-0.02, 0.02)	> 0.99
Eye contusion	1 (< 0.01) 1	0	0.01 (-0.01, 0.02)	> 0.99
Epidural haemorrhage	0	1 (< 0.01) 1	-0.01 (-0.02, 0.01)	0.50
Rib fracture	2 (0.01) 2	0	0.01 (-0.01, 0.03)	0.50

Tendon injury	1 (< 0.01) 1	1 (< 0.01) 1	0.00 (-0.02, 0.02)	> 0.99
Tendon rupture	0	1 (< 0.01) 1	-0.01 (-0.02, 0.01)	0.50
Femur fracture	1 (< 0.01) 1	0	0.01 (-0.01, 0.02)	> 0.99
Limb crushing injury	0	1 (< 0.01) 1	-0.01 (-0.02, 0.01)	0.50
Limb injury	2 (0.01) 2	4 (0.03) 4	-0.01 (-0.05, 0.02)	0.45
Humerus fracture	1 (< 0.01) 1	0	0.01 (-0.01, 0.02)	> 0.99
Tibia fracture	2 (0.01) 2	2 (0.01) 2	0.00 (-0.03, 0.03)	> 0.99
Concussion	2 (0.01) 2	0	0.01 (-0.01, 0.03)	0.50
Fracture of wrist	1 (< 0.01) 1	0	0.01 (-0.01, 0.02)	> 0.99
Brachial plexus injury	1 (< 0.01) 1	0	0.01 (-0.01, 0.02)	> 0.99
Ankle fracture	1 (< 0.01) 1	0	0.01 (-0.01, 0.02)	> 0.99
Trunk injury	0	1 (< 0.01) 1	-0.01 (-0.02, 0.01)	0.50
Soft tissue injury	2 (0.01) 2	3 (0.02) 3	-0.01 (-0.04, 0.03)	0.69
Alcohol poisoning	1 (< 0.01) 1	0	0.01 (-0.01, 0.02)	> 0.99
Closed globe injury	1 (< 0.01) 1	0	0.01 (-0.01, 0.02)	> 0.99
Facial bones fracture	0	2 (0.01) 2	-0.01 (-0.03, 0.01)	0.25
Ligament sprain	0	1 (< 0.01) 1	-0.01 (-0.02, 0.01)	0.50
Ligament injury	0	1 (< 0.01) 1	-0.01 (-0.02, 0.01)	0.50
Skull fracture	1 (< 0.01) 1	0	0.01 (-0.01, 0.02)	> 0.99
Cervical vertebral fracture	0	1 (< 0.01) 1	-0.01 (-0.02, 0.01)	0.50
Fracture	0	1 (< 0.01) 1	-0.01 (-0.02, 0.01)	0.50
Acetabulum fracture	1 (< 0.01) 1	0	0.01 (-0.01, 0.02)	> 0.99
Hip fracture	1 (< 0.01) 1	2 (0.01) 2	-0.01 (-0.03, 0.02)	0.62

Hepatic enzyme increased	1 (< 0.01) 1	0	0.01 (-0.01, 0.02)	> 0.99
Blood pressure increased	3 (0.02) 3	4 (0.03) 4	-0.01 (-0.04, 0.03)	0.73
Migraine	2 (0.01) 2	1 (< 0.01) 1	0.01 (-0.02, 0.03)	> 0.99
Haemorrhagic stroke	1 (< 0.01) 1	0	0.01 (-0.01, 0.02)	> 0.99
Vestibular migraine	1 (< 0.01) 1	0	0.01 (-0.01, 0.02)	> 0.99
Ageusia	3 (0.02) 3	1 (< 0.01) 1	0.01 (-0.02, 0.04)	0.63
Anosmia	3 (0.02) 3	2 (0.01) 2	0.01 (-0.03, 0.04)	> 0.99
Sciatica	1 (< 0.01) 1	0	0.01 (-0.01, 0.02)	> 0.99
Cerebral infarction	3 (0.02) 3	2 (0.01) 2	0.01 (-0.03, 0.04)	> 0.99
Dizziness	2 (0.01) 2	1 (< 0.01) 1	0.01 (-0.02, 0.03)	> 0.99
Headache	14 (0.09) 17	13 (0.08) 14	0.01 (-0.07, 0.08)	0.85
Paraesthesia	1 (< 0.01) 2	0	0.01 (-0.01, 0.02)	> 0.99
Syncope	1 (< 0.01) 1	1 (< 0.01) 2	0.00 (-0.02, 0.02)	> 0.99
Guillain-Barre syndrome	1 (< 0.01) 1	0	0.01 (-0.01, 0.02)	> 0.99
Transient ischaemic attack	1 (< 0.01) 1	0	0.01 (-0.01, 0.02)	> 0.99
Hypoxic-ischaemic encephalopathy	0	1 (< 0.01) 1	-0.01 (-0.02, 0.01)	0.50
Cerebrovascular accident	3 (0.02) 3	0	0.02 (-0.01, 0.04)	0.25
Cerebrovascular disorder	1 (< 0.01) 1	0	0.01 (-0.01, 0.02)	> 0.99
Lacunar infarction	1 (< 0.01) 1	0	0.01 (-0.01, 0.02)	> 0.99
Subarachnoid haemorrhage	1 (< 0.01) 1	0	0.01 (-0.01, 0.02)	> 0.99
Vascular headache	1 (< 0.01) 1	1 (< 0.01) 1	0.00 (-0.02, 0.02)	> 0.99
Carotid artery stenosis	0	1 (< 0.01) 1	-0.01 (-0.02, 0.01)	0.50
Dysphonia	0	1 (< 0.01) 1	-0.01 (-0.02, 0.01)	0.50

Oropharyngeal pain	5 (0.03) 5	7 (0.05) 7	-0.01 (-0.06, 0.04)	0.56
Dyspnoea	1 (< 0.01) 1	2 (0.01) 2	-0.01 (-0.03, 0.02)	0.62
Haemoptysis	1 (< 0.01) 1	0	0.01 (-0.01, 0.02)	> 0.99
Cough	10 (0.06) 10	6 (0.04) 6	0.03 (-0.03, 0.08)	0.32
Throat irritation	1 (< 0.01) 1	0	0.01 (-0.01, 0.02)	> 0.99
Asthma	3 (0.02) 3	3 (0.02) 3	0.00 (-0.04, 0.04)	> 0.99
Asthmatic crisis	1 (< 0.01) 1	1 (< 0.01) 1	0.00 (-0.02, 0.02)	> 0.99
Status asthmaticus	0	1 (< 0.01) 1	-0.01 (-0.02, 0.01)	0.50
Acute respiratory failure	3 (0.02) 3	1 (< 0.01) 1	0.01 (-0.02, 0.04)	0.63
Chronic obstructive pulmonary disease	4 (0.03) 4	2 (0.01) 2	0.01 (-0.02, 0.05)	0.69
Bronchial obstruction	1 (< 0.01) 1	0	0.01 (-0.01, 0.02)	> 0.99
Pneumothorax	1 (< 0.01) 1	0	0.01 (-0.01, 0.02)	> 0.99
Rhinorrhoea	5 (0.03) 5	6 (0.04) 6	-0.01 (-0.05, 0.04)	0.76
Orthopnoea	1 (< 0.01) 1	0	0.01 (-0.01, 0.02)	> 0.99
Pulmonary embolism	1 (< 0.01) 1	0	0.01 (-0.01, 0.02)	> 0.99
Allergic cough	3 (0.02) 3	0	0.02 (-0.01, 0.04)	0.25
Rhinitis allergic	2 (0.01) 2	4 (0.03) 4	-0.01 (-0.05, 0.02)	0.45
Allergic sinusitis	1 (< 0.01) 1	1 (< 0.01) 1	0.00 (-0.02, 0.02)	> 0.99
Nasal congestion	2 (0.01) 2	1 (< 0.01) 1	0.01 (-0.02, 0.03)	> 0.99
Nasal obstruction	1 (< 0.01) 1	1 (< 0.01) 1	0.00 (-0.02, 0.02)	> 0.99
Abortion incomplete	1 (< 0.01) 1	1 (< 0.01) 1	0.00 (-0.02, 0.02)	> 0.99
Abortion threatened	0	1 (< 0.01) 1	-0.01 (-0.02, 0.01)	0.50
Abortion complete	1 (< 0.01) 1	1 (< 0.01) 1	0.00 (-0.02, 0.02)	> 0.99

Ectopic pregnancy	0	1 (< 0.01) 1	-0.01 (-0.02, 0.01)	0.50
Premature labour	1 (< 0.01) 1	0	0.01 (-0.01, 0.02)	> 0.99
Abortion	0	1 (< 0.01) 1	-0.01 (-0.02, 0.01)	0.50
Abortion missed	0	2 (0.01) 2	-0.01 (-0.03, 0.01)	0.25
Oligohydramnios	0	1 (< 0.01) 1	-0.01 (-0.02, 0.01)	0.50
Foetal death	0	1 (< 0.01) 1	-0.01 (-0.02, 0.01)	0.50
Premature rupture of membranes	0	1 (< 0.01) 1	-0.01 (-0.02, 0.01)	0.50
Abortion spontaneous	1 (< 0.01) 1	2 (0.01) 2	-0.01 (-0.03, 0.02)	0.62
Angina unstable	0	1 (< 0.01) 1	-0.01 (-0.02, 0.01)	0.50
Aortic valve stenosis	0	1 (< 0.01) 1	-0.01 (-0.02, 0.01)	0.50
Cardiac failure congestive	1 (< 0.01) 1	0	0.01 (-0.01, 0.02)	> 0.99
Congestive cardiomyopathy	0	1 (< 0.01) 1	-0.01 (-0.02, 0.01)	0.50
Coronary artery disease	1 (< 0.01) 1	0	0.01 (-0.01, 0.02)	> 0.99
Supraventricular tachycardia	1 (< 0.01) 1	0	0.01 (-0.01, 0.02)	> 0.99
Ventricular extrasystoles	1 (< 0.01) 1	0	0.01 (-0.01, 0.02)	> 0.99
Cardiac failure	1 (< 0.01) 1	2 (0.01) 2	-0.01 (-0.03, 0.02)	0.62
Myocardial infarction	1 (< 0.01) 1	2 (0.01) 2	-0.01 (-0.03, 0.02)	0.62
Myocardial ischaemia	2 (0.01) 3	3 (0.02) 3	-0.01 (-0.04, 0.03)	0.69
Cardio-respiratory arrest	1 (< 0.01) 1	0	0.01 (-0.01, 0.02)	> 0.99
Acute coronary syndrome	2 (0.01) 2	4 (0.03) 4	-0.01 (-0.05, 0.02)	0.45
Cardiac failure acute	0	1 (< 0.01) 1	-0.01 (-0.02, 0.01)	0.50
Acute myocardial infarction	1 (< 0.01) 1	3 (0.02) 3	-0.01 (-0.04, 0.02)	0.37
Atrial fibrillation	0	1 (< 0.01) 1	-0.01 (-0.02, 0.01)	0.50

Hypertensive heart disease	1 (< 0.01) 1	2 (0.01) 2	-0.01 (-0.03, 0.02)	0.62
HIV infection	1 (< 0.01) 1	1 (< 0.01) 1	0.00 (-0.02, 0.02)	> 0.99
Upper respiratory tract infection	32 (0.21) 34	36 (0.23) 40	-0.03 (-0.15, 0.09)	0.63
Lower respiratory tract infection	3 (0.02) 3	5 (0.03) 5	-0.01 (-0.05, 0.03)	0.51
Lower respiratory tract infection bacterial	1 (< 0.01) 1	0	0.01 (-0.01, 0.02)	> 0.99
Scrub typhus	1 (< 0.01) 1	1 (< 0.01) 1	0.00 (-0.02, 0.02)	> 0.99
Otitis media	0	1 (< 0.01) 1	-0.01 (-0.02, 0.01)	0.50
Erysipelas	0	1 (< 0.01) 1	-0.01 (-0.02, 0.01)	0.50
Systemic viral infection	10 (0.06) 11	10 (0.06) 13	0.00 (-0.06, 0.06)	1.00
Respiratory tract infection	5 (0.03) 5	4 (0.03) 4	0.01 (-0.04, 0.05)	> 0.99
Pharyngotonsillitis	10 (0.06) 10	11 (0.07) 11	-0.01 (-0.07, 0.06)	0.83
Pharyngitis	4 (0.03) 4	5 (0.03) 5	-0.01 (-0.05, 0.04)	0.75
Laryngitis	0	1 (< 0.01) 1	-0.01 (-0.02, 0.01)	0.50
Complicated appendicitis	0	1 (< 0.01) 1	-0.01 (-0.02, 0.01)	0.50
Otitis externa	1 (< 0.01) 1	0	0.01 (-0.01, 0.02)	> 0.99
Vulvovaginitis	0	1 (< 0.01) 1	-0.01 (-0.02, 0.01)	0.50
Parasitic gastroenteritis	1 (< 0.01) 1	0	0.01 (-0.01, 0.02)	> 0.99
Infection parasitic	0	3 (0.02) 3	-0.02 (-0.04, 0.01)	0.12
Urinary tract infection	12 (0.08) 14	5 (0.03) 6	0.05 (-0.01, 0.1)	0.09
Localised infection	1 (< 0.01) 1	0	0.01 (-0.01, 0.02)	> 0.99
Bartholin's abscess	2 (0.01) 2	0	0.01 (-0.01, 0.03)	0.50
Otitis media acute	1 (< 0.01) 1	0	0.01 (-0.01, 0.02)	> 0.99

Acute sinusitis	6 (0.04) 6	4 (0.03) 4	0.01 (-0.03, 0.06)	0.75
Septic shock	2 (0.01) 2	2 (0.01) 2	0.00 (-0.03, 0.03)	> 0.99
Septic necrosis	1 (< 0.01) 1	0	0.01 (-0.01, 0.02)	> 0.99
Enterocolitis infectious	1 (< 0.01) 1	0	0.01 (-0.01, 0.02)	> 0.99
Abortion infected	0	1 (< 0.01) 1	-0.01 (-0.02, 0.01)	0.50
Infective myositis	0	1 (< 0.01) 1	-0.01 (-0.02, 0.01)	0.50
Pneumonia	13 (0.08) 13	16 (0.10) 17	-0.02 (-0.1, 0.06)	0.58
Tonsillitis	0	3 (0.02) 3	-0.02 (-0.04, 0.01)	0.12
Bronchitis	6 (0.04) 6	7 (0.05) 7	-0.01 (-0.06, 0.05)	0.78
Sepsis neonatal	0	1 (< 0.01) 1	-0.01 (-0.02, 0.01)	0.50
Postoperative wound infection	0	2 (0.01) 2	-0.01 (-0.03, 0.01)	0.25
Influenza	7 (0.05) 7	12 (0.08) 13	-0.03 (-0.1, 0.03)	0.25
Latent syphilis	0	1 (< 0.01) 1	-0.01 (-0.02, 0.01)	0.50
Tooth abscess	0	2 (0.01) 2	-0.01 (-0.03, 0.01)	0.25
Furuncle	1 (< 0.01) 1	0	0.01 (-0.01, 0.02)	> 0.99
Viral upper respiratory tract infection	10 (0.06) 10	8 (0.05) 11	0.01 (-0.05, 0.07)	0.64
Viral pharyngitis	1 (< 0.01) 1	2 (0.01) 2	-0.01 (-0.03, 0.02)	0.62
Gastroenteritis viral	4 (0.03) 4	3 (0.02) 3	0.01 (-0.03, 0.04)	> 0.99
Viral infection	5 (0.03) 5	3 (0.02) 3	0.01 (-0.03, 0.05)	0.73
Carbuncle	1 (< 0.01) 1	1 (< 0.01) 1	0.00 (-0.02, 0.02)	> 0.99
Dengue fever	9 (0.06) 9	11 (0.07) 11	-0.01 (-0.08, 0.05)	0.65
Skin infection	0	1 (< 0.01) 1	-0.01 (-0.02, 0.01)	0.50
Bacterial infection	0	1 (< 0.01) 1	-0.01 (-0.02, 0.01)	0.50

Pneumonia bacterial	1 (< 0.01) 1	1 (< 0.01) 2	0.00 (-0.02, 0.02)	> 0.99
Gastroenteritis bacterial	0	1 (< 0.01) 1	-0.01 (-0.02, 0.01)	0.50
Osteomyelitis bacterial	1 (< 0.01) 1	0	0.01 (-0.01, 0.02)	> 0.99
Meningitis tuberculous	1 (< 0.01) 1	0	0.01 (-0.01, 0.02)	> 0.99
Tuberculosis	2 (0.01) 2	0	0.01 (-0.01, 0.03)	0.50
Conjunctivitis	2 (0.01) 2	1 (< 0.01) 1	0.01 (-0.02, 0.03)	> 0.99
Liver abscess	1 (< 0.01) 1	0	0.01 (-0.01, 0.02)	> 0.99
Abscess limb	1 (< 0.01) 1	0	0.01 (-0.01, 0.02)	> 0.99
Pulmonary tuberculosis	1 (< 0.01) 1	3 (0.02) 3	-0.01 (-0.04, 0.02)	0.37
Gastroenteritis	5 (0.03) 5	4 (0.03) 4	0.01 (-0.04, 0.05)	> 0.99
Sepsis	2 (0.01) 2	2 (0.01) 2	0.00 (-0.03, 0.03)	> 0.99
Cystitis	1 (< 0.01) 1	1 (< 0.01) 1	0.00 (-0.02, 0.02)	> 0.99
Cellulitis	4 (0.03) 4	4 (0.03) 4	0.00 (-0.04, 0.04)	> 0.99
Gastric ulcer helicobacter	0	1 (< 0.01) 1	-0.01 (-0.02, 0.01)	0.50
Helicobacter infection	0	1 (< 0.01) 1	-0.01 (-0.02, 0.01)	0.50
Soft tissue infection	0	1 (< 0.01) 1	-0.01 (-0.02, 0.01)	0.50
Leptospirosis	0	1 (< 0.01) 1	-0.01 (-0.02, 0.01)	0.50
Plasmodium vivax infection	0	1 (< 0.01) 1	-0.01 (-0.02, 0.01)	0.50
Appendicitis	3 (0.02) 3	2 (0.01) 2	0.01 (-0.03, 0.04)	> 0.99
Osteomyelitis	0	1 (< 0.01) 1	-0.01 (-0.02, 0.01)	0.50
Nasopharyngitis	61 (0.39) 66	50 (0.32) 57	0.07 (-0.08, 0.22)	0.30
Rhinitis	1 (< 0.01) 1	0	0.01 (-0.01, 0.02)	> 0.99
Sinusitis	0	2 (0.01) 2	-0.01 (-0.03, 0.01)	0.25

Gingivitis	1 (< 0.01) 1	0	0.01 (-0.01, 0.02)	> 0.99
Breast dysplasia	0	1 (< 0.01) 1	-0.01 (-0.02, 0.01)	0.50
Breast cyst	0	1 (< 0.01) 1	-0.01 (-0.02, 0.01)	0.50
Ovarian cyst	1 (< 0.01) 1	0	0.01 (-0.01, 0.02)	> 0.99
Endometrial hyperplasia	0	1 (< 0.01) 1	-0.01 (-0.02, 0.01)	0.50
Adenomyosis	1 (< 0.01) 1	0	0.01 (-0.01, 0.02)	> 0.99
Abnormal uterine bleeding	3 (0.02) 3	1 (< 0.01) 1	0.01 (-0.02, 0.04)	0.63
Benign prostatic hyperplasia	1 (< 0.01) 1	0	0.01 (-0.01, 0.02)	> 0.99
Papule	0	1 (< 0.01) 1	-0.01 (-0.02, 0.01)	0.50
Urticaria papular	1 (< 0.01) 1	0	0.01 (-0.01, 0.02)	> 0.99
Toxic epidermal necrolysis	1 (< 0.01) 1	0	0.01 (-0.01, 0.02)	> 0.99
Stevens-Johnson syndrome	1 (< 0.01) 2	0	0.01 (-0.01, 0.02)	> 0.99
Urticaria chronic	1 (< 0.01) 1	0	0.01 (-0.01, 0.02)	> 0.99
Dermatitis contact	1 (< 0.01) 1	1 (< 0.01) 1	0.00 (-0.02, 0.02)	> 0.99
Dermatitis atopic	1 (< 0.01) 1	0	0.01 (-0.01, 0.02)	> 0.99
Pruritus	0	1 (< 0.01) 1	-0.01 (-0.02, 0.01)	0.50
Dermatitis	1 (< 0.01) 1	0	0.01 (-0.01, 0.02)	> 0.99
Skin ulcer	1 (< 0.01) 1	1 (< 0.01) 1	0.00 (-0.02, 0.02)	> 0.99
Skin lesion	1 (< 0.01) 1	0	0.01 (-0.01, 0.02)	> 0.99
Neurodermatitis	1 (< 0.01) 1	0	0.01 (-0.01, 0.02)	> 0.99
Diabetic foot	3 (0.02) 3	2 (0.01) 2	0.01 (-0.03, 0.04)	> 0.99
Erythema nodosum	1 (< 0.01) 1	0	0.01 (-0.01, 0.02)	> 0.99
Cellulite	1 (< 0.01) 1	1 (< 0.01) 1	0.00 (-0.02, 0.02)	> 0.99

Alopecia	1 (< 0.01) 1	0	0.01 (-0.01, 0.02)	> 0.99
Urticaria	3 (0.02) 3	2 (0.01) 2	0.01 (-0.03, 0.04)	> 0.99
Yellow skin	0	1 (< 0.01) 1	-0.01 (-0.02, 0.01)	0.50
Eyelid ptosis	1 (< 0.01) 1	0	0.01 (-0.01, 0.02)	> 0.99
Blindness	0	1 (< 0.01) 1	-0.01 (-0.02, 0.01)	0.50
Cataract	1 (< 0.01) 1	0	0.01 (-0.01, 0.02)	> 0.99
Diabetic blindness	1 (< 0.01) 1	0	0.01 (-0.01, 0.02)	> 0.99
Macular hole	0	1 (< 0.01) 1	-0.01 (-0.02, 0.01)	0.50
Bipolar disorder	0	1 (< 0.01) 1	-0.01 (-0.02, 0.01)	0.50
Insomnia	1 (< 0.01) 1	0	0.01 (-0.01, 0.02)	> 0.99
Completed suicide	1 (< 0.01) 1	0	0.01 (-0.01, 0.02)	> 0.99
Premature ejaculation	1 (< 0.01) 1	0	0.01 (-0.01, 0.02)	> 0.99
Anxiety	1 (< 0.01) 1	0	0.01 (-0.01, 0.02)	> 0.99
Anxiety disorder	3 (0.02) 3	0	0.02 (-0.01, 0.04)	0.25
Substance abuse	2 (0.01) 2	0	0.01 (-0.01, 0.03)	0.50
Sleep disorder	1 (< 0.01) 1	0	0.01 (-0.01, 0.02)	> 0.99
Substance-induced psychotic disorder	1 (< 0.01) 1	0	0.01 (-0.01, 0.02)	> 0.99
Behaviour disorder	1 (< 0.01) 1	0	0.01 (-0.01, 0.02)	> 0.99
Vertigo positional	0	1 (< 0.01) 2	-0.01 (-0.02, 0.01)	0.50
Vestibular disorder	3 (0.02) 4	2 (0.01) 2	0.01 (-0.03, 0.04)	> 0.99
Vertigo	0	2 (0.01) 2	-0.01 (-0.03, 0.01)	0.25
Ear pain	1 (< 0.01) 1	1 (< 0.01) 1	0.00 (-0.02, 0.02)	> 0.99
Cholecystitis acute	1 (< 0.01) 1	0	0.01 (-0.01, 0.02)	> 0.99

Hepatic cirrhosis	0	2 (0.01) 2	-0.01 (-0.03, 0.01)	0.25
Hepatic steatosis	1 (< 0.01) 1	0	0.01 (-0.01, 0.02)	> 0.99
Cholelithiasis	1 (< 0.01) 1	1 (< 0.01) 1	0.00 (-0.02, 0.02)	> 0.99
Cholangitis	0	1 (< 0.01) 1	-0.01 (-0.02, 0.01)	0.50
Cirrhosis alcoholic	0	1 (< 0.01) 1	-0.01 (-0.02, 0.01)	0.50
Nocturia	1 (< 0.01) 1	0	0.01 (-0.01, 0.02)	> 0.99
Urinary tract obstruction	0	1 (< 0.01) 1	-0.01 (-0.02, 0.01)	0.50
Acute kidney injury	0	2 (0.01) 2	-0.01 (-0.03, 0.01)	0.25
Chronic kidney disease	1 (< 0.01) 1	1 (< 0.01) 1	0.00 (-0.02, 0.02)	> 0.99
Azotaemia	1 (< 0.01) 1	0	0.01 (-0.01, 0.02)	> 0.99
Diabetic end stage renal disease	0	1 (< 0.01) 1	-0.01 (-0.02, 0.01)	0.50
End stage renal disease	0	1 (< 0.01) 1	-0.01 (-0.02, 0.01)	0.50
Nephrolithiasis	0	3 (0.02) 3	-0.02 (-0.04, 0.01)	0.12
Renal colic	0	1 (< 0.01) 1	-0.01 (-0.02, 0.01)	0.50
Haematuria	1 (< 0.01) 1	0	0.01 (-0.01, 0.02)	> 0.99
Ureterolithiasis	0	2 (0.01) 2	-0.01 (-0.03, 0.01)	0.25
Upper gastrointestinal haemorrhage	1 (< 0.01) 1	1 (< 0.01) 1	0.00 (-0.02, 0.02)	> 0.99
Abdominal pain upper	1 (< 0.01) 1	1 (< 0.01) 1	0.00 (-0.02, 0.02)	> 0.99
Dysphagia	0	2 (0.01) 2	-0.01 (-0.03, 0.01)	0.25
Vomiting	3 (0.02) 4	0	0.02 (-0.01, 0.04)	0.25
Pancreatitis acute	1 (< 0.01) 1	1 (< 0.01) 1	0.00 (-0.02, 0.02)	> 0.99
Acute abdomen	0	1 (< 0.01) 1	-0.01 (-0.02, 0.01)	0.50
Nausea	6 (0.04) 6	1 (< 0.01) 1	0.03 (-0.01, 0.07)	0.13

Peptic ulcer perforation	0	1 (< 0.01) 1	-0.01 (-0.02, 0.01)	0.50
Toothache	1 (< 0.01) 1	0	0.01 (-0.01, 0.02)	> 0.99
Haemorrhoids	1 (< 0.01) 1	1 (< 0.01) 1	0.00 (-0.02, 0.02)	> 0.99
Gastritis erosive	0	1 (< 0.01) 1	-0.01 (-0.02, 0.01)	0.50
Inguinal hernia strangulated	1 (< 0.01) 1	0	0.01 (-0.01, 0.02)	> 0.99
Anal stenosis	0	1 (< 0.01) 1	-0.01 (-0.02, 0.01)	0.50
Intestinal ischaemia	0	1 (< 0.01) 1	-0.01 (-0.02, 0.01)	0.50
Gastric haemorrhage	1 (< 0.01) 1	0	0.01 (-0.01, 0.02)	> 0.99
Gastritis	4 (0.03) 4	2 (0.01) 2	0.01 (-0.02, 0.05)	0.69
Gastrointestinal haemorrhage	1 (< 0.01) 1	0	0.01 (-0.01, 0.02)	> 0.99
Gastrointestinal disorder	1 (< 0.01) 1	0	0.01 (-0.01, 0.02)	> 0.99
Hyperchlorhydria	1 (< 0.01) 1	0	0.01 (-0.01, 0.02)	> 0.99
Gastrooesophageal reflux disease	8 (0.05) 8	4 (0.03) 4	0.03 (-0.02, 0.08)	0.25
Umbilical hernia	0	2 (0.01) 2	-0.01 (-0.03, 0.01)	0.25
Ascites	0	1 (< 0.01) 1	-0.01 (-0.02, 0.01)	0.50
Diarrhoea	6 (0.04) 6	4 (0.03) 4	0.01 (-0.03, 0.06)	0.75
Abdominal pain	0	3 (0.02) 3	-0.02 (-0.04, 0.01)	0.12
Inguinal hernia	2 (0.01) 2	0	0.01 (-0.01, 0.03)	0.50
Dental caries	0	1 (< 0.01) 1	-0.01 (-0.02, 0.01)	0.50
Hypopharyngeal cancer	0	1 (< 0.01) 1	-0.01 (-0.02, 0.01)	0.50
Breast cancer	0	1 (< 0.01) 1	-0.01 (-0.02, 0.01)	0.50
Glioblastoma multiforme	0	1 (< 0.01) 1	-0.01 (-0.02, 0.01)	0.50
Endometrial adenocarcinoma	1 (< 0.01) 1	0	0.01 (-0.01, 0.02)	> 0.99

Uterine leiomyoma	1 (< 0.01) 1	1 (< 0.01) 1	0.00 (-0.02, 0.02)	> 0.99
Cervix carcinoma stage II	1 (< 0.01) 1	0	0.01 (-0.01, 0.02)	> 0.99
Cervix carcinoma stage III	1 (< 0.01) 1	0	0.01 (-0.01, 0.02)	> 0.99
Bronchial carcinoma	0	1 (< 0.01) 1	-0.01 (-0.02, 0.01)	0.50
Tracheal cancer	1 (< 0.01) 1	0	0.01 (-0.01, 0.02)	> 0.99
Testis cancer	0	1 (< 0.01) 1	-0.01 (-0.02, 0.01)	0.50
Testicular neoplasm	0	1 (< 0.01) 1	-0.01 (-0.02, 0.01)	0.50
Hepatocellular carcinoma	1 (< 0.01) 2	1 (< 0.01) 1	0.00 (-0.02, 0.02)	> 0.99
Lung neoplasm malignant	1 (< 0.01) 1	0	0.01 (-0.01, 0.02)	> 0.99
Metastases to lung	1 (< 0.01) 1	0	0.01 (-0.01, 0.02)	> 0.99
Cholangiocarcinoma	1 (< 0.01) 1	0	0.01 (-0.01, 0.02)	> 0.99
Lipoma	1 (< 0.01) 1	0	0.01 (-0.01, 0.02)	> 0.99
Abdominal neoplasm	0	1 (< 0.01) 1	-0.01 (-0.02, 0.01)	0.50
Benign neoplasm of skin	0	1 (< 0.01) 1	-0.01 (-0.02, 0.01)	0.50
Metastatic neoplasm	0	1 (< 0.01) 1	-0.01 (-0.02, 0.01)	0.50
Metastatic gastric cancer	1 (< 0.01) 1	0	0.01 (-0.01, 0.02)	> 0.99
Squamous cell carcinoma	1 (< 0.01) 1	0	0.01 (-0.01, 0.02)	> 0.99
Bicytopenia	1 (< 0.01) 1	0	0.01 (-0.01, 0.02)	> 0.99
Blood loss anaemia	1 (< 0.01) 1	2 (0.01) 2	-0.01 (-0.03, 0.02)	0.62
Iron deficiency anaemia	0	1 (< 0.01) 1	-0.01 (-0.02, 0.01)	0.50
Anaemia	2 (0.01) 2	0	0.01 (-0.01, 0.03)	0.50
Superior vena cava syndrome	0	1 (< 0.01) 1	-0.01 (-0.02, 0.01)	0.50
Aortic aneurysm	1 (< 0.01) 1	0	0.01 (-0.01, 0.02)	> 0.99

Hypotension	1 (< 0.01) 1	0	0.01 (-0.01, 0.02)	> 0.99
Hypovolaemic shock	0	1 (< 0.01) 1	-0.01 (-0.02, 0.01)	0.50
Hot flush	1 (< 0.01) 1	0	0.01 (-0.01, 0.02)	> 0.99
Extremity necrosis	0	1 (< 0.01) 1	-0.01 (-0.02, 0.01)	0.50
Intermittent claudication	1 (< 0.01) 1	0	0.01 (-0.01, 0.02)	> 0.99
Hypertension	21 (0.14) 21	19 (0.12) 19	0.01 (-0.08, 0.1)	0.75
Hypertensive emergency	1 (< 0.01) 1	1 (< 0.01) 1	0.00 (-0.02, 0.02)	> 0.99

The analysis was based on the participants in the safety analysis set, grouped according to actual intervention. Participants who received at least one dose of Pneucolin® were included in the vaccine group. Data of incidence are shown as n (%) m: n, number of participants; %, proportion of participants; m, number of events.

Table S11. Adverse events of special interest in safety analysis set throughout the observation period.

Preferred Term	Vaccine group (n=15500) n(%) m	Placebo group (n=15490) n(%) m	Rate difference (95% CI)	*P* value
Food allergy	1 (< 0.01) 1	0	0.01 (-0.01, 0.02)	> 0.99
Ageusia	50 (0.32) 50	55 (0.36) 57	-0.03 (-0.18, 0.12)	0.62
Anosmia	26 (0.17) 26	23 (0.15) 23	0.02 (-0.08, 0.12)	0.67
Cerebral infarction	2 (0.01) 2	0	0.01 (-0.01, 0.03)	0.50
Guillain-Barre syndrome	1 (< 0.01) 1	0	0.01 (-0.01, 0.02)	> 0.99
Cerebrovascular accident	1 (< 0.01) 1	0	0.01 (-0.01, 0.02)	> 0.99
Lacunar infarction	1 (< 0.01) 1	0	0.01 (-0.01, 0.02)	> 0.99
Acute respiratory failure	2 (0.01) 2	0	0.01 (-0.01, 0.03)	0.50
Supraventricular tachycardia	1 (< 0.01) 1	0	0.01 (-0.01, 0.02)	> 0.99
Cardiac failure	0	2 (0.01) 2	-0.01 (-0.03, 0.01)	0.25
Arrhythmia	2 (0.01) 2	1 (< 0.01) 1	0.01 (-0.02, 0.03)	> 0.99
Myocardial ischaemia	1 (< 0.01) 1	2 (0.01) 2	-0.01 (-0.03, 0.02)	0.62
Cardiovascular disorder	0	1 (< 0.01) 1	-0.01 (-0.02, 0.01)	0.50
Acute coronary syndrome	1 (< 0.01) 1	2 (0.01) 2	-0.01 (-0.03, 0.02)	0.62
Acute myocardial infarction	1 (< 0.01) 1	2 (0.01) 2	-0.01 (-0.03, 0.02)	0.62
Pneumonia	1 (< 0.01) 1	0	0.01 (-0.01, 0.02)	> 0.99
Meningitis tuberculous	1 (< 0.01) 1	0	0.01 (-0.01, 0.02)	> 0.99
Cutaneous vasculitis	0	1 (< 0.01) 1	-0.01 (-0.02, 0.01)	0.50
Acute kidney injury	0	1 (< 0.01) 1	-0.01 (-0.02, 0.01)	0.50
Pancreatitis acute	1 (< 0.01) 1	1 (< 0.01) 1	0.00 (-0.02, 0.02)	> 0.99

The analysis was based on the participants in the safety analysis set, grouped according to actual intervention. Participants who received at least one dose of Pneucolin® were included in the vaccine group. Data of incidence are shown as n (%)m: n, number of participants; %, proportion of participants; m, number of events. The adverse events of special interest specified in the protocol are derived from Brighton Collaboration's Safety Platform for Emergency Vaccines (SPEAC).

Table S12. List* of Adverse events of special interest in safety analysis set throughout the observation period.

The listings of AESIs related to COVID-19	Vaccine group (N=15500) n (%) m	Placebo group (N=15490) n (%) m
Acute respiratory distress syndrome	3 (0.02) 3	0
Multisystem inflammatory syndrome	0	0
Acute cardiovascular injury	6 (0.04) 6	10 (0.06) 10
Coagulation disorder	4 (0.03) 4	0
Anosmia, ageusia	61 (0.39) 76	65 (0.42) 80
Chilblain – like lesions	0	0
Erythema multiforme	0	0
Single Organ Cutaneous Vasculitis	0	1 (<0.01) 1
Acute kidney injury	0	1 (<0.01) 1
Acute liver injury	0	0
Acute pancreatitis	1 (<0.01) 1	1 (<0.01) 1
Rhabdomyolysis	0	0
Subacute thyroiditis	0	0
Anaphylaxis	1 (<0.01) 1	0
Thrombocytopenia	0	0
Generalized convulsion	0	0
Acute disseminated encephalomyelitis	0	0
Guillain Barré Syndrome	1 (<0.01) 1	0
Acute aseptic arthritis	0	0
Aseptic meningitis	1 (<0.01) 1	0
Encephalitis / Encephalomyelitis	0	0
Idiopathic Peripheral Facial Nerve Palsy	0	0
Vaccine associated enhanced disease	0	0

*This listing is originated from Brighton Collaboration's Safety Platform for Emergency Vaccines (SPEAC).

Table S13. Serious adverse events in safety analysis set throughout the observation period.

Preferred Term	Vaccine group (n=15500) n(%) m	Placebo group (n=15490) n(%) m	Rate difference (95% CI)	P value
Type 2 diabetes mellitus	4 (0.03) 4	1 (< 0.01) 1	0.02 (-0.01, 0.05)	0.38
Hypoglycaemia	0	1 (< 0.01) 1	-0.01 (-0.02, 0.01)	0.50
Hypokalaemia	0	1 (< 0.01) 1	-0.01 (-0.02, 0.01)	0.50
Electrolyte imbalance	0	1 (< 0.01) 1	-0.01 (-0.02, 0.01)	0.50
Gout	0	1 (< 0.01) 1	-0.01 (-0.02, 0.01)	0.50
Diabetes mellitus	0	1 (< 0.01) 1	-0.01 (-0.02, 0.01)	0.50
Diabetic metabolic decompensation	0	2 (0.01) 2	-0.01 (-0.03, 0.01)	0.25
Diabetes mellitus inadequate control	0	1 (< 0.01) 1	-0.01 (-0.02, 0.01)	0.50
Dehydration	0	1 (< 0.01) 1	-0.01 (-0.02, 0.01)	0.50
Dyslipidaemia	1 (< 0.01) 1	0	0.01 (-0.01, 0.02)	> 0.99
Food allergy	1 (< 0.01) 1	0	0.01 (-0.01, 0.02)	> 0.99
Multiple organ dysfunction syndrome	0	1 (< 0.01) 1	-0.01 (-0.02, 0.01)	0.50
Sudden cardiac death	1 (< 0.01) 1	0	0.01 (-0.01, 0.02)	> 0.99
Accidental death	1 (< 0.01) 1	0	0.01 (-0.01, 0.02)	> 0.99
Death	4 (0.03) 4	4 (0.03) 4	0.00 (-0.04, 0.04)	> 0.99
Inflammation	0	1 (< 0.01) 1	-0.01 (-0.02, 0.01)	0.50
Sudden death	3 (0.02) 3	1 (< 0.01) 1	0.01 (-0.02, 0.04)	0.63
Fatigue	0	1 (< 0.01) 1	-0.01 (-0.02, 0.01)	0.50
Hanging	0	1 (< 0.01) 1	-0.01 (-0.02, 0.01)	0.50

Preferred Term	Vaccine group (n=15500) n(%) m	Placebo group (n=15490) n(%) m	Rate difference (95% CI)	P value
Adrenal insufficiency	1 (< 0.01) 1	0	0.01 (-0.01, 0.02)	> 0.99
Trisomy 21	1 (< 0.01) 1	0	0.01 (-0.01, 0.02)	> 0.99
Tumour excision	1 (< 0.01) 1	0	0.01 (-0.01, 0.02)	> 0.99
Joint effusion	0	1 (< 0.01) 1	-0.01 (-0.02, 0.01)	0.50
Arthritis	0	1 (< 0.01) 1	-0.01 (-0.02, 0.01)	0.50
Intervertebral disc disorder	0	1 (< 0.01) 1	-0.01 (-0.02, 0.01)	0.50
Intervertebral disc displacement	0	2 (0.01) 2	-0.01 (-0.03, 0.01)	0.25
Intervertebral disc protrusion	0	1 (< 0.01) 1	-0.01 (-0.02, 0.01)	0.50
Myopathy	1 (< 0.01) 1	0	0.01 (-0.01, 0.02)	> 0.99
Back pain	1 (< 0.01) 1	0	0.01 (-0.01, 0.02)	> 0.99
Spinal osteoarthritis	1 (< 0.01) 1	1 (< 0.01) 1	0.00 (-0.02, 0.02)	> 0.99
Human bite	1 (< 0.01) 1	0	0.01 (-0.01, 0.02)	> 0.99
Wound	2 (0.01) 2	2 (0.01) 2	0.00 (-0.03, 0.03)	> 0.99
Joint injury	1 (< 0.01) 1	1 (< 0.01) 1	0.00 (-0.02, 0.02)	> 0.99
Joint dislocation	1 (< 0.01) 1	0	0.01 (-0.01, 0.02)	> 0.99
Traumatic amputation	1 (< 0.01) 1	0	0.01 (-0.01, 0.02)	> 0.99
Multiple injuries	3 (0.02) 3	2 (0.01) 2	0.01 (-0.03, 0.04)	> 0.99
Multiple fractures	1 (< 0.01) 1	0	0.01 (-0.01, 0.02)	> 0.99
Head injury	1 (< 0.01) 1	2 (0.01) 2	-0.01 (-0.03, 0.02)	0.62
Ulna fracture	1 (< 0.01) 1	0	0.01 (-0.01, 0.02)	> 0.99
Stab wound	1 (< 0.01) 1	0	0.01 (-0.01, 0.02)	> 0.99

Preferred Term	Vaccine group (n=15500) n(%) m	Placebo group (n=15490) n(%) m	Rate difference (95% CI)	P value
Hand fracture	1 (< 0.01) 1	0	0.01 (-0.01, 0.02)	> 0.99
Gun shot wound	2 (0.01) 2	3 (0.02) 3	-0.01 (-0.04, 0.03)	0.69
Radius fracture	1 (< 0.01) 1	0	0.01 (-0.01, 0.02)	> 0.99
Spinal compression fracture	0	1 (< 0.01) 1	-0.01 (-0.02, 0.01)	0.50
Thermal burn	0	1 (< 0.01) 1	-0.01 (-0.02, 0.01)	0.50
Overdose	1 (< 0.01) 1	0	0.01 (-0.01, 0.02)	> 0.99
Skin laceration	2 (0.01) 2	0	0.01 (-0.01, 0.03)	0.50
Skin abrasion	1 (< 0.01) 1	0	0.01 (-0.01, 0.02)	> 0.99
Eye contusion	1 (< 0.01) 1	0	0.01 (-0.01, 0.02)	> 0.99
Epidural haemorrhage	0	1 (< 0.01) 1	-0.01 (-0.02, 0.01)	0.50
Tendon injury	1 (< 0.01) 1	1 (< 0.01) 1	0.00 (-0.02, 0.02)	> 0.99
Tendon rupture	0	1 (< 0.01) 1	-0.01 (-0.02, 0.01)	0.50
Femur fracture	1 (< 0.01) 1	0	0.01 (-0.01, 0.02)	> 0.99
Limb crushing injury	0	1 (< 0.01) 1	-0.01 (-0.02, 0.01)	0.50
Tibia fracture	2 (0.01) 2	1 (< 0.01) 1	0.01 (-0.02, 0.03)	> 0.99
Concussion	2 (0.01) 2	0	0.01 (-0.01, 0.03)	0.50
Fracture of wrist	1 (< 0.01) 1	0	0.01 (-0.01, 0.02)	> 0.99
Ankle fracture	1 (< 0.01) 1	0	0.01 (-0.01, 0.02)	> 0.99
Soft tissue injury	2 (0.01) 2	2 (0.01) 2	0.00 (-0.03, 0.03)	> 0.99
Road traffic accident	1 (< 0.01) 1	0	0.01 (-0.01, 0.02)	> 0.99
Alcohol poisoning	1 (< 0.01) 1	0	0.01 (-0.01, 0.02)	> 0.99

Preferred Term	Vaccine group (n=15500) n(%) m	Placebo group (n=15490) n(%) m	Rate difference (95% CI)	P value
Closed globe injury	1 (< 0.01) 1	0	0.01 (-0.01, 0.02)	> 0.99
Facial bones fracture	0	2 (0.01) 2	-0.01 (-0.03, 0.01)	0.25
Ligament injury	0	1 (< 0.01) 1	-0.01 (-0.02, 0.01)	0.50
Skull fracture	1 (< 0.01) 1	0	0.01 (-0.01, 0.02)	> 0.99
Cervical vertebral fracture	0	1 (< 0.01) 1	-0.01 (-0.02, 0.01)	0.50
Acetabulum fracture	1 (< 0.01) 1	0	0.01 (-0.01, 0.02)	> 0.99
Hip fracture	0	2 (0.01) 2	-0.01 (-0.03, 0.01)	0.25
Weight decreased	1 (< 0.01) 1	0	0.01 (-0.01, 0.02)	> 0.99
Haemorrhagic stroke	1 (< 0.01) 1	0	0.01 (-0.01, 0.02)	> 0.99
Vestibular migraine	1 (< 0.01) 1	0	0.01 (-0.01, 0.02)	> 0.99
Sciatica	1 (< 0.01) 1	0	0.01 (-0.01, 0.02)	> 0.99
Cerebral infarction	4 (0.03) 4	2 (0.01) 2	0.01 (-0.02, 0.05)	0.69
Syncope	0	1 (< 0.01) 1	-0.01 (-0.02, 0.01)	0.50
Guillain-Barre syndrome	1 (< 0.01) 1	0	0.01 (-0.01, 0.02)	> 0.99
Hypoxic-ischaemic encephalopathy	0	1 (< 0.01) 1	-0.01 (-0.02, 0.01)	0.50
Cerebrovascular accident	5 (0.03) 5	1 (< 0.01) 1	0.03 (-0.01, 0.06)	0.22
Cerebrovascular disorder	1 (< 0.01) 1	0	0.01 (-0.01, 0.02)	> 0.99
Lacunar infarction	1 (< 0.01) 1	0	0.01 (-0.01, 0.02)	> 0.99
Subarachnoid haemorrhage	1 (< 0.01) 1	0	0.01 (-0.01, 0.02)	> 0.99
Dyspnoea	0	2 (0.01) 2	-0.01 (-0.03, 0.01)	0.25
Asthma	2 (0.01) 2	1 (< 0.01) 1	0.01 (-0.02, 0.03)	> 0.99

Preferred Term	Vaccine group (n=15500) n(%) m	Placebo group (n=15490) n(%) m	Rate difference (95% CI)	P value
Status asthmaticus	0	1 (< 0.01) 1	-0.01 (-0.02, 0.01)	0.50
Acute respiratory failure	3 (0.02) 3	2 (0.01) 2	0.01 (-0.03, 0.04)	> 0.99
Chronic obstructive pulmonary disease	2 (0.01) 2	1 (< 0.01) 1	0.01 (-0.02, 0.03)	> 0.99
Pneumothorax	1 (< 0.01) 1	0	0.01 (-0.01, 0.02)	> 0.99
Asphyxia	0	2 (0.01) 2	-0.01 (-0.03, 0.01)	0.25
Pulmonary embolism	1 (< 0.01) 1	0	0.01 (-0.01, 0.02)	> 0.99
Abortion incomplete	1 (< 0.01) 1	1 (< 0.01) 1	0.00 (-0.02, 0.02)	> 0.99
Abortion threatened	0	1 (< 0.01) 1	-0.01 (-0.02, 0.01)	0.50
Abortion complete	1 (< 0.01) 1	1 (< 0.01) 1	0.00 (-0.02, 0.02)	> 0.99
Ectopic pregnancy	0	1 (< 0.01) 1	-0.01 (-0.02, 0.01)	0.50
Premature labour	1 (< 0.01) 1	0	0.01 (-0.01, 0.02)	> 0.99
Abortion	0	1 (< 0.01) 1	-0.01 (-0.02, 0.01)	0.50
Abortion missed	0	2 (0.01) 2	-0.01 (-0.03, 0.01)	0.25
Oligohydramnios	0	1 (< 0.01) 1	-0.01 (-0.02, 0.01)	0.50
Foetal death	0	1 (< 0.01) 1	-0.01 (-0.02, 0.01)	0.50
Premature rupture of membranes	0	1 (< 0.01) 1	-0.01 (-0.02, 0.01)	0.50
Abortion spontaneous	2 (0.01) 2	3 (0.02) 3	-0.01 (-0.04, 0.03)	0.69
Angina unstable	0	1 (< 0.01) 1	-0.01 (-0.02, 0.01)	0.50
Aortic valve stenosis	0	1 (< 0.01) 1	-0.01 (-0.02, 0.01)	0.50
Cardiac failure congestive	1 (< 0.01) 1	0	0.01 (-0.01, 0.02)	> 0.99
Congestive cardiomyopathy	0	1 (< 0.01) 1	-0.01 (-0.02, 0.01)	0.50

Preferred Term	Vaccine group (n=15500) n(%) m	Placebo group (n=15490) n(%) m	Rate difference (95% CI)	P value
Coronary artery disease	1 (< 0.01) 1	0	0.01 (-0.01, 0.02)	> 0.99
Supraventricular tachycardia	1 (< 0.01) 1	0	0.01 (-0.01, 0.02)	> 0.99
Ventricular extrasystoles	1 (< 0.01) 1	0	0.01 (-0.01, 0.02)	> 0.99
Cardiac failure	1 (< 0.01) 2	3 (0.02) 3	-0.01 (-0.04, 0.02)	0.37
Arrhythmia	1 (< 0.01) 1	1 (< 0.01) 1	0.00 (-0.02, 0.02)	> 0.99
Myocardial infarction	1 (< 0.01) 1	3 (0.02) 3	-0.01 (-0.04, 0.02)	0.37
Myocardial ischaemia	1 (< 0.01) 1	1 (< 0.01) 1	0.00 (-0.02, 0.02)	> 0.99
Cardiac arrest	1 (< 0.01) 1	0	0.01 (-0.01, 0.02)	> 0.99
Cardiovascular disorder	0	1 (< 0.01) 1	-0.01 (-0.02, 0.01)	0.50
Acute coronary syndrome	2 (0.01) 2	5 (0.03) 5	-0.02 (-0.06, 0.02)	0.29
Cardiac failure acute	0	1 (< 0.01) 1	-0.01 (-0.02, 0.01)	0.50
Acute myocardial infarction	1 (< 0.01) 1	8 (0.05) 8	-0.05 (-0.09, 0.00)	0.02
Atrial fibrillation	0	1 (< 0.01) 1	-0.01 (-0.02, 0.01)	0.50
Hypertensive heart disease	1 (< 0.01) 1	1 (< 0.01) 1	0.00 (-0.02, 0.02)	> 0.99
Lower respiratory tract infection	1 (< 0.01) 1	1 (< 0.01) 1	0.00 (-0.02, 0.02)	> 0.99
Scrub typhus	1 (< 0.01) 1	1 (< 0.01) 1	0.00 (-0.02, 0.02)	> 0.99
Central nervous system infection	1 (< 0.01) 1	0	0.01 (-0.01, 0.02)	> 0.99
Erysipelas	0	1 (< 0.01) 1	-0.01 (-0.02, 0.01)	0.50
Respiratory tract infection	1 (< 0.01) 1	0	0.01 (-0.01, 0.02)	> 0.99
Pharyngitis	1 (< 0.01) 1	2 (0.01) 2	-0.01 (-0.03, 0.02)	0.62
Complicated appendicitis	0	1 (< 0.01) 1	-0.01 (-0.02, 0.01)	0.50

Preferred Term	Vaccine group (n=15500) n(%) m	Placebo group (n=15490) n(%) m	Rate difference (95% CI)	P value
Parasitic gastroenteritis	1 (< 0.01) 1	0	0.01 (-0.01, 0.02)	> 0.99
Urinary tract infection	6 (0.04) 8	2 (0.01) 2	0.03 (-0.02, 0.07)	0.29
Septic shock	3 (0.02) 3	2 (0.01) 2	0.01 (-0.03, 0.04)	> 0.99
Septic necrosis	1 (< 0.01) 1	0	0.01 (-0.01, 0.02)	> 0.99
Abortion infected	0	1 (< 0.01) 1	-0.01 (-0.02, 0.01)	0.50
Pneumonia	10 (0.06) 10	10 (0.06) 10	0.00 (-0.06, 0.06)	1.00
Tonsillitis	0	1 (< 0.01) 1	-0.01 (-0.02, 0.01)	0.50
Sepsis neonatal	0	1 (< 0.01) 1	-0.01 (-0.02, 0.01)	0.50
Postoperative wound infection	0	2 (0.01) 2	-0.01 (-0.03, 0.01)	0.25
Viral infection	1 (< 0.01) 1	0	0.01 (-0.01, 0.02)	> 0.99
Dengue fever	7 (0.05) 7	9 (0.06) 9	-0.01 (-0.07, 0.04)	0.62
Skin infection	0	1 (< 0.01) 1	-0.01 (-0.02, 0.01)	0.50
Pneumonia bacterial	1 (< 0.01) 1	1 (< 0.01) 1	0.00 (-0.02, 0.02)	> 0.99
Osteomyelitis bacterial	1 (< 0.01) 1	0	0.01 (-0.01, 0.02)	> 0.99
Meningitis tuberculous	1 (< 0.01) 1	0	0.01 (-0.01, 0.02)	> 0.99
Tuberculosis	2 (0.01) 2	1 (< 0.01) 1	0.01 (-0.02, 0.03)	> 0.99
Conjunctivitis	1 (< 0.01) 1	0	0.01 (-0.01, 0.02)	> 0.99
Liver abscess	1 (< 0.01) 1	0	0.01 (-0.01, 0.02)	> 0.99
Abscess limb	1 (< 0.01) 1	0	0.01 (-0.01, 0.02)	> 0.99
Pulmonary tuberculosis	1 (< 0.01) 1	2 (0.01) 2	-0.01 (-0.03, 0.02)	0.62
Gastroenteritis	2 (0.01) 2	2 (0.01) 2	0.00 (-0.03, 0.03)	> 0.99

Preferred Term	Vaccine group (n=15500) n(%) m	Placebo group (n=15490) n(%) m	Rate difference (95% CI)	P value
Sepsis	2 (0.01) 2	2 (0.01) 2	0.00 (-0.03, 0.03)	> 0.99
Cellulitis	3 (0.02) 3	2 (0.01) 2	0.01 (-0.03, 0.04)	> 0.99
Gastric ulcer helicobacter	0	1 (< 0.01) 1	-0.01 (-0.02, 0.01)	0.50
Soft tissue infection	0	1 (< 0.01) 1	-0.01 (-0.02, 0.01)	0.50
Leptospirosis	0	1 (< 0.01) 1	-0.01 (-0.02, 0.01)	0.50
Plasmodium vivax infection	0	1 (< 0.01) 1	-0.01 (-0.02, 0.01)	0.50
Appendicitis	3 (0.02) 3	2 (0.01) 2	0.01 (-0.03, 0.04)	> 0.99
Ovarian cyst	1 (< 0.01) 1	0	0.01 (-0.01, 0.02)	> 0.99
Endometrial hyperplasia	0	1 (< 0.01) 1	-0.01 (-0.02, 0.01)	0.50
Abnormal uterine bleeding	2 (0.01) 2	1 (< 0.01) 1	0.01 (-0.02, 0.03)	> 0.99
Toxic epidermal necrolysis	1 (< 0.01) 1	0	0.01 (-0.01, 0.02)	> 0.99
Stevens-Johnson syndrome	1 (< 0.01) 2	0	0.01 (-0.01, 0.02)	> 0.99
Skin ulcer	1 (< 0.01) 1	1 (< 0.01) 1	0.00 (-0.02, 0.02)	> 0.99
Diabetic foot	4 (0.03) 4	2 (0.01) 2	0.01 (-0.02, 0.05)	0.69
Eyelid ptosis	1 (< 0.01) 1	0	0.01 (-0.01, 0.02)	> 0.99
Cataract	1 (< 0.01) 1	0	0.01 (-0.01, 0.02)	> 0.99
Diabetic blindness	1 (< 0.01) 1	0	0.01 (-0.01, 0.02)	> 0.99
Bipolar disorder	0	1 (< 0.01) 1	-0.01 (-0.02, 0.01)	0.50
Completed suicide	1 (< 0.01) 1	0	0.01 (-0.01, 0.02)	> 0.99
Confusional state	1 (< 0.01) 1	0	0.01 (-0.01, 0.02)	> 0.99
Substance abuse	2 (0.01) 2	0	0.01 (-0.01, 0.03)	0.50

Preferred Term	Vaccine group (n=15500) n(%) m	Placebo group (n=15490) n(%) m	Rate difference (95% CI)	P value
Substance-induced psychotic disorder	1 (< 0.01) 1	0	0.01 (-0.01, 0.02)	> 0.99
Behaviour disorder	1 (< 0.01) 1	0	0.01 (-0.01, 0.02)	> 0.99
Vertigo positional	0	1 (< 0.01) 2	-0.01 (-0.02, 0.01)	0.50
Vestibular disorder	3 (0.02) 3	1 (< 0.01) 1	0.01 (-0.02, 0.04)	0.63
Cholecystitis acute	1 (< 0.01) 1	0	0.01 (-0.01, 0.02)	> 0.99
Hepatic cirrhosis	1 (< 0.01) 1	2 (0.01) 2	-0.01 (-0.03, 0.02)	0.62
Gallbladder rupture	1 (< 0.01) 1	0	0.01 (-0.01, 0.02)	> 0.99
Cholelithiasis	0	1 (< 0.01) 1	-0.01 (-0.02, 0.01)	0.50
Cholangitis	0	1 (< 0.01) 1	-0.01 (-0.02, 0.01)	0.50
Cirrhosis alcoholic	0	1 (< 0.01) 1	-0.01 (-0.02, 0.01)	0.50
Acute kidney injury	0	2 (0.01) 2	-0.01 (-0.03, 0.01)	0.25
Chronic kidney disease	1 (< 0.01) 1	1 (< 0.01) 1	0.00 (-0.02, 0.02)	> 0.99
Azotaemia	1 (< 0.01) 1	0	0.01 (-0.01, 0.02)	> 0.99
Diabetic end stage renal disease	0	1 (< 0.01) 1	-0.01 (-0.02, 0.01)	0.50
Nephrolithiasis	0	2 (0.01) 2	-0.01 (-0.03, 0.01)	0.25
Renal colic	0	1 (< 0.01) 1	-0.01 (-0.02, 0.01)	0.50
Haematuria	1 (< 0.01) 1	0	0.01 (-0.01, 0.02)	> 0.99
Ureterolithiasis	0	2 (0.01) 2	-0.01 (-0.03, 0.01)	0.25
Upper gastrointestinal haemorrhage	3 (0.02) 3	3 (0.02) 3	0.00 (-0.04, 0.04)	> 0.99
Pancreatitis acute	1 (< 0.01) 1	1 (< 0.01) 1	0.00 (-0.02, 0.02)	> 0.99
Peptic ulcer perforation	0	1 (< 0.01) 1	-0.01 (-0.02, 0.01)	0.50

Preferred Term	Vaccine group (n=15500) n(%) m	Placebo group (n=15490) n(%) m	Rate difference (95% CI)	P value
Haemorrhoids	1 (< 0.01) 1	1 (< 0.01) 1	0.00 (-0.02, 0.02)	> 0.99
Inguinal hernia strangulated	1 (< 0.01) 1	0	0.01 (-0.01, 0.02)	> 0.99
Anal stenosis	0	1 (< 0.01) 1	-0.01 (-0.02, 0.01)	0.50
Intestinal ischaemia	0	1 (< 0.01) 1	-0.01 (-0.02, 0.01)	0.50
Gastric haemorrhage	1 (< 0.01) 1	0	0.01 (-0.01, 0.02)	> 0.99
Gastrointestinal haemorrhage	1 (< 0.01) 1	0	0.01 (-0.01, 0.02)	> 0.99
Gastrooesophageal reflux disease	3 (0.02) 3	2 (0.01) 2	0.01 (-0.03, 0.04)	> 0.99
Umbilical hernia	0	1 (< 0.01) 1	-0.01 (-0.02, 0.01)	0.50
Ascites	0	1 (< 0.01) 1	-0.01 (-0.02, 0.01)	0.50
Hypopharyngeal cancer	0	1 (< 0.01) 1	-0.01 (-0.02, 0.01)	0.50
Breast cancer	0	1 (< 0.01) 1	-0.01 (-0.02, 0.01)	0.50
Glioblastoma multiforme	0	1 (< 0.01) 1	-0.01 (-0.02, 0.01)	0.50
Endometrial adenocarcinoma	1 (< 0.01) 1	0	0.01 (-0.01, 0.02)	> 0.99
Uterine leiomyoma	1 (< 0.01) 1	1 (< 0.01) 1	0.00 (-0.02, 0.02)	> 0.99
Cervix carcinoma stage II	1 (< 0.01) 1	0	0.01 (-0.01, 0.02)	> 0.99
Cervix carcinoma stage III	1 (< 0.01) 1	0	0.01 (-0.01, 0.02)	> 0.99
Bronchial carcinoma	0	1 (< 0.01) 1	-0.01 (-0.02, 0.01)	0.50
Tracheal cancer	1 (< 0.01) 1	0	0.01 (-0.01, 0.02)	> 0.99
Testis cancer	0	1 (< 0.01) 1	-0.01 (-0.02, 0.01)	0.50
Hepatocellular carcinoma	2 (0.01) 2	1 (< 0.01) 1	0.01 (-0.02, 0.03)	> 0.99
Lung neoplasm malignant	1 (< 0.01) 1	0	0.01 (-0.01, 0.02)	> 0.99

Preferred Term	Vaccine group (n=15500) n(%) m	Placebo group (n=15490) n(%) m	Rate difference (95% CI)	P value
Metastases to lung	1 (< 0.01) 1	0	0.01 (-0.01, 0.02)	> 0.99
Cholangiocarcinoma	1 (< 0.01) 1	0	0.01 (-0.01, 0.02)	> 0.99
Abdominal neoplasm	0	1 (< 0.01) 1	-0.01 (-0.02, 0.01)	0.50
Metastatic neoplasm	0	1 (< 0.01) 1	-0.01 (-0.02, 0.01)	0.50
Metastatic gastric cancer	1 (< 0.01) 1	0	0.01 (-0.01, 0.02)	> 0.99
Squamous cell carcinoma	1 (< 0.01) 1	0	0.01 (-0.01, 0.02)	> 0.99
Bicytopenia	1 (< 0.01) 1	0	0.01 (-0.01, 0.02)	> 0.99
Blood loss anaemia	1 (< 0.01) 1	2 (0.01) 2	-0.01 (-0.03, 0.02)	0.62
Anaemia	1 (< 0.01) 1	0	0.01 (-0.01, 0.02)	> 0.99
Superior vena cava syndrome	0	1 (< 0.01) 1	-0.01 (-0.02, 0.01)	0.50
Aortic aneurysm	1 (< 0.01) 1	0	0.01 (-0.01, 0.02)	> 0.99
Hypovolaemic shock	0	1 (< 0.01) 1	-0.01 (-0.02, 0.01)	0.50
Extremity necrosis	0	1 (< 0.01) 1	-0.01 (-0.02, 0.01)	0.50
Hypertension	3 (0.02) 3	1 (< 0.01) 1	0.01 (-0.02, 0.04)	0.63
Hypertensive emergency	0	1 (< 0.01) 1	-0.01 (-0.02, 0.01)	0.50

The analysis was based on the participants in the safety analysis set, grouped according to actual intervention. Participants who received at least one dose of Pneucolin® were included in the vaccine group. Data of incidence are shown as n (%) m: n, number of participants; %, proportion of participants; m, number of events.

Table S14. Demographics and clinical profiles of hospitalized SARS-CoV-2 infection (WHO score ≥ 4) in the participants who received at least one dose.

No	Group	Country	Age	Sex	COVID-19 Vaccination History	Underlying disease	WHO Score	NMPA Classification	Profile
1	Placebo	Colombia	81	Male	Inactivated vaccine	Myocardial ischemia, atrial fibrillation, hypertension	5	Severe	Dyspnea; Cough; Diarrhea; General weakness/Fatigue; Headache; Myalgia; Sore throat; Fever; Hypokalemia; Low oxygen saturation (83%); Have imaging characters related to COVID-19.
2	Placebo	Philippines	29	Male	Adenoviral vector vaccine	No	5	Severe	Dyspnea; Anorexia/Nausea/Vomiting; Cough; Myalgia; asthma; Low oxygen saturation (80%); Have imaging characters related to COVID-19.
3	Placebo	Philippines	52	Male	No history	No	5	Severe	Dyspnea; Cough; Chest pain; Epigastric pain; Stomach ulcer; Hypokalemia; Low oxygen saturation (85%); Have imaging characters related to COVID-19.
4	Placebo	South Africa	63	Male	mRNA vaccine	No	4	Mild	Dyspnea; Cough; General weakness/Fatigue; Fever; Sweating.
5	Placebo	Vietnam	39	Female	Adenoviral vector vaccine, mRNA vaccine	No	4	General	Cough; General weakness/Fatigue; Headache; Loss of taste or smell; Sore throat; Hypokalemia; Have imaging characters related to COVID-19.

Table S15. Vaccine efficacy ≥ 15 days after the second dose against symptomatic COVID-19, subgroup analysis.

Subgroup	Total Cases	Vaccine group		Placebo group		Vaccine Efficacy (95%CI)
		No. at risk (person-year)	Incidence density (%)	No. at risk (person-year)	Incidence density (%)	
Per-protocol population						
Age group						
18-59 years	148	10930 (4036.3)	1.6	10978 (4036.4)	2.1	25.4 (-3.3, 46.2)
≥ 60 years	32	1910 (610.2)	2.0	1924 (609.9)	3.3	39.9 (-23.0, 70.6)
Underlying chronic conditions†						
No	138	10911 (4063.3)	1.4	11017 (4078.3)	2.0	30.0 (1.7, 50.2)
Yes	42	1929 (583.3)	3.3	1885 (568.0)	4.0	19.4 (-48.0, 56.1)
Country						
Philippines	56	10014 (4089.6)	0.5	10030 (4083.1)	0.9	40.0 (-3.0, 65.1)
South Africa	37	1295 (298.5)	5.4	1307 (301.0)	7.0	23.1 (-47.5, 59.8)
Colombia	85	1465 (246.3)	15.0	1472 (245.5)	19.6	23.2 (-18.0, 50.0)
Vietnam	2	66 (12.2)	8.2	93 (16.6)	6.0	-42.0 (NA, 91.1)
Modified intention-to-treat-two population						
Age group						
18-59 years	149	11037 (4055.0)	1.6	11096 (4059.2)	2.1	26.3 (-2.0, 46.7)
⩾ 60 years	32	2017 (636.0)	1.9	2019 (632.3)	3.2	40.3 (-22.2, 70.8)
Underlying chronic conditions†						
No	139	11035 (4088.5)	1.4	11137 (4103.0)	2.0	31.1 (3.2, 50.9)
Yes	42	2019 (602.5)	3.2	1978 (588.5)	3.9	19.2 (-48.4, 56.0)
Country						
Philippines	56	10025 (4093.8)	0.5	10045 (4089.6)	0.9	40.0 (-3.1, 65.1)
South Africa	37	1435 (331.2)	4.8	1448 (332.7)	6.3	23.3 (-46.9, 60.0)
Colombia	86	1527 (253.5)	14.6	1527 (252.4)	19.4	24.8 (-15.2, 51.0)

Vietnam	2	67 (12.4)	8.1	95 (16.8)	5.9	-42.9 (-2184.0, 91.1)

Primary efficacy was assessed 14 days after the second dose. A symptomatic COVID-19 case was defined as a participant who met the protocol-specified criteria for a suspected case and had at least one positive reverse-transcriptase–polymerase-chain-reaction (RT-PCR) assay for severe acute respiratory syndrome coronavirus 2 (SARS-CoV-2) RNA.

†Underlying chronic conditions were those diseases that were ongoing at baseline and could increase the risk of SARS-CoV-2 infection.

Project Name: A Phase III Clinical Trial of Influenza Virus Vector COVID-19 Vaccine for Intranasal Spray (DelNS1-2019-nCoV-RBD-OPT1)

Study Title: A Global, Multi-center, Randomized, Double-blind, Placebo-controlled Phase III Clinical Trial to Evaluate the Protective Efficacy and Safety of Influenza Virus Vector COVID-19 Vaccine for Intranasal Spray (DelNS1-2019-nCoV-RBD-OPT1) in Adults Aged 18 Years and Older

Name of Investigational Vaccine: Influenza Virus Vector COVID-19 Vaccine for Intranasal Spray (DelNS1-2019-nCoV-RBD-OPT1)

Sponsor: Beijing Wantai Biological Pharmacy Enterprise Co., Ltd.

Protocol No.: COVID-19-PRO-003

Protocol Version No.: 3.0

Protocol Version Date: July 20, 2022

Authorized Signatory of the Protocol: [redacted] (Signature)

Post of signatory: [redacted]

Signature date: [redacted]

Statement of Confidentiality

All information contained within this protocol is the property of Beijing Wantai Biological Pharmacy Enterprise Co., Ltd. Therefore, the document is being provided to investigators, co-investigators, the Ethics Review Committee, regulatory authorities and relevant institutions for the purpose of review only. Without a prior written permission of the sponsor, except for offering necessary explanation during signing an informed consent form by subjects who might participate in this study, disclosure of any information to a third party unrelated to this study is strictly prohibited.

Protocol Synopsis

**Title**	A Global, Multi-center, Randomized, Double-blind, Placebo-controlled Phase III Clinical Trial to Evaluate the Protective Efficacy and Safety of Influenza Virus Vector COVID-19 Vaccine for Intranasal Spray (DelNS1-2019-nCoV-RBD-OPT1) in Adults Aged 18 Years and Older
**Short Title**	A phase III clinical trial of influenza virus vector COVID-19 vaccine for intranasal spray (DelNS1-2019-nCoV-RBD-OPT1)
**Protocol No.**	COVID-19-PRO-003
**Study Phase**	Phase III
**Study Background**	The novel coronavirus pneumonia (COVID-19) caused by the novel coronavirus (SARS-CoV-2) is a new acute respiratory infectious disease and has become a major global public health event. Globally, several COVID-19 vaccines have been approved for conditional marketing or emergency use and large-scale vaccination is being carried out. The phase I/II clinical trials (ChiCTR2000037782/ChiCTR2000039715) of COVID-19 candidate vaccine, i.e., influenza virus vector COVID-19 vaccine for intranasal spray (DelNS1-2019-nCoV-RBD-OPT1) produced by Wantai BioPharm, have been completed in China, demonstrating that the candidate vaccine has good safety and immunogenicity in healthy adults aged 18 years and older. The sponsor plans to conduct an international multi-center, randomized, double-blind, placebo-controlled phase III clinical trial to further determine the efficacy and safety of this candidate vaccine in adults aged 18 years and older.
**Study Vaccines**	● **Investigational vaccine: Influenza Virus Vector COVID-19 Vaccine for Intranasal Spray**<br>Appearance: packaged in vials, liquid preparation<br>Dosage form: intranasal spray<br>Strength:0.2 mL/dose<br>Active ingredient: influenza virus of receptor binding domain (RBD) gene of recombinant SARS-CoV-2<br>Route of vaccination: intranasal<br>Vaccination dose: 0.1 mL each nasal cavity, totaling 0.2 mL each time<br>Immunization schedule: one each dose at Day 0 and Day 14, 2 doses in total<br>Storage condition: ≤ -15℃<br>Shelf life: 24 months<br>Manufacturer: Beijing Wantai Biological Pharmacy Enterprise Co., Ltd.<br>● **Control vaccine: Placebo of Influenza Virus Vector COVID-19 Vaccine for Intranasal Spray**<br>Identical with the investigational vaccine in dosage form, appearance, and method of vaccination, etc., other than that it does not contain the active ingredient of the vaccine.<br>All investigational vaccines and placebos will be provided by the sponsor.
**Primary study hypothesis**	DelNS1-2019-nCoV-RBD-OPT1 can prevent symptomatic COVID-19 of any severity.

Primary objective	Primary endpoint
● **Efficacy**	
1. To evaluate the protective efficacy of DelNS1-2019-nCoV-RBD-OPT1 for preventing virologically confirmed (reverse transcription polymerase chain reaction [RT-PCR]-positive) symptomatic COVID-19.	1. Virologically confirmed (RT-PCR-positive) symptomatic COVID-19 cases of any severity that occurs for the first time at least 14 days (≥ 15 days) after the second vaccination.
● **Safety**	
1. To evaluate the safety of DelNS1-2019-nCoV-RBD-OPT1.	1. All serious adverse events (SAEs), other medically attended AEs (MAAEs), and adverse events of special interest (AESIs) from the first vaccination to 12 months after the second vaccination in all subjects;
	2. All solicited adverse events within 7 days following the first and second vaccinations in all subjects;
	3. All unsolicited adverse events that occur during the interval between doses and within 30 days after the second vaccination in all subjects.
**Secondary objectives**	**Secondary endpoints**
● **Efficacy**	
1. To respectively evaluate the protective efficacy of DelNS1-2019-nCoV-RBD-OPT1 for preventing virologically confirmed (RT-PCR-positive) symptomatic COVID-19 in subjects with and without COVID-19 vaccination history.	1. Virologically confirmed (RT-PCR-positive) symptomatic COVID-19 cases of any severity that occurs for the first time at least 14 days (⩾ 15 days) after the second vaccination in subjects with or without COVID-19 vaccination history previously.
2. To evaluate the protective efficacy of DelNS1-2019-nCoV-RBD-OPT1 against severe COVID-19.	2. Virologically confirmed (RT-PCR-positive) severe and above COVID-19 cases that occurs for the first time at least 14 days (≥ 15 days) after the second vaccination.
3. To evaluate the protective efficacy of DelNS1-2019-nCoV-RBD-OPT1 against symptomatic COVID-19 of any severity in different age groups.	3. Virologically confirmed (RT-PCR-positive) symptomatic COVID-19 cases of any severity that occur for the first time at least 14 days after the second vaccination (≥ 15 days) in subjects aged 18-59 and ≥60 years.
4. To evaluate the protective efficacy of DelNS1-2019-nCoV-RBD-OPT1 against virologically confirmed (RT-PCR-positive) COVID-19 deaths.	4. Virologically confirmed (RT-PCR-positive) death cases resulting from COVID-19 that occur at least 14 days after the second vaccination (≥ 15 days).

5. To evaluate the protective efficacy of DelNS1-2019-nCoV-RBD-OPT1 against symptomatic COVID-19 of any severity in patients with chronic diseases.	5. Virologically confirmed (RT-PCR-positive) symptomatic COVID-19 cases of any severity that occur for the first time at least 14 days after the second vaccination (≥ 15 days) in subjects with a clear chronic disease.
**Exploratory Objective**	**Exploratory endpoints**
● **Efficacy**	
1. To evaluate the protective efficacy of DelNS1-2019-nCoV-RBD-OPT1 against symptomatic COVID-19 of any severity at least 14 days after the first vaccination (≥15 days).	1. Virologically confirmed (RT-PCR-positive) symptomatic COVID-19 cases of any severity that occur for the first time at least 14 days after the first vaccination (≥15 days).
2. To evaluate the protective efficacy of DelNS1-2019-nCoV-RBD-OPT1 against symptomatic COVID-19 of different severities.	2. Virologically confirmed (RT-PCR-positive) symptomatic COVID-19 cases of different severities that occur for the first time at least 14 days after the second vaccination (≥ 15 days).
3. To evaluate the protective efficacy of DelNS1-2019-nCoV-RBD-OPT1 against symptomatic COVID-19 of any severity in all subjects with symptomatic COVID-19 during the follow-up period.	3. Virologically confirmed (RT-PCR-positive) symptomatic COVID-19 cases of any severity that occur for the first time after the first vaccination, including subjects with symptomatic COVID-19 that occur during the observation period of non-endpoint cases (interval between doses or within 14 days after the second vaccination).
4. To evaluate the protective efficacy of DelNS1-2019-nCoV-RBD-OPT1 against symptomatic COVID-19 infections of any severity in different countries.	4. Virologically confirmed (RT-PCR-positive) symptomatic COVID-19 cases of any severity that occurs for the first time at least 14 days after the second vaccination (≥ 15 days) in different countries.
5. To evaluate the protective efficacy of DelNS1-2019-nCoV-RBD-OPT1 against symptomatic COVID-19 of any severity caused by the variant of concern (VOC).	5. Virologically confirmed (RT-PCR-positive) symptomatic COVID-19 cases of any severity that occurs for the first time and caused by the variant of concern (VOC) at least 14 days after the second vaccination (≥ 15 days).
6. To evaluate the protective efficacy of DelNS1-2019-nCoV-RBD-OPT1 for preventing virologically confirmed (RT-PCR-positive) influenza A.	6. Virologically confirmed (RT-PCR-positive) influenza A cases of any severity that occur for the first time at least 14 days (≥ 15 days) after the second vaccination.

7. To evaluate the protective efficacy of DelNS1-2019-nCoV-RBD-OPT1 for preventing virologically confirmed (RT-PCR-positive) influenza A subtypes H1 and/or H3.	7. Virologically confirmed (RT-PCR-positive) influenza A subtypes H1 and/or H3 subjects of any severity that occur for the first time at least 14 days (≥ 15 days) after the second vaccination.
● **Safety**	
1. To assess the safety of DelNS1-2019-nCoV-RBD-OPT1 from the perspective of vaccine-enhanced disease (VED).	1. Vaccine-enhanced disease (VED) events experienced by subjects with virologically confirmed (RT-PCR-positive) symptomatic COVID-19 cases throughout the study period;
**Study design**	This will be case-driven, multi-center, randomized, double-blind, placebo-controlled study to evaluate the efficacy and safety of DelNS1-2019-nCoV-RBD-OPT1. The study subjects will be adults aged 18 years or above [stratified according to COVID-19 vaccination (marketed or investigational) or not and age]. Subjects enrolled in each clinical study site will be randomized into the study group or the placebo group in a 1:1 ratio to be vaccinated with DelNS1-2019-nCoV-RBD-OPT1 or placebo, respectively.<br>**Safety follow-up:**<br>Solicited adverse events (AEs) within 7 days following the first and second vaccinations and unsolicited AEs from after the first vaccination to 30 days after the last vaccination will be collected from all subjects by combining active monitoring with spontaneous reporting. SAEs, MAAEs and AESIs will also be monitored from the first vaccination to 1 year after the second vaccination.<br>**Protective efficacy follow-up:**<br>Protective efficacy follow-up at each study site will be started after the first dose, and ended when the subjects of the study site were unblinded.<br>In order to assess the safety from the perspective of VED, regardless of the occurrence of vaccine-associated enhanced respiratory disease (VAERD) or antibody-dependent enhancement (ADE), the follow-up period may need to be extended. The study includes an interim analysis and final analysis of safety and efficacy.<br>In each study site, when the last subject of the study site has been followed up for six months after the second dose (with a window period of +15 days), all the subjects of the study site should be unblinded (if the approval date by the local Ethical Review Committee is later than this date, the unblinding should be carried out within 2 months after the approval as far as possible) and cross-over of the placebo group will be conducted. That is, subjects in the placebo group could choose to cross over to receive the investigational vaccine (DelNS1-2019-nCoV-RBD-OPT1), or they could choose to discontinue vaccination and withdraw from the study, and such subjects would be considered to have completed the study. COVID-19 case monitoring will be ceased after the unblinding in the site, and SAE/MAAE/AESI will be collected via telephone follow-up or other

	communication methods at least once every 4 weeks. Subjects can also actively report SAE/MAAE/AESI through the completion of the study. The blinded subjects in other study sites will continue to be followed up for vaccine efficacy and safety as originally specified. The visit schedule after placebo cross-over is specified in Appendix 3.<br>In order to ensure more accurate determination of the collected COVID-19 cases, an Endpoint Adjudication Committee (EAC) will be set up for this study for central independent evaluation and judgment of the endpoint cases. In addition, an Independent Data Monitoring Committee (IDMC) will be set up to review the safety data and protective efficacy data after vaccination.
**Study population**	Adults aged 18 years and older. The study population is grouped according to whether they have previously received COVID-19 vaccines: those who have not received any COVID-19 vaccine (marketed or investigational), those who have received at least one dose of other COVID-19 vaccines (marketed or investigational) with an interval of ≥ 6 months between the last dose and the date when the subjects sign the informed consent for this study, with the number of subjects in each group accounting for about 50% of the total; moreover, subjects are also grouped according to the age of subjects: 18 ~ 59 years old and ≥ 60 years old. The proportion of the subjects aged 60 years and above in each vaccine group is at least 20%, and the proportion of the Chinese/East Asians is not less than 20% of the total enrolled population.
**Indication**	This vaccine is used to prevent the novel coronavirus pneumonia (COVID-19) caused by SARS-CoV-2 infection.
**Sample size**	The target primary endpoint cases are determined based on the following assumptions:<br>1) The vaccine efficacy (VE) for preventing virologically-confirmed COVID-19 is 60%.<br>2) The power to reject H_0 hypothesis is approximately 90%, H_0:VE ≤ 30%.<br>3) The type 1 error rate for evaluating vaccine VE is one-sided α = 2.5%.<br>4) The randomization ratio of DelNS1-2019-nCoV-RBD-OPT1 group to placebo group is 1:1.<br>Based on the above assumptions, the target primary endpoint cases for this trial are 150 firstly confirmed COVID-19 cases.<br>Assuming a 6-month incidence rate of 0.85% in the placebo group, and a drop-out rate of 20%, 32,000-40,000 subjects are planned to be enrolled.<br>The sample size is estimated based on various assumptions before the initiation of the clinical trial. The actual sample size of enrollment may be adjusted according to the actual morbidity, vaccination rate, and the proportion of subjects that meet the protocol analysis conditions in each country.
**Interim analysis**	The number of primary endpoint cases triggering the interim analysis is 75. In interim analysis, the primary efficacy and safety endpoints will be evaluated. The criterion for a successful interim analysis is when the lower bound of the vaccine efficacy (VE) confidence interval corresponding to

	the alpha assigned to interim analysis is greater than 30%. For specific information such as analysis methods, please see the Statistical Analysis Plan (SAP).
**Definition of COVID-19 cases**	**Definition of confirmed COVID-19 cases**<br>In accordance with NMPA *Diagnosis and Treatment Protocol for Novel Coronavirus Pneumonia* (Trial Version Eight, Amendment) and the relevant WHO requirements, for this study, a COVID-19 case for protective efficacy endpoint assessment is defined as meeting both of the following two criteria:<br>1) The patient has newly experienced one or more of the following symptoms:<br>1. Any two or more of the following symptoms (persisting ≥ 2 days): fever (oral temperature≥ 38.0℃ or ear temperature ≥ 38.0℃ or axillary temperature≥ 37.8℃); sore throat; weakness generalized/fatigue (if two symptoms appear simultaneously, only one symptom is counted); rhinitis; myalgia; headache; anorexia/nausea/vomiting (if three symptoms appear simultaneously, only one symptom is counted); diarrhea; mental status changes.<br>2. Any one or more of the following symptoms: cough (persisting≥2 days); loss of taste or smell (persisting≥2 days); dyspnea.<br>3. Clinical or imaging evidences of COVID-19 (if any).<br>2) Positivity for SARS-CoV-2 nucleic acid test (i.e., RT-PCR-positive).<br>**Definition of primary endpoint case**<br>Subjects meeting both of the above two criteria for the first time at least 14 days (≥15 days) after the second vaccination.<br>COVID-19 cases confirmed from the first vaccination to 14 days after full schedule will not be defined as primary endpoint cases for this study.<br>Subjects who are confirmed from after the first vaccination to before the second vaccination can choose whether to continue the second vaccination based on their willingness provided that the criteria for the second vaccination are met.
**Clinical study sites**	Approximately 50 sites
**Study duration**	The estimated duration of the entire study is 20 months.

List of Abbreviations

Abbreviations	English Name
ADE	Antibody-Dependent Enhancement
AE	Adverse Event
AESI	Adverse Event of Special Interest
AVR	Adverse Reaction
CI	Confidence Interval
COVID-19	Corona Virus Disease 2019
CRF	Case Report Form
CRO	Contract Research Organization
IDMC	Independent Data Monitoring Committee
EAC	Endpoint Adjudication Committee
eCRF	Electronic Case Report Form
EDC	Electronic Data Capture System
ERC	Ethics review committee
GCP	Good Clinical Practice
HIV	Human Immunodeficiency Virus
ICF	Informed Consent Form
ICH	International Council for Harmonisation
ID	Identification Number
IgA	Immunoglobulin A
IgG	Immunoglobulin G
ITT	Intent-to-Treat
MAAE	Medically Attended Adverse Event
MedDRA	Medical Dictionary for Regulatory Activities
MERS	Middle East Respiratory Syndrome
MERS-CoV	Middle East Respiratory Syndrome-Coronavirus
mITT1	Modified Intent-To-Treat 1
mITT2	Modified Intent-To-Treat 2
mL	Milliliter
mm	Millimeter
NMPA	National Medical Products Administration
PCR	Polymerase Chain Reaction
PI	Principal Investigator
PP	Per-protocol
RBD	Receptor Binding Domain
RNA	Ribonucleic Acid
RT-PCR	Reverse Transcription Polymerase Chain Reaction
SAE	Serious Adverse Event
SAP	Statistic Analysis Plan
SARS	Severe Acute Respiratory Syndrome
SARS-CoV	Severe Acute Respiratory Syndrome-Coronavirus

SOP	Standard Operating Procedure
SUSAR	Suspected Unexpected Serious Adverse Reaction
VAERD	Vaccine-Associated Enhanced Respiratory Disease
VE	Vaccine Efficacy
VED	Vaccine-Enhanced Disease
WHO	World Health Organization

Table of Contents

Protocol Synopsis **2**

List of Abbreviations **8**

Table of Contents **10**

1 Study Background and Rationale **15**

1.1 Study Background 15

1.1.1 Pathogens *15*

1.1.2 Clinical manifestations *15*

1.1.3 Route of transmission *16*

1.1.4 Risk factors *16*

1.1.5 Clinical management guidelines *16*

1.2 Introduction to Investigational Vaccine 16

1.3 Overview of Phase I/II Clinical Trials 17

1.4 Study Rationale 18

1.5 Potential Risks of the Investigational Vaccine 18

1.5.1 Risk of DelNS1-2019-nCoV-RBD-OPT1 vaccination *18*

1.5.2 Vaccine enhanced disease *18*

1.5.3 Pregnant population *19*

1.5.4 Risk of personal medical information leakage *19*

1.5.5 Risks for Biological Samples Collection *19*

1.6 Potential Benefits of Participating in the Trial 19

2 Study Hypotheses, Study Objectives, Endpoints and Case Definitions **20**

2.1 Primary Study Hypothesis 20

2.2 Study Objectives and Endpoints 20

2.2.1 Primary objective *20*

2.2.2 Secondary objectives *20*

2.2.3 Exploratory objectives *21*

2.2.4 Primary endpoints *21*

2.2.5 Secondary endpoints *22*

2.2.6 Exploratory endpoints *22*

2.3 Definition of COVID-19 Cases 23

3 Study Design **23**

3.1 Overall Study Design 23

3.2 Study Criteria 25
3.3 Randomization and Blinding 25
3.3.1 Randomization 25
3.3.2 Blinding 26
3.3.3 Emergency unblinding 26
3.3.4 Rules for unblinding 27
4 Study Subjects 27
4.1 Description of Subject Population 27
4.2 Inclusion Criteria 27
4.3 Exclusion Criteria 28
4.4 Criteria for Postponement of the Second Vaccination 29
4.5 Criteria for Exclusion from the Second Vaccination 29
4.6 Discontinuation Criteria 30
4.7 Temporary Suspension or Early Termination of the Study 31
4.8 Definition of end of study 31
5 Study Vaccines 31
5.1 Acquisition of Vaccine 32
5.2 Components, Appearance, Package and Label of Vaccine 32
5.3 Storage and Transport of Vaccines 32
5.4 Immunization Route 33
5.5 Immunization Schedule 33
5.6 Dose Tracking 33
5.7 Accountability and Disposal Procedures for Study Vaccines 33
5.8 Concomitant Medications 33
5.8.1 Permitted concomitant medication 34
5.8.2 Prohibited concomitant medication 34
6 Study Procedures 34
6.1 Study Procedures and Evaluation 34
6.1.1 V1-screening visit (D-7 to D-1) 34
6.1.3 V3 - Visit for the second vaccination (D14 + 14d window period) 36
6.1.4 V4 - Follow-up Visit (V3+30d, + 14d window period) 37
6.1.5 Follow-up visits during study period 37
6.1.6 Temporary contacts and unscheduled visits 38
6.1.7 Birth control and pregnancy management during the study 38

6.2 Monitoring Procedures of COVID-19 Cases 38
6.3 Follow-up of Confirmed COVID-19 Cases 39
6.4 Safety Monitoring 40
6.5 Laboratory Evaluation 41
6.5.1 Collection, transportation and storage of samples 41
6.5.2 Detection of samples 42
6.5.3 Future use of preserved samples; correlation analysis 42
7 Safety Assessment and Reporting 42
7.1 Definitions Related to AE 42
7.1.1 Adverse events (AEs) 42
7.1.2 Adverse vaccine reactions (AVR) 43
7.1.3 Solicited adverse events 43
7.1.4 Unsolicited adverse events 43
7.1.5 Medically-attended adverse event (MAAE) 43
7.1.6 AEs of Special Interest (AESI) 43
7.1.7 Serious adverse event (SAE) 45
7.1.8 Suspected unexpected serious adverse reaction (SUSAR) 45
7.2 Reporting Period and Parameters 45
7.3 Severity of Adverse Events (AEs) 45
7.4 Correlation of Adverse Events (AEs) 45
7.5 Follow-up of Adverse Events (AEs) 46
7.6 COVID-19-related AEs for VED Assessment 47
7.7 General Principles for Recording of Adverse Events (AEs) 47
7.8 Reporting of SAEs 47
7.8.1 Investigator's responsibilities 47
7.8.2 Sponsor's responsibilities 48
7.9 Pregnancy-Related Events 48
7.10 Safety Monitoring 49
7.11 Noncompliance with Regulations or Requirements 49
8 Data Management and Data Retention 49
8.1 Data Management 49
8.1.1 Data coding 50
8.1.2 Data validity 50
8.1.3 Source data verification 50

8.1.4 Relevant definitions ... *50*
8.2 Data Collection Method (Compilation and Completion of Case Report Forms) ... 50
8.3 Data Storage ... 51
8.4 Data Locking Procedure ... 51
8.5 Study Record Retention ... 51
8.6 Protocol Deviation ... 51
9 General Statistical Consideration ... 51
9.1 Sample Size ... 52
9.2 Definition of Analysis Population ... 53
9.2.1 Enrolled population ... *53*
9.2.2 Safety analysis population ... *53*
9.2.3 Intent-to-treat (ITT) population ... *53*
9.2.4 Per-protocol (PP) population ... *54*
9.3 Interim Analysis and Risk Monitoring ... 54
9.4 Placebo Cross-over Design ... 55
9.5 Analysis Methods ... 55
9.5.1 Descriptive analysis ... *55*
9.5.2 Change to analysis plan ... *55*
9.5.3 Analysis of baseline and demographic characteristics and subject compliance ... *55*
9.5.4 Analysis of primary endpoints ... *56*
9.5.5 Analysis of secondary endpoints ... *56*
9.6 Safety Analysis ... 56
9.7 Processing of Dropouts and Missing Values ... 57
10 Quality Assurance and Quality Control ... 57
10.1 General Principles ... 58
10.2 Clinical Monitoring ... 58
10.3 Third Party Audits ... 58
10.4 Inspection by Management Agencies ... 59
10.5 Endpoint Adjudication Committees (EAC) ... 59
10.6 Independent Data Monitoring Committee (IDMC) ... 59
11 Ethical Considerations (and Informed Consent) ... 59
11.1 Ethical Standards ... 59
11.2 Ethical Review ... 60

11.3 Informed Consent Process 60

11.4 Confidentiality 60

11.5 Compensation........ 61

11.6 Risks and Benefits........ 61

11.6.1 Risks Faced by Investigators 62

11.7 Reporting of Infectious Diseases 62

11.8 Study-related Injury Treatment and Compensation Methods 62

12 Publication and data sharing policy........ 62

Appendix 1 Visit Schedule........ 64

Appendix 2 Table of severity grading of adverse events 67

Appendix 3 Placebo Cross-over Design 71

Appendix 4 WHO Criteria for Grading of COVID-19 Cases [1]........ 73

Appendix 5 Clinical Classification of COVID-19 issued by National Health Commission of the People's Republic of China........ 74

Appendix 6 Country-specific requirements........ 75

1 Study Background and Rationale

1.1 Study Background

The novel coronavirus pneumonia (COVID-19) refers to pneumonia caused by infection with novel coronavirus (2019-nCoV or SARS-CoV-2). This infectious disease was firstly discovered by humans in December 2019 and has rapidly spread to more than 210 countries and regions around the world. In January 2020, the World Health Organization (WHO) declared the COVID-19 epidemic to be a public health emergency of international concern, and subsequently announced in March 2020 that COVID-19 has the characteristics of a pandemic. Currently, several COVID-19 vaccines have been approved for conditional marketing or emergency use and large-scale vaccination is being carried out globally.

1.1.1 Pathogens

SARS-CoV-2 (2019-nCoV) is a β coronavirus that is enveloped, round or oval, and 60-140nm in diameter. It has 5 essential genes targeting four structural proteins, namely nucleoprotein (N), viral envelope (E), matrix protein (M) and spike protein (S), and ribonucleic acid (RNA)-dependent RNA polymerase. The nucleoprotein (N) wraps the RNA genome to form a nucleocapsid, surrounded by a viral envelope (E), and the viral envelope encapsules proteins such as matrix protein (M) and spike protein (S). Spike protein enters the cell by binding to angiotensin converting enzyme 2. When isolated and cultured in vitro, SARS-CoV-2 was detectable at around 96 hours in human respiratory tract epithelial cells, and around 4-6 days in Vero E6 and Huh-7 cell lines.

The virus is sensitive to ultraviolet and heat. Exposure to 56°C for 30 minutes and lipid solvents such as ether, 75% ethanol, chlorine-containing disinfectant, peracetic acid, and chloroform can effectively inactivate the virus. Chlorhexidine has not been effective in inactivating the virus.

1.1.2 Clinical manifestations

The latency period is 1 to 14 days, mostly 3 to 7 days.

The main manifestations include fever, dry cough, and fatigue. Some patients have decline or loss of senses of smell and taste as the first symptoms, and a few patients are accompanied by symptoms such as nasal obstruction, rhinorrhoea, pharyngalgia, conjunctivitis, myalgia, and diarrhea. Severe cases mostly developed dyspnea and (or) hypoxemia after one week. In severe cases, patients progress rapidly to acute respiratory distress syndrome, septic shock, metabolic acidosis that is difficult to correct, blood coagulation dysfunction and multiple organ failure etc. A very small number of patients may also have manifestations such as central nervous system involvement and avascular necrosis of the extremities. It is worth noting that for severe and critically ill patients, their fever could be moderate to low, or even barely noticeable. The patients with mild symptoms may not develop pneumonia but only low fever, mild fatigue, dysosmia and dysgeusia. Few patients have no significant clinical symptom after being infected with novel coronavirus. Most patients have good prognosis and a small number of patients are critically ill, which are commonly observed in elderly patients, patients with chronic underlying

diseases, females in late trimester of pregnancy and perinatal period, and obese population.

1.1.3 Route of transmission

Respiratory droplet and close contact transmission are the main routes of transmission. Contact with virus-contaminated items can also cause infection. Long-time exposure to high concentrations of aerosols in relatively closed environments may cause aerosol transmission. Since the novel coronavirus can be isolated from feces and urine, attention should be paid to the contact transmission or aerosol transmission due to environmental pollution caused thereby.

1.1.4 Risk factors

According to current clinical expertise and related epidemiological investigations, risk factors for COVID-19 disease include but are not limited to: age > 65 years, asthma, chronic kidney disease, chronic lung disease, hypertension, diabetes, weakened immunity, living in a nursing home or long-term care facility, and severe obesity.

1.1.5 Clinical management guidelines

At present, no antiviral drugs have been found to be effective in a strict "randomized, double-blind, placebo-controlled study", but some drugs may have certain therapeutic effects through clinical observation studies. For patients with mild symptoms, careful monitoring of clinical deterioration at home is recommended. Severe cases require hospitalization, especially if patients need supplemental oxygen therapy or advanced support, including high-flow oxygen therapy, CPAP, mechanical ventilation, and dialysis. There is currently no specific treatment for COVID-19. Many countries around the world have formulated local clinical management guidelines compatible to their resources.

1.2 Introduction to Investigational Vaccine

The vaccine strain of DelNS1-2019-nCoV-RBD-OPT1 is an attenuated influenza virus strain of recombinant 2019-nCoV RBD gene, which is constructed by cloning the RBD coding sequence of spike protein of 2019-nCoV to deleted-NS1 site of attenuated, cold-adapted influenza virus strain, and has good safety. The skeleton of the virus genome originated from an influenza A (H1N1) virus strain that was isolated in 2009, which has the characteristics of widespread, weakly pathogenic and sensitive to antiviral drugs such as oseltamivir. The virus loses its natural immune function of interfering host cells due to NS1 deletion and fails to effectively proliferate in normal cells. The vaccine strain is an attenuated, cold-adapted influenza strain, and its optimum growth temperature is about 33°C, but its replication is limited at 37°C. This ensures that the virus is safer. The vaccine strain that carries B2M secretion signal peptide and codon-optimized RBD coding sequence can express a large amount of 2019-nCoV RBD protein and secrete it extracellularly after infecting the host cell, which facilitate to stimulate the immune system to produce protective immune response.

This product is prepared by inoculating MDCK cells with influenza virus strain of recombinant 2019-nCoV RBD gene, culturing, harvesting, purifying and adding appropriate stabilizer. It is used for prophylaxis of diseases caused by 2019-nCoV. This product has been completed for the preparation of cell bank and passed the inspection by the National Institutes for Food and

Drug Control with COA; it has been completed for the preparation of virus bank, and the toxicity evaluation of the master virus seed lot is qualified in ferret, and the virus working seed lot have been tested qualified by the National Institutes for Food and Drug Control; it has been completed for the 40-layer cell factory preparation process of virus, the crude purification process through membrane system and the refine process through column chromatography, and the virus preparation process; it has been completed for the development of verification methods for vaccine study and specification study; it has been completed for the production of continuous batches of vaccines for clinical application and vaccines for clinical use, and is sampled to the National Institutes for Food and Drug Control for verification; it has undergone pharmacotoxicological evaluation of acute toxicity, allergic reaction, repeated-dose toxicity, tissue distribution of virus, immunogenicity and immunoprotection of vaccine.

This vaccine is a liquid preparation, which is stored at -15℃ and below and administered by nasal spray. Nasal spray administration is simple and convenient for rapid large-scale population immunization, which has less systemic adverse reactions. Similar to the respiratory infection pathway of COVID-19 virus, this vaccine can simulate the natural respiratory mucosal infection pathway of virus, well stimulate the mucosal immune response mediated by immunoglobulin A (IgA) antibody, and reduce the potential ADE risk mediated by immunoglobulin G (IgG) antibody. It is theoretically an ideal vaccine to prevent 2019-nCoV.

1.3 Overview of Phase I/II Clinical Trials

The phase I clinical trial of DelNS1-2019-nCoV-RBD-OPT1 adopted a single-center, randomized, double-blind, placebo-controlled design, and a total of 63 subjects were enrolled, including 32 subjects in the Stage 1 (18-59 years old) adult group and 31 subjects in the Stage 2 (≥60 years old) elderly group. The subjects in the two stages completed the full-course vaccination on September 17, 2020 and October 1, 2020 (two doses on Day 0 and Day 14), respectively. The interim analysis has currently been completed. The results of the analysis showed that there was a total of 63 subjects in the safety evaluation data set (no dropout or removal), including 51 subjects in the study group and 12 subjects in the placebo group. The overall incidence of adverse reactions was 19.6% (10/51) and 16.7% (2/12) in the study group and the placebo group, respectively. Among them, the incidence of local adverse reactions was 5.9% (3/51) and 8.3% (1/12), while the incidence of systemic adverse reactions was 13.7% (7/51) and 16.7% (2/12), respectively. There were no adverse events or SAEs of grade 3 or above among the subjects in the two groups. All adverse reactions were mild and could spontaneously recover in a short period of time. The results of the safety analysis showed no statistically significant difference between the two groups (P>0.05). There were no obvious clinically significant abnormal changes in laboratory parameters before and 3 days after each vaccination. The overall seroconversion rate of peripheral blood specific cellular immunity of per protocol subjects in the study group after vaccination was 70.8% (34/48), the seroconversion rate of serum anti-novel coronavirus IgG antibody was 25.5% (13/51), and the seroconversion rate of nasopharyngeal swab anti-novel coronavirus IgA antibody was 15.7% (8/51).

The phase II clinical trial of DelNS1-2019-nCoV-RBD-OPT1 adopted a single-center, randomized, double-blind, placebo-controlled design, and enrolled a total of 724 healthy subjects aged 19-86 years, including 362 subjects in the Day 0-14 immunization schedule group and 362 subjects in the Day 0-21 immunization schedule group. A total of 708 subjects completed the full-course vaccination (the full-course vaccination rate was 97.8%). The current results showed that the overall incidence of adverse reactions was 22.6% (55/243) and 21.8% (26/119) in the Day 0-14 immunization schedule study group and the placebo group, respectively; the overall incidence of adverse reactions was 19.4% (47/242) and 25.0% (30/120) in the Day 0-21 immunization schedule study group and the placebo group, respectively. No serious adverse events associated with vaccination occurred during the trial. The overall seroconversion rate of peripheral blood specific cellular immunity of per protocol subjects in the Day 0-14 and Day 0-21 immunization schedule study groups was 55.2% (127/230) and 59.7% (138/231), respectively; the seroconversion rate of serum anti-novel coronavirus IgG antibody was 11.9% (28/236) and 8.6% (20/232), respectively; the seroconversion rate of nasopharyngeal swab anti-novel coronavirus IgA antibody was 13.1% (31/236) and 11.2% (26/232), respectively.

In summary, the results of phase I/II clinical trials have preliminarily shown that this product has good safety and immunogenicity.

1.4 Study Rationale

This study aims to provide data support for registration application of the preventive vaccine, i.e., influenza virus vector COVID-19 vaccine for intranasal spray (DelNS1-2019-nCoV-RBD-OPT1), against COVID-19 so that approval of the regulatory authorities can be obtained. The data will also support the WHO prequalification application. The study sites selected for this study are located in areas where the population is at a relatively high risk.

1.5 Potential Risks of the Investigational Vaccine

1.5.1 Risk of DelNS1-2019-nCoV-RBD-OPT1 vaccination

Like any vaccination, DelNS1-2019-nCoV-RBD-OPT1 may cause allergic reactions. In order to effectively prevent and control this potential risk, the study staff will observe the safety of patients for at least 30 minutes after each vaccination. Recipients of this product may experience rhinorrhoea, nasal obstruction, sore throat, sneezing, fever, headache, asthenia, dizziness, cough, chills and nausea. There may be other currently unknown adverse reactions.

1.5.2 Vaccine enhanced disease

Animals vaccinated with SARS or MERS-CoV candidate vaccines may experience pathological enhancement during challenge tests in theory, that is, lung lymphocyte and eosinophil infiltration, which is similar to the situation observed in infants undergoing respiratory syncytial virus enhancement after being immunized with formalin inactivated vaccines. Some researchers believe that this immunopathological response is the result of a dominant Th2-type response to vaccine antigens, but there is no direct evidence to prove this. Although preclinical studies of non-human primates infected with SARS-CoV-2 after

immunization with DelNS1-2019-nCoV-RBD-OPT1 have not found similar histopathology, the enhanced respiratory disease [called vaccine-associated enhanced respiratory disease (VAERD)] requires close monitoring and follow-up of all participants to determine possible severe respiratory infections that may occur during or after the confirmed COVID-19 infection, and are consistent with the pathogenesis of severe COVID-19, including non-respiratory consequences. In addition to the risks that may be associated with VAERD, the antibody-dependent enhancement (ADE) of the disease has been emphasized as another mechanism of VED, that is, antibodies produced after vaccination may increase the uptake of the virus by the host cells, thereby resulting in an immune response to promote rather than prevent the disease.

1.5.3 Pregnant population

The safety of DelNS1-2019-nCoV-RBD-OPT1 for pregnant women has not been assessed, so this product is not suitable for pregnant women.

1.5.4 Risk of personal medical information leakage

To ensure the confidentiality of all collected information of study participants, the following safeguard measures will be taken:

1) Access to study files and personal information is only restricted to study personnel, Ethics Review Committee, regulatory authorities and the sponsor;
2) The study information files should be uniformly stored in the reference room of the project, and the file manager will manage the files and keep the key to the reference room;
3) All biological samples will be affixed with a unique study identification number without personally identifiable information.

1.5.5 Risks for Biological Samples Collection

Venipuncture is a routine clinical procedure commonly used to collect blood samples in the medical field. Direct complications may include mild pain during skin puncture, and rare dizziness and fainting. In addition, venipuncture may cause hematoma, but the risk is very low. Skin/soft tissue infections at the puncture site, vein, or bloodstream can occur, but are very rare in finger blood draws and venous blood draws. The associated risks that may be caused by the nasopharyngeal/oropharyngeal swabs collection process are very low. After being wiped by the swab, some people may cough and sneeze for a short period of time, and a few people may feel irritation or have slight bleeding in the nasal passages.

Under the strict supervision of the investigators, qualified and experienced medical personnel or health workers should collect venous blood specimens, nasopharyngeal/oropharyngeal swabs according to the SOPs after being trained, so as to minimize the pain and risk of subjects (including local pain, very rare infection at venipuncture site and nasal mucosa damage).

1.6 Potential Benefits of Participating in the Trial

All study participants will gain the following benefits:

1) All volunteers will receive free physical examinations, regardless of whether they are

eventually enrolled. The results of all physical examinations will be notified to the volunteers. In the event of a newly diagnosed disease, the volunteer will be referred to an appropriate medical and health institution.

2) After the product's efficacy is confirmed (the vaccine has been confirmed with statistically significant protective efficacy) or its efficacy/safety data are sufficient to support conditional marketing approval or emergency use and it is approved by the country where the study is conducted, subjects in the placebo group will be vaccinated with DelNS1-2019-nCoV-RBD-OPT1 free of charge.

3) Participating in this study will help better understand the COVID-19 disease so as to develop better preventive measures. If DelNS1-2019-nCoV-RBD-OPT1 can successfully prevent COVID-19, all subjects will make a significant contribution to the progress of global public health.

2 Study Hypotheses, Study Objectives, Endpoints and Case Definitions

2.1 Primary Study Hypothesis

DelNS1-2019-nCoV-RBD-OPT1 can prevent symptomatic COVID-19 of any severity.

2.2 Study Objectives and Endpoints

2.2.1 Primary objective

2.2.1.1 Efficacy

To evaluate the protective efficacy of DelNS1-2019-nCoV-RBD-OPT1 for preventing virologically confirmed (RT-PCR-positive) symptomatic COVID-19.

2.2.1.2 Safety

To evaluate the safety of DelNS1-2019-nCoV-RBD-OPT1.

2.2.2 Secondary objectives

2.2.2.1 Efficacy

1) To respectively evaluate the protective efficacy of DelNS1-2019-nCoV-RBD-OPT1 for preventing virologically confirmed (RT-PCR-positive) symptomatic COVID-19 in subjects with and without COVID-19 vaccination history;

2) To evaluate the protective efficacy of DelNS1-2019-nCoV-RBD-OPT1 against severe COVID-19;

3) To evaluate the protective efficacy of DelNS1-2019-nCoV-RBD-OPT1 against symptomatic COVID-19 of any severity in different age groups;

4) To evaluate the protective efficacy of DelNS1-2019-nCoV-RBD-OPT1 against virologically -confirmed (RT-PCR-positive) COVID-19 deaths;

5) To evaluate the protective efficacy of DelNS1-2019-nCoV-RBD-OPT1 against symptomatic COVID-19 of any severity in patients with chronic diseases.

2.2.3 Exploratory objectives

2.2.3.1 Efficacy

1) 1. To evaluate the protective efficacy of DelNS1-2019-nCoV-RBD-OPT1 against symptomatic COVID-19 of any severity at least 14 days after the first vaccination (≥15 days);
2) To evaluate the protective efficacy of DelNS1-2019-nCoV-RBD-OPT1 against symptomatic COVID-19 of different severities;
3) To evaluate the protective efficacy of DelNS1-2019-nCoV-RBD-OPT1 against symptomatic COVID-19 of any severity in all subjects with symptomatic COVID-19 during the follow-up period;
4) To evaluate the protective efficacy of DelNS1-2019-nCoV-RBD-OPT1 against symptomatic COVID-19 of any severity in different countries;
5) To evaluate the protective efficacy of DelNS1-2019-nCoV-RBD-OPT1 against symptomatic COVID-19 of any severity caused by the variant of concern (VOC);
6) To evaluate the protective efficacy of DelNS1-2019-nCoV-RBD-OPT1 for preventing virologically confirmed (RT-PCR-positive) influenza A.
7) To evaluate the protective efficacy of DelNS1-2019-nCoV-RBD-OPT1 for preventing virologically confirmed (RT-PCR-positive) influenza A subtypes H1 and/or H3.

2.2.3.2 Safety

1) To assess the safety of DelNS1-2019-nCoV-RBD-OPT1 from the perspective of vaccine-enhanced disease (VED).

2.2.4 Primary endpoints

2.2.4.1 Efficacy

Virologically confirmed (RT-PCR-positive) symptomatic COVID-19 cases of any severity that occurs for the first time at least 14 days (≥ 15 days) after the second vaccination.

2.2.4.2 Safety

1) All serious adverse events (SAEs), other medically attended AEs (MAAEs), and adverse events of special interest (AESIs) from the first dose to 12 months after the second dose in all subjects;
2) All solicited adverse events within 7 days following the first and second vaccinations in all subjects;
3) All unsolicited adverse events that occur during the interval between doses and within 30 days after the second vaccination in all subjects.

2.2.5 Secondary endpoints

2.2.5.1 Efficacy

1) Virologically confirmed (RT-PCR-positive) symptomatic COVID-19 cases of any severity that occurs for the first time at least 14 days (⩾ 15 days) after the second vaccination in subjects with or without COVID-19 vaccination history previously.
2) Virologically confirmed (RT-PCR-positive) severe and above COVID-19 cases that occurs for the first time at least 14 days (≥ 15 days) after the second vaccination.
3) Virologically confirmed (RT-PCR-positive) symptomatic COVID-19 cases of any severity that occur for the first time at least 14 days after the second vaccination (≥ 15 days) in subjects aged 18-59 and ≥60 years.
4) Virologically confirmed (RT-PCR-positive) death cases resulting from COVID-19 that occur at least 14 days after the second vaccination (≥ 15 days).
5) Virologically confirmed (RT-PCR-positive) symptomatic COVID-19 cases of any severity that occur for the first time at least 14 days after the second vaccination (≥ 15 days) in subjects with a clear chronic disease.

2.2.6 Exploratory endpoints

2.2.6.1 Efficacy

1) Virologically confirmed (RT-PCR-positive) symptomatic COVID-19 cases of any severity that occur for the first time at least 14 days after the first vaccination (≥15 days);
2) Virologically confirmed (RT-PCR-positive) symptomatic COVID-19 cases of different severities that occur for the first time at least 14 days after the second vaccination (≥ 15 days);
3) Virologically confirmed (RT-PCR-positive) symptomatic COVID-19 cases of any severity that occur for the first time after the first vaccination, including subjects with symptomatic COVID-19 that occur during the observation period of non-endpoint cases (interval between doses or within 14 days after the second vaccination);
4) Virologically confirmed (RT-PCR-positive) symptomatic COVID-19 cases of any severity that occurs for the first time at least 14 days after the second vaccination (≥ 15 days) in different clinical study sites (countries).
5) Virologically confirmed (RT-PCR-positive) symptomatic COVID-19 cases of any severity that occurs for the first time and caused by the variant of concern (VOC) at least 14 days after the second vaccination (≥ 15 days).
6) Virologically confirmed (RT-PCR-positive) influenza A cases of any severity that occur for the first time at least 14 days (≥ 15 days) after the second vaccination.
7) Virologically confirmed (RT-PCR-positive) influenza A subtypes H1 and/or H3 subjects of any severity that occur for the first time at least 14 days (≥ 15 days) after the second

vaccination.

2.2.6.2 Safety

1. Vaccine-enhanced disease (VED) events experienced by subjects with virologically confirmed (RT-PCR-positive) symptomatic COVID-19 throughout the study period.

2.3 Definition of COVID-19 Cases

Definition of confirmed COVID-19 cases

In accordance with NMPA Diagnosis and Treatment Protocol for Novel Coronavirus Pneumonia (Trial Version Eight, Amendment) and the relevant WHO requirements, for this study, a COVID-19 case for protective efficacy endpoint assessment is defined as meeting both of the following two criteria:

1) The patient has newly experienced one or more of the following symptoms:

1. Any two or more of the following symptoms (persisting ≥ 2 days): fever (oral temperature≥ 38.0°C or ear temperature ≥ 38°C or axillary temperature≥ 37.8°C); sore throat; weakness generalized/fatigue (if two symptoms appear simultaneously, only one symptom is counted); rhinitis; myalgia; headache; anorexia/nausea/vomiting (if three symptoms appear simultaneously, only one symptom is counted); diarrhea; mental status changes.
2. Any one or more of the following symptoms: cough (persisting ≥ 2 days); loss of taste or smell (persisting ≥ 2 days); dyspnea.
3. Clinical or imaging evidences of COVID-19 (if any).

2) Positivity for SARS-CoV-2 nucleic acid test (i.e., RT-PCR-positive).

Definition of primary endpoint case

Subjects meeting both of the above two criteria for the first time at least 14 days (≥15 days) after the second vaccination.

COVID-19 cases confirmed from the first vaccination to 14 days after full schedule will not be defined as primary endpoint cases for this study.

Subjects who are confirmed from after the first vaccination to before the second vaccination can choose whether to continue the second vaccination based on their willingness provided that the criteria for the second vaccination are met.

3 Study Design

3.1 Overall Study Design

This will be case-driven, multi-center, randomized, double-blind, placebo-controlled study to evaluate the efficacy and safety of DELNS1-2019-nCoV-RBD-OPT1. The study subjects are adults aged 18 years and above (stratified according to COVID-19 vaccination or not and age). The study population is grouped according to whether they have previously received COVID-19 vaccines: those who have not received any COVID-19 vaccine (marketed or investigational),

those who have received at least one dose of other COVID-19 vaccines (marketed or investigational) with an interval of ⩾ 6 months between the last dose and the date when the subjects sign the informed consent for this study, with the number of subjects in each group accounting for about 50% of the total; moreover, subjects are also grouped according to the age of subjects: 18 ~ 59 years old and ⩾ 60 years old. The proportion of the subjects aged 60 years and above in each group is at least 20%, and the proportion of the Chinese/East Asians is not less than 20% of the total enrolled population. Subjects enrolled in each clinical study site will be randomized into the study group or the placebo group in a 1:1 ratio to be vaccinated with DelNS1-2019-nCoV-RBD-OPT1 or placebo, respectively.

Safety follow-up:

Solicited adverse events (AEs) within 7 days following the first and second vaccinations and unsolicited AEs from after the first vaccination to 30 days after the last vaccination will be collected from all subjects by combining active monitoring with spontaneous reporting. SAEs, MAAEs and AESIs will also be monitored from the first vaccination to 1 year after the second vaccination.

Protective efficacy follow-up:

Protective efficacy follow-up will be started after the first dose, and ended when the subjects of the study site were unblinded.

In order to assess the safety from the perspective of VED, regardless of the occurrence of vaccine-associated enhanced respiratory disease (VAERD) or antibody-dependent enhancement (ADE), the follow-up period may need to be extended. The study includes an interim analysis and final analysis of safety and efficacy.

In each study site, when the last subject of the study site has been followed up for six months after the second dose (with a window period of +15 days), all the subjects of the study site should be unblinded (if the approval date by the local Ethical Review Committee is later than this date, the unblinding should be carried out within 2 months after the approval as far as possible) and cross-over of the placebo group will be conducted. That is, subjects in the placebo group could choose to cross over to receive the investigational vaccine (DelNS1-2019-nCoV-RBD-OPT1), or they could choose to discontinue vaccination and withdraw from the study, and such subjects would be considered to have completed the study. COVID-19 case monitoring will be ceased after the unblinding in the site, and SAE/MAAE/AESI will be collected via telephone follow-up or other communication methods at least once every 4 weeks. Subjects can also actively report SAE/MAAE/AESI through the completion of the study. The blinded subjects in other study sites will continue to be followed up for vaccine efficacy and safety as originally specified. The visit schedule after placebo cross-over is specified in Appendix 3.

In order to ensure more accurate determination of the collected COVID-19 cases, an Endpoint Adjudication Committee (EAC) will be set up for this study for central independent evaluation and judgment of the endpoint cases. In addition, an Independent Data Monitoring Committee

(IDMC) will be set up to review the safety data and protective efficacy data after vaccination.

3.2 Study Criteria

The point estimate of vaccine efficacy (VE) should be at least 50%, meeting the minimum requirements given in the WHO target product profile. The success of the protective analysis will be determined by the lower limit of the adjusted 95% confidence interval (CI), and the lower limit of the confidence interval for the primary endpoint should exceed 30%.

The 6-month incidence rate of COVID-19 in the placebo group is expected to be approximately 0.85%. This is a case-driven trial, and the primary analysis will be triggered by accumulation of primary endpoints in the two groups.

This trial assumes that the target vaccine efficacy (VE) is 60%, and a power of 90% will reject the null hypothesis that VE ≤ 30%. The efficacy of the vaccine will be estimated using 1-HR. HR is the hazard ratio estimated based on the Cox proportional hazards model. If the efficacy criteria have been met in interim analysis (approximately 50% of the events have been confirmed), conditional marketing approval can be applied for. If DelNS1-2019-nCoV-RBD-OPT1 is found to be ineffective in the interim analysis, the IDMC may declare it as ineffective. In this case, enrollment will be stopped (if the enrollment is not yet completed). The IDMC can provide recommendations for continuous safety monitoring of subjects in the study group or all subjects for an additional limited period. If neither the efficacy nor the ineffectiveness criteria are met in the interim analysis before the enrollment is completed, the enrollment of the remaining volunteers will be continued.

Severe and above COVID-19 cases of and COVID-19 deaths will be monitored at all trial sites. Although this study may lack sufficient statistical inference capacity regarding the efficacy of the vaccine against serious illness and death, the results will be presented as a secondary endpoint.

3.3 Randomization and Blinding

This is a global, multi-center, randomized, double-blind, placebo-controlled parallel-group study.

3.3.1 Randomization

Enrollment will be performed online through the electronic data capture (EDC) system, and randomization will be completed after subjects are confirmed to meet the inclusion criteria and before vaccination. In order to achieve a relatively balanced different strata between the vaccine group and the placebo group, this trial adopts a stratified blocked randomization design. Site documentation will include the identity number (ID), date of birth, medical history, date of screening visit, registration status and location (if applicable), and reasons for exclusion from the study (if applicable) of subjects. All eligible subjects will be stratified by study site, COVID-19 vaccination (marketed or investigational) or not and age (18-59 years and ≥ 60 years), and be randomized to the study group or placebo group in a 1: 1 ratio. The randomization plan will be generated and maintained by the randomization statistician. The

subjects will be randomly grouped online through the central randomization and drug supply management system, and the randomization system will assign a unique random number to the subjects based on their demographic information and enrollment qualification data entered in EDC to determine the grouping of subjects.

3.3.2 Blinding

After demographic information and screening data are entered into the system and eligibility is confirmed, each subject will be assigned a unique random number and vaccine number for the corresponding group. The investigator responsible for distributing the vaccine during randomization will distribute the vaccine to the investigator responsible for vaccination according to the designated number. All investigators will remain blind during the trial, and the blind code will be kept by an independent randomization statistician.

Due to the particularity of cross-contamination of the investigational vaccine, the vaccination room areas of the study group and the placebo group should be distinguished and isolated. In order to maintain the blindness of this trial, it is planned to set up at least 4 mutually independent vaccination rooms at each study site, which will be numbered. Subjects in each room will be inoculated with the same vaccine (investigational vaccine or placebo). When the vaccine number is generated, the system will also generate the corresponding vaccination room number. Subjects should go to the designated vaccination room for vaccination according to the assigned number, followed by the post-vaccination safety observation. Subjects in each vaccination room should be observed in the same area and subjects in different vaccination rooms should not be observed in the same area. Relevant information which may indicate the room number allocated for subjects shall be recovered and sealed by independent personnel on the day of vaccination. The specific operation of randomization and related blinding maintenance will be implemented in accordance with the standard operating procedures (SOP) or equivalent documents uniformly formulated in this study.

Adequate measures will be adopted in this study to ensure and maintain blindness. To ensure that the trial is carried out correctly, the unblinded staff of the random drug delivery system or other unblinded personnel who need to know this information can have access to the vaccine distribution information. IDMC members may also be unblinded due to the need to review the safety and efficacy of vaccines.

3.3.3 Emergency unblinding

Only in an emergency or in the event of a serious adverse event and when knowledge of the study vaccine information is vital to the clinical care or wellbeing of the subject can emergency unblinding for an individual case be performed after confirmation by the principal investigator and sponsor. In such case, a person designated for the study region who is allowed to have access to the subject blind codes will log into the central randomization system to implement emergency unblinding while keeping the relevant records. The subject with the study number will be discontinued from the trial and treated as a drop-out case, and the investigator will record the reasons for the discontinuation on the electronic case report form (eCRF).

If the investigator believes that knowing the subject's vaccine assignment information will be beneficial to the subject's health and safety, even if the subject does not have a major safety risk, the investigator can contact the medical monitors to discuss the need for unblinding. If the subject has a major safety risk, the investigator should be able to perform emergency unblinding by himself, but must notify the Medical Monitor and the sponsor as soon as possible afterwards.

3.3.4 Rules for unblinding

The overall blinding of the study will be maintained until database lock or the conditions for cross-over vaccination in the placebo group have been met and related data has been cleaned. Emergency unblinding can be performed when it is necessary to know the group to which an individual subject is assigned due to an SAE so that corresponding emergency treatment can be given.

After the study is completed or the sponsor decides to perform cross-over for the placebo group or the study is terminated, data cleaning and database lock will be performed by the data manager. After data lock, the project statistician will apply to the sponsor for unblinding. Blind codes will be available to the project statistician upon approval by the sponsor.

4 Study Subjects

4.1 Description of Subject Population

It is planned to enroll 32,000-40,000 adults ≥ 18 years old.

The final determination of eligibility for enrollment will depend on the medical history, clinical examination and other screening results, compliance with all the inclusion criteria and noncompliance with any exclusion criteria, a full understanding of the study content, and signing of informed consent.

Based on surveillance data and epidemiological models, adults of any age whose location or circumstances increase their risk of exposure to SARS-CoV-2 and COVID-19 will be recruited for the trial. The recruited volunteers should be regionally representative to a certain extent and support the universality of the data results, including healthy volunteers and volunteers at high risk of COVID-19, such as the elderly ≥60 years old and patients suffering from hypertension, diabetes, obesity, chronic kidney disease, chronic obstructive pulmonary disease, chronic heart disease and other chronic diseases who are in stable condition under drug control.

If the investigator considers necessary, the assessment of relevant screening procedures may be repeated for the subject at Visit 1 (D-7 ~ D-1), but it is only allowed to repeat once, and the finally accepted results should be recorded in EDC.

4.2 Inclusion Criteria

Subjects participating in this study need to meet all the following criteria:

1) Aged ≥18 years old at the time of enrollment;

2) Be able to comply with the requirements of clinical study protocol and complete all trial

procedures, and sign informed consent form;

3) Subjects who have not received any COVID-19 vaccine (marketed or investigational), those who have received at least one dose of other COVID-19 vaccines (marketed or investigational) with an interval of ⩾ 6 months between the last dose and the date when the subjects sign the informed consent for this study;

4) Those who are negative for HIV screening (depending on the relevant policy of the country where the trial is conduct, if qualification for HIV testing is required in the country, this information will be obtained mainly by inquiry while protecting the subject's privacy);

5) Fertile males and females of childbearing potential who are willing to take appropriate contraceptive measures from signing ICF to 3 months after the last dose, including abstinence or effective contraceptive measures (e.g., intrauterine or implantable contraceptive devices, oral contraception, combination of contraceptive diaphragm or condom with contraceptive gel); women of childbearing potential should be negative for pregnancy test on the day of vaccination (if applicable).

6) Healthy people or people with a mild underlying disease that has remained stable without exacerbation (not requiring hospitalization or without major modification of the treatment regimen) within at least 3 months prior to inclusion in the study.

4.3 Exclusion Criteria

Subjects who meet any one of the following criteria will be excluded from this study:

1) Prior history of COVID-19, or SARS-CoV-2 RT-PCR-positive at screening;

2) Positive test result of SARS-CoV-2-specific antibody at screening [Only applicable to subjects without vaccination history of COVID-19 vaccine (marketed or investigational)];

3) Pregnant or lactating women;

4) Fever on the day of vaccination or within 3 days prior to vaccination (oral temperature ≥37.5℃/ear temperature ≥ 37.5℃ / axillary temperature ≥37.3℃);

5) Those who had any acute disease in the past 5 days that requires systemic antibiotic or antiviral treatment (including but not limited to the use of anti-influenza virus drugs such as Tamiflu, Relenza, Symmetrel or Flumadine);

6) Those who had low immune function caused by immunodeficiency diseases, diseases of important organs, cancer, and immune diseases (e.g. Guillain Barre syndrome, systemic lupus erythematosus, rheumatoid arthritis, alienia or splenectomy caused by any condition, and other immune disease that may affect immune response at the investigator's discretion);

7) Long-term use (defined as ≥14 days) of immunosuppressants or other immunomodulators (for glucocorticoids, e.g., ≥10 mg/day prednisone or equivalent dose; inhaled and topical steroids are allowed) within 6 months prior to the first vaccination;

8) History of hemorrhagic diseases (e.g., factor deficiency, thrombocytopenia or other coagulation disorders), or hemorrhagic tendency, or continuous requirement of

anticoagulants;

9) Having been injected with immunoglobulins and/or blood products within 3 months before receiving the investigational vaccine;
10) Received subunit or inactivated vaccine within 14 days before vaccination, or received live attenuated vaccine within 28 days before vaccination;
11) Participation in a clinical trial of another product within 1 month prior to vaccination, or planning to participate in a clinical trial of another product during the study;
12) Having a history of severe allergic reactions or severe adverse reactions from previous immunizations, or allergy to any component of the investigational vaccine;
13) Patients deemed by the investigator as unsuitable for using nasal spray (those with severe rhinitis or nasal deformities, etc.);
14) Planning to relocate permanently from the current area prior to the completion of the study or to leave the current area for a long period (preventing compliance with the prescribed visit schedule) during the study visits;
15) Other conditions that the investigators consider unsuitable for this clinical study.

4.4 Criteria for Postponement of the Second Vaccination

The second vaccination takes place on the 14th day after the first vaccination. Vaccination will be postponed in the event of any of the following during the study:

1. Axillary temperature over 37.3°C (or oral temperature over 37.5°C or the ear temperature over 37.5 ℃) before the second vaccination, waiting until the body temperature returns to normal;
2. Before the second vaccination, the subject has developed an acute disease that has not recovered or has been in the acute phase of a chronic disease, and the investigator has assessed that the acute disease can recover in the short term; If the subject is confirmed with COVID-19 after receiving the first vaccination, only when the COVID-19 has recovered can they receive the second dose.

The second dose of vaccine can be delayed within Day 14+14 days, and subjects will not receive the second dose if it is out of such time frame.

4.5 Criteria for Exclusion from the Second Vaccination

The subject will discontinue vaccination in case of any of the following before the second dose, but may continue with other study procedures at the discretion of the investigator:

1) A positive pregnancy test for female subjects of childbearing potential;
2) Severe hypersensitivity or serious adverse event causally related to vaccination has occurred following the previous vaccination;
3) Other situations where the investigator considers it inappropriate for the subject to continue receiving the second dose.

4.6 Discontinuation Criteria

Withdrawal at the investigator's discretion:

Withdrawal from the study is where the investigator discontinues an enrolled subject from the study in the occurrence of any of the following that make it no longer appropriate for the subject to continue on the study. The reasons may include:

1. The subject has experienced an intolerable adverse event so that the investigator considers that continuing in the trial would be unfavorable for the subject's wellbeing.
2. A major protocol violation has occurred that may affect the subject's safety;
3. Other reasons that an investigator judges not suitable for the continued participation in the study.

Subject's voluntary withdrawal:

According the ICF and local regulations, the subject has the right to withdraw prematurely from the study at any time during the study, or the subject may not specifically request withdrawal, but has not been followed up as per the trial requirements or has been lost to follow-up.

For subjects who withdraw early, the investigator should make every effort to contact the subject, record the reason for withdrawal on the eCRF, and inform the study team.

Individual subjects may be withdrawn from the study for the following reasons:

- Withdrawal of informed consent by the subject;
- Complete loss to follow-up (after three failed contact attempts), after three failed contact attempts, the investigator can decide whether to continue contact with the subject. If the subject actively contacts the investigator before the end of the study, the last contact date will be the end date of the study;
- Received other COVID-19 vaccination (marketed or investigational);
- Noncompliance with the inclusion criteria or compliance with the exclusion criteria (new or retrospective onset during the study period, or being ignored during the screening process);
- Noncompliance with the study procedures:

If the withdrawal is due to an AE, with the subject's consent, appropriate follow-up or medical care will be arranged until the AE resolves. If the subject withdraws from the study, the data gathered as well as blood (if applicable), nasopharyngeal and/or oropharyngeal samples collected before withdrawal can still be used for analysis and testing. Unless specifically requested by the subject, the blood (if applicable)/nasopharyngeal and/or oropharyngeal samples collected will continue to be stored.

4.7 Temporary Suspension or Early Termination of the Study

If the sponsor has sufficient and appropriate reasons, this study can be temporarily suspended or early terminated. However, a written notice stating the reasons for the suspension or termination of the study should be provided to the investigator, ERC/IRB and regulatory authorities. If the study is early terminated or suspended, the principal investigator (PI) will promptly notify the ERC/IRB and provide reasons for termination or suspension.

The sponsor reserves the right to terminate or reduce this clinical study for any reason, including but not limited to the following situations:

- Investigational vaccine lack of efficacy;
- Safety risks of subjects.
- The scientific issues lose their meaning, or the study objectives cannot be realized (e.g., the accumulation of cases is slow);
- Failure to comply with the GCP or clinical trial protocol;
- Risks that cannot be fully quantified;
- Ethical issues raised by local communities or local medical/health care institutions;
- Failure to correct study defects discovered through site monitoring (e.g., inaccurate and/or incomplete long-term data records, or failure to meet other pre-determined standards and requirements of the sponsor);
- The sponsor decides to stop the development of the investigational vaccine;
- Failure of the enrolled subjects to meet the requirements in terms of quality and/or quantity.

If it is decided at any time to terminate the management of all vaccinated subjects, the PI will promptly notify the ERC/IRB and regulatory authorities.

In addition, based on the safety data review or interim safety analysis results, the IDMC may recommend that the sponsor terminate the study. If the sponsor decides to early terminate the trial for some reason, a summary report should be submitted to the regulatory authorities. The summary report will give a brief overview of the study, and describes the number of subjects vaccinated, the dose and duration of the vaccination, the details of adverse reactions (if applicable), and the reasons for stopping the study or discontinuing the use of the vaccine.

4.8 Definition of end of study

The end of the study refers to the completion of 12-month follow-up after the final dose of the last subject in the vaccine group, or premature termination of the study for various reasons.

5 Study Vaccines

The investigational vaccine in this study is influenza virus vector COVID-19 vaccine for intranasal spray (DelNS1-2019-nCoV-RBD-OPT1), and placebo is used as control.

Table **1** Introduction to the Study Vaccines

Investigational vaccine:

Name:	Influenza Virus Vector COVID-19 Vaccine for Intranasal Spray
Dosage form:	Intranasal spray
Ingredient per dose:	[redacted]
Appearance:	Packaged in vials, liquid preparation
Specification:	0.2 mL/dose
Storage condition:	≤-15°C
Shelf life:	24 months
Manufacturer:	Beijing Wantai Biological Pharmacy Enterprise CO., LTD.

Placebo:

Name:	Placebo of Influenza Virus Vector COVID-19 Vaccine for Intranasal Spray
Dosage form:	Intranasal spray
Ingredient per dose:	[redacted]
Appearance:	Packaged in vials, liquid preparation
Specification:	0.2 mL/dose
Storage condition:	≤-15°C
Shelf life:	24 months
Manufacturer:	Beijing Wantai Biological Pharmacy Enterprise CO., LTD.

The placebo is identical with investigational vaccine in dosage form, appearance and method of inoculation, etc., other than that it does not contain the active ingredient of vaccine.

All investigational vaccine and placebo will be provided by the sponsor.

5.1 Acquisition of Vaccine

The vaccines will be provided by the sponsor and will be shipped directly to the study site in a verified shipping container under frozen condition.

5.2 Components, Appearance, Package and Label of Vaccine

See the Investigator's Brochure and vaccine package insert for details. A label containing the following information will be attached to the study vaccines: vaccine number, manufacturer's name and address, product name, date of manufacture, expiry date, storage conditions (≤-15°C), instructions for use, and any other suitable study vaccine label information required by the jurisdiction where the trial is located. To keep blinded, the appearance of the investigational vaccine and placebo is kept consistent, including the use of same intranasal spray device. Each vaccine has a unique vaccine number. The vaccines used for cross-over of the placebo group will also have their unique vaccine number.

5.3 Storage and Transport of Vaccines

The investigational vaccine should be stored in the freezer at - 15 ℃ and below with temperature monitoring equipment. Only authorized site management personnel can enter the

freezer. Before the start of the study, the sponsor will assess the site vaccine storage conditions. Storage temperature should be continuously monitored with calibrated temperature monitoring device and be recorded according to relevant standard operating procedures (SOPs) or equivalent documents. The study site should be equipped with a temperature alarm system and a spare freezer to cope with unexpected situations such as cold chain interruption. Whether the cold chain interruption occurs before or after the study vaccine is received on site, the sponsor and study monitor must be contacted. Vaccines can be stored for no more than 2 weeks at 2 - 8 ℃ after thawing and should not be thawed for more than 3 times before use.

Before receiving study vaccines at the study site, the sponsor will monitor temperature deviations and fluctuations during shipment. After receiving study vaccines, the study site will be responsible for managing the vaccine storage temperature and provide relevant data to the sponsor on a regular basis. The sponsor will decide whether to manage the site vaccines or whether to destroy the vaccines after a temperature deviation occurs.

5.4 Immunization Route

The vaccine is for intranasal administration only, and a specific intranasal spray device is required (each dose of vaccine is 0.2 mL, 0.1 mL for each nasal cavity each time).

5.5 Immunization Schedule

Two doses will be administered, respectively on Day 0 and Day 14.

5.6 Dose Tracking

Whether the subject receives vaccination and the number of doses will be recorded in the case report form (CRF).

5.7 Accountability and Disposal Procedures for Study Vaccines

The investigational vaccine will be distributed to each study site and handed over to and managed by the site director. Vaccine responsibilities include the quantity of vaccines shipped, adequate and safe handling and use related documents, vaccine temperature records, and plans to return or destroy unused vaccines.

Vaccine administrators should keep complete records of all study vaccines received from the sponsor, accurate records of vaccine numbers and inventory, and accountability records of the study vaccines. Vaccine administrators should also ensure the security of these documents, and ensure that used empty vaccine vials cannot be used for other studies. After the study is over and the monitoring of all study vaccines is completed, the study institution will receive the sponsor’s final disposal instructions for all remaining study vaccines, and the investigator will dispose of and document them as required by the sponsor’s specifications.

5.8 Concomitant Medications

Concomitant medications refer to all medications other than the study vaccines used from subject’s signing of ICF to 30 days after the second dose, including antibiotics, antiviral drugs, antipyretic and analgesic drugs, anti-allergic drugs, biological products (including vaccines,

immunoglobulin, and blood products, etc.), and Chinese (patent) drugs, etc. (except vitamins and/or food supplements for non-therapeutic purposes). Beyond 30 days after the second dose, any therapies for SAE/MAAE/AESI should also be recorded.

All information of concomitant medications, including medication name, medication purpose, dosage and administration, and duration, must be recorded in detail on the eCRF.

5.8.1 Permitted concomitant medication

The following medications are permitted to be used during the study:

1) Drugs for the control of concomitant diseases are allowed to be continued during the study period if the investigator considers that they would not confound interpretation of the trial results;
2) Necessary drug treatment for any AE that the subject has experienced is allowed;
3) Patients diagnosed with COVID-19 after vaccination are allowed to be treated according to local standard.

5.8.2 Prohibited concomitant medication

The following medications are not permitted during the study:

1) Any prophylactic medications for COVID-19 are prohibited (except for treatment for confirmed patients);
2) It is prohibited to use any investigational or unapproved drug or vaccine other than the study vaccines;
3) It is prohibited to receive any other marketed or investigational COVID-19 vaccines;
4) Administration of other vaccines is prohibited from the first dose to 30 days after the second dose (except for emergency vaccines such as tetanus and rabies vaccines);
5) Long-term (continuously for >14 days) use of glucocorticoids (dose ≥10 mg/day prednisone or equivalent) or other immunosuppressants (other than inhaled and topical steroids, or short-term oral steroids for ≤14 days) are prohibited;
6) Immunoglobulin or other blood products (except those used for emergency such as tetanus and rabies, and immunoglobulin for tumor treatment);
7) Subjects should avoid using non-prescription medications such as antipyretics (such as acetaminophen), anti-inflammatory agents (such as ibuprofen and naproxen), and anti-influenza drugs for 12 hours prior to vaccination.

6 Study Procedures

6.1 Study Procedures and Evaluation

Details of study visit date, window period, and study procedures are provided in Appendix 1.

6.1.1 V1-screening visit (D-7 to D-1)

After the subject's informed consent is obtained, several procedures will be completed during

the screening process to determine whether the subject can be selected. Baseline data will be collected within 7 days before the first vaccination; unless otherwise specified for the enrollment criteria, all inclusion and exclusion criteria must be assessed based on the data obtained during this period. The information collected during the screening process (medical history, physical examination and laboratory test results) will be recorded in the original documents and CRF. The specific laboratory reference values of each study site will be included in the site-specific study file. After confirming the eligibility, subjects will be randomly assigned with the vaccine number and complete vaccination as early as possible.

After study information is provided and sufficient informed consent is obtained, the following steps will be performed before registration:

1) Confirming that the written informed consent form is obtained;
2) Assigning subject number after the study-specific informed consent form is signed;
3) Height and weight;
4) Physical examination;
5) Vital signs;
6) Reviewing inclusion/exclusion criteria;
7) Obtaining demographic information materials and contact information (e.g., address, telephone);
8) Obtaining medical history, treatment history, medication history, and vaccination history including:
    - Details of vaccination in the past and vaccination reactions;
    - Surgery history;
    - Previous hospitalization history;
    - History of food/drug allergy;
    - Previous medications;
    - Any history of chronic or recurrent diseases;
    - COVID-19 vaccination history. For the subjects with COVID-19 vaccination history (marketed or investigational), it is necessary to provide the relevant supporting documents for the vaccination and document the time of vaccination, manufacturer and dose of the vaccine in detail;
    - Influenza vaccination history within one year prior to administration of the study vaccines. For the subjects who received influenza vaccine within one year prior to administration of the study vaccines, it is necessary to record in detail the time of vaccination, manufacturer and dose of the vaccine.

9) Detection of SARS-CoV-2-specific antibody (fingertip blood);
10) RT-PCR test for SARS-CoV-2;
11) Performing pregnancy test for women of childbearing potential;
12) HIV screening;
13) Collecting information of concomitant medications.

The screening procedure schedule for any single subject will include a 7-day window period before randomization. Data will be collected using eCRF. The number of days and procedures for site visits (including the allowable window interval) are described in the Visit Schedule (Appendix 1).

6.1.2 V2-First Vaccination Visit (D0)

- Reviewing inclusion/exclusion criteria;
- Physical examination;
- Vital signs;
- Performing pregnancy test for women of childbearing potential (if necessary);
- Randomization;
- Performing intranasal vaccination, and correctly recording the vaccination time and vaccine number after verifying the vaccination information;
- Staying for at least 30-minute observation after vaccination;
- Collecting information of concomitant medications;
- Providing subjects with Diary Card (paper) and/or installing e-PRO in the subject's electronic device;
- Dispensing thermometer to subjects
- Collecting adverse events.

6.1.3 V3 - Visit for the second vaccination (D14 + 14d **window period***)*

- Reviewing inclusion/exclusion criteria;
- Collecting diary card information, and the investigator will check the information recorded on the diary card with the subject;
- Updating the demographic information of the subject (if there are changes);
- Updating the subject's medical history and health status (if necessary);
- Physical examination;
- Vital signs;
- Performing pregnancy test for women of childbearing potential;

- Collecting information on the subject's concurrent medications;
- Performing intranasal vaccination, and correctly recording the vaccination time and vaccine number after verifying the vaccination information;
- Staying for at least 30-minute observation after vaccination;
- Collecting adverse events;
- Providing subjects with Diary Card (paper) and/or installing e-PRO in the subject's electronic device.

6.1.4 V4 - Follow-up Visit (V3+30d, + 14d window period)

- Collecting diary card information, and the investigator will check the information recorded on the diary card with the subject;
- Updating the demographic information of the subject (if there are changes);
- Updating the subject's medical history and health status (if necessary);
- Physical examination;
- Vital signs;
- Collecting information on the subject's concurrent medications;
- Collecting adverse events;
- Installing e-PRO in the subject's electronic device.

6.1.5 Follow-up visits during study period

Through an e-PRO installed on a mobile phone or other electronic device or diary card, the subject will record/report any symptoms that may be related to COVID-19 from after the first vaccination to *V3 + 30 days* (with a window period of +14 days) on a daily basis, and the study staff will weekly make inquiries via telephone or other communication methods. After *V3 + 30 days* (with a window period of +14 days) until the unblinding in the site, subjects are required to complete e-PRO, if applicable, and the study staff will make inquiries every week. The window period for the above remote visits is ±3 days. The subjects are asked whether they have experienced related signs/symptoms, and are reminded to seek medical help and receive relevant COVID-19 tests as soon as possible should any suspected symptoms occur.

Subjects will record/report whether they have experienced any AEs including SAE/MAAE/AESI daily from after the first vaccination to *V3 + 30 days* (with a window period of +14 days) using e-PRO installed on their mobile phone or other electronic devices or the Diary Card. Meanwhile, the study staff will make inquiries every week via telephone or in other ways. After *V3 + 30 days* (with a window period of +14 days) until the unblinding in the site, subjects are required to complete e-PRO, if applicable, and the study staff will make inquiries at least once every 4 weeks to record SAEs, MAAEs, and AESIs. The window period for the above remote visits is ±3 days.

6.1.6 Temporary contacts and unscheduled visits

Temporary contacts and unscheduled visits can be conducted according to the subject's request or when deemed necessary by the investigator and other designated personnel. All temporary contacts and unscheduled visits should be recorded in the subject's study records and CRF.

6.1.7 Birth control and pregnancy management during the study

Before a female subject of childbearing age is enrolled and receives the investigational vaccine, her contraceptive status should be assessed and recorded. Appropriate methods of contraception include barrier contraception, hormonal contraception, intrauterine device, surgical infertility, and abstinence. If a female subject becomes pregnant after enrollment, she will be encouraged to complete the remaining visits and study procedures (except the vaccination procedure), unless there is a medical contraindication. The investigator must inform the sponsor [or the Contract Research Organization (CRO) designated by the sponsor] within 24 hours of knowledge of pregnancy. For any subject who becomes pregnant between vaccination and the last study visit, the pregnancy outcome will continue to be tracked in the study, even if it occurs after the subject's expected study end time. The pregnancy and its outcome will be recorded in the pregnancy CRF. If the subject's pregnancy outcome is childbirth, the investigator will follow up until the newborn is 1 year old after birth.

6.2 Monitoring Procedures of COVID-19 Cases

All subjects will be followed up for protective efficacy from after the first dose until the unblinding in the site. Confirmed COVID-19 cases will be captured by both active and passive monitoring. All subjects will install e-PRO on their mobile phones or other electronic devices or use diary cards to record/report suspected COVID-19 symptoms, and will receive a contact card containing the investigator's contact information and designated medical institutions.

Each study site will establish a 24/7 communication service to maintain contacts with the subjects. During the post-vaccination self-safety observation training process, subjects will be told to call the service in the event of any disease.

Through an e-PRO installed on a mobile phone or other electronic device or diary card, the subject will record/report any symptoms that may be related to COVID-19 from after the first vaccination to *V3 + 30 days* (with a window period of +14 days) on a daily basis, and the study staff will weekly make inquiries via telephone or other communication methods. After *V3 + 30 days* (with a window period of +14 days) until the unblinding in the site, subjects are required to complete e-PRO, if applicable, and the study staff will make inquiries for any related signs/symptoms every week. The window period for the above remote visits is ±3 days.

SARS-CoV-2 nucleic acid test (RT-PCR) will be triggered by any of the following during the study:

1. Any two or more of the following symptoms (persisting ≥ 2 days): fever (oral temperature≥ 38.0°C or ear temperature ≥ 38 ℃ or axillary temperature≥ 37.8°C); sore throat; weakness generalized/fatigue (if two symptoms appear simultaneously, only one symptom is

counted); rhinitis; myalgia; headache; anorexia/nausea/vomiting (if three symptoms appear simultaneously, only one symptom is counted); diarrhea; mental status changes.

2. Any one or more of the following symptoms: cough (persisting≥2 days); loss of taste or smell (persisting≥2 days); dyspnea.
3. Clinical or imaging evidences of COVID-19 (if any).

For subjects who have the above suspected COVID-19 symptoms, nasopharyngeal swabs should in principle be collected in the designated hospital/health agency within 72 hours, but if it's undoable, nasopharyngeal swabs should be collected timely upon the notice of such suspected cases; (If nasopharyngeal swabs cannot be collected, oropharyngeal swabs should be collected, or additional oropharyngeal swabs can be collected in the same collection tube based on local policy. If nasopharyngeal swabs are not collected, please note the reason), or by the investigator at his/her home or using other effective sampling methods at the investigator's discretion. Two samples (one nasopharyngeal swab each in one nasal cavity) will be collected, one for immediate SARS-CoV-2 RT-PCR testing by the laboratory of the study institution and another for central laboratory as backup sample (The first sample collected each time shall be used as a backup sample; A backup sample must contain at least a nasopharyngeal swab unless no nasopharyngeal swab has been collected). For tests performed at the study institution's laboratory, if the test result is negative but the symptoms persist, a second sampling test should be performed within 3 to 5 days (two samples required, one nasopharyngeal swab each in one nasal cavity). If the second sampling test result remains negative, the test will not be repeated. If the test result is positive, it will be followed as COVID-19 confirmed cases (refer to section 6.3 below). SARS-CoV-2 RT-PCR retest and RT-PCR test of influenza A viruses will be performed for samples sent to the central laboratory. The test results of the central laboratory will be regarded as the final results.

6.3 Follow-up of Confirmed COVID-19 Cases

For subjects confirmed as COVID-19 cases, the date of onset of symptoms related to COVID-19 will be taken as the date of onset, and exploratory study of the sequence of SARS-CoV-2 variants will be carried out for confirmed COVID-19 cases.

Subjects confirmed as COVID-19 cases will be managed and treated according to the local policy. After being confirmed with COVID-19, the subject will be followed up by the investigator every 7-10 days until RT-PCR is negative and the symptoms have resolved (If symptoms persist after two consecutive negative test results, investigators will decide whether to continue sampling for RT-PCR testing). Nasopharyngeal swabs of subjects will be collected during follow-up visits (If nasopharyngeal swabs cannot be collected, oropharyngeal swabs should be collected, or additional oropharyngeal swabs can be collected in the same collection tube based on local policy. If nasopharyngeal swabs are not collected, please note the reason). Two samples will be collected each time (one nasopharyngeal swab each in one nasal cavity): one for immediate SARS-CoV-2 RT-PCR testing by the laboratory of the study institution and another for central laboratory as backup sample (The first sample collected each time shall be used as a backup sample; A backup sample must contain at least a nasopharyngeal swab unless

no nasopharyngeal swab has been collected). The treatment status and medical history of the subjects will be collected to determine whether the subject meets the criteria for severe or critical COVID-19.

The type, time of onset and reporting time of the subject's symptoms, the sampling time, testing time and reporting time of nucleic acid test, the time of confirmed diagnosis, and the outcome, etc., will be recorded and form relevant records.

See Figure 1 for the detailed flow chart.

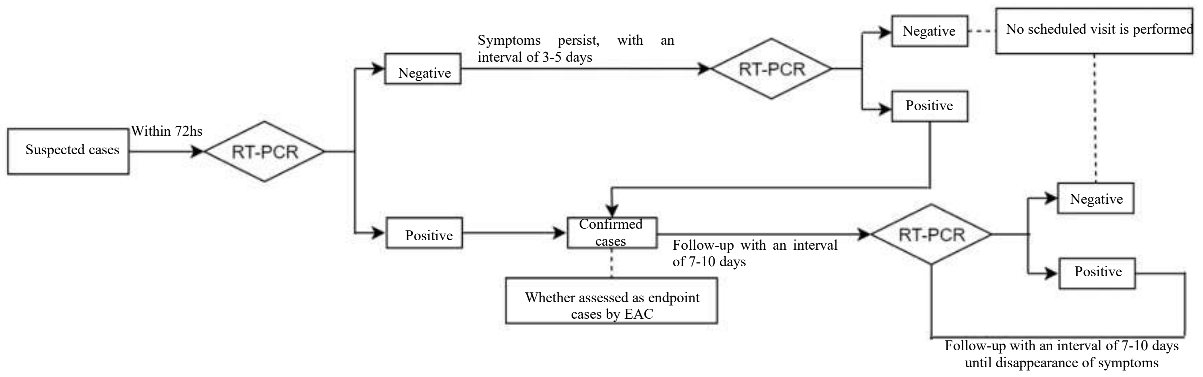

Figure1 Follow-up flowchart of COVID-19 cases

6.4 Safety Monitoring

Safety assessment is an important part of this trial and the common primary endpoint. Two independent safety aspects will be monitored.

1) Safety monitoring related to vaccination:

- Subjects should stay for at least 30 minutes of observation after vaccination. At least 30 minutes after vaccination, the axillary temperature (unit °C, accurate to 0.1°C) will be measured and reactions at the vaccination site will be assessed. Any immediate post-vaccination adverse reactions will be described and recorded in detail [Note: The site investigator (PI or other designated personnel) can decide whether the subject needs further site safety observation];
- Subjects will be asked to fill in the diary card (electronic or paper) to collect solicited AEs that occur within 7 days after each vaccination and unsolicited AEs that occur after the first dose until 30 days after the last dose of vaccination. At the same time, subjects will be followed up for SAEs, MAAEs and AESIs developing from the first dose to 1 year after the second dose. Subjects will record/report whether they have experienced any AEs including SAE/MAAE/AESI daily from after the first vaccination to *V3 + 30 days* (with a window period of +14 days) using e-PRO installed on their mobile phone or other electronic devices or the Diary Card. Meanwhile, the study staff will make inquiries every week via telephone or in other ways. After *V3 + 30 days* (with a window period of +14 days) until the unblinding in the site, subjects are required to complete e-PRO, if applicable, and the study staff will make inquiries at least once every 4 weeks to record SAEs, MAAEs,

and AESIs. The window period for the above remote visits is ±3 days.

2) Safety monitoring theoretically related to the risk of VED: monitoring will be carried out during the follow-up period from the time the SARS-CoV-2 infection confirmed by RT-PCR occurs after at least one dose of vaccination until the end of the study. The specific possibility of enhanced respiratory disease is not yet clear, but it is theoretically related to the abnormal and excessive immunological type II reactions observed in animal studies of other coronavirus infections (e.g., SARS-CoV or MERS-CoV), although no such phenomenon has been observed in humans. Therefore, the study staff will monitor all severe cases to obtain relevant data, including but not limited to type of oxygen support needs (if applicable), organ system dysfunction, specific therapy, recovery time, and outcome (survival or death).

3) Within 7 days after each vaccination, if any solicited adverse event is the same as the suspected COVID-19 symptom, the investigator will further determine whether it is a suspected COVID-19 symptom. During the onset of a confirmed COVID-19 case, any symptoms or signs will not be recorded as an AE.

6.5 Laboratory Evaluation

6.5.1 Collection, transportation and storage of samples

Respiratory samples will be collected at the clinical trial site and sent to the corresponding laboratory for testing. Samples must be stored in a freezer with backup power supply and cold chain monitoring to ensure that they are properly stored. The procedures for obtaining and managing samples are documented in the laboratory manual.

The preparation, processing and storage of samples should be carried out in accordance with the laboratory manual. All samples will be labeled with subject ID number, sample number and barcode (or other identification code). The sample label must not contain any personally identifiable information. Both the shipping laboratory and the receiving laboratory will use the same sample preservation cold chain.

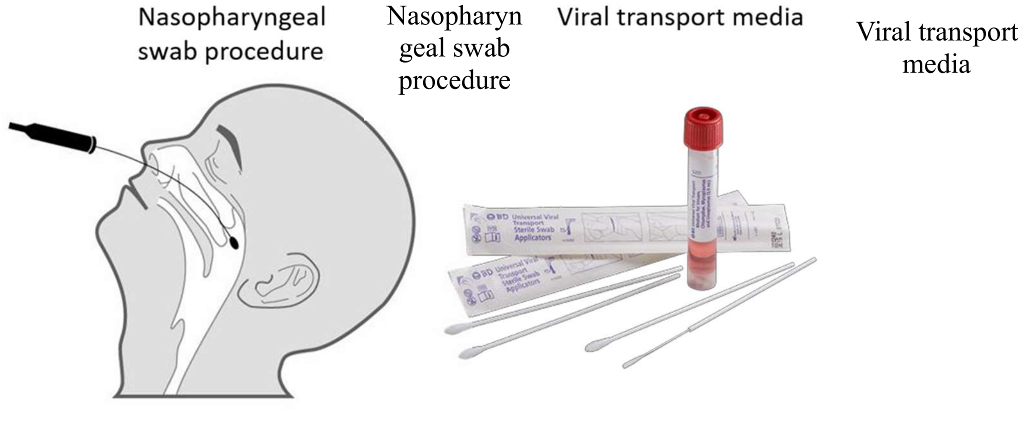

Figure 2 Schematic Diagram of Nasopharyngeal Swab Procedure and Viral Transport Media

6.5.2 Detection of samples

Nucleic acid detection (RT-PCR) of SARS-CoV-2 will be performed for all baseline-collected nasopharyngeal swabs (If nasopharyngeal swabs cannot be collected, oropharyngeal swabs should be collected, or additional oropharyngeal swabs can be collected in the same collection tube based on local policy. If nasopharyngeal swabs are not collected, please note the reason); the nucleic acid tests (RT-PCR) of SARS-CoV-2 and influenza A viruses will be performed for nasopharyngeal and/or oropharyngeal swabs collected during case monitoring period and follow-up period of confirmed cases; the sequencing of SARS-CoV-2 virus strains and influenza viruses (if applicable) will be performed for confirmed COVID-19 cases.

The samples will be detected in accordance with the laboratory manual.

6.5.3 Future use of preserved samples; correlation analysis

Samples will be stored for this purpose only when the subjects agree to the use of the samples for unscheduled purposes during the process of informed consent. Subjects can revoke their permission for other ways of using the samples in the future at any time. In this case, the samples will be destroyed after the study is completely over. The PI will ensure that all known remaining samples are destroyed and reported to the sponsor and the ERC/IRB. Other future uses of the sample will be reviewed and approved by the ERC/IRB (if applicable).

7 Safety Assessment and Reporting

Site investigators will perform safety monitoring on subjects to identify, assess and manage safety-related events. The project will set up a safety review team composed of the study site director, sponsor representatives, and the safety monitoring CRO team to conduct regular discussions on the safety of the study. The team may handle special situations related to enrollment, follow-up, and safety observation. The team will maintain communication so as to analyze emergent and suspected vaccine-related safety events in a timely manner.

Subjects will use a diary card/e-PRO to record the symptoms and signs of solicited adverse events that occur within 7 days after receiving each dose of the investigational vaccine, and record the unsolicited adverse events that occur after the first dose until 30 days after the last dose of vaccination. Subjects will be monitored for SAEs, MAAEs, and AESIs from the first dose to 1 year after the second dose.

All serious adverse events will be reported to the local Ethics Review Committee and regulatory authorities within the specified time as required.

7.1 Definitions Related to AE

7.1.1 Adverse events (AEs)

Adverse events are any uncomfortable or unexpected medical events that occur to the subject after receiving the study vaccines, including abnormal laboratory test results or diseases temporally related to the administration of study vaccines. Adverse events are not necessarily associated with the study vaccines. This definition includes the aggravation or worsening of previous symptoms after vaccination. A relatively stable previous symptom that has not

changed in severity during the study period will not be regarded as an adverse event, but this symptom should be recorded and reported as part of the previous medical history.

7.1.2 Adverse vaccine reactions (AVR)

Adverse vaccine reactions refer to any harmful or unanticipated reactions that may occur in clinical trials and be related to study vaccines. An AVR implies at least a reasonable possibility of a correlation between the study vaccines and an adverse reaction, i.e., relevance cannot be excluded.

7.1.3 Solicited adverse events

Solicited adverse events refer to pre-defined adverse events, that is, common or known symptoms or diseases related to vaccination, which are proactively monitored as potential indicators of vaccine reactogenicity. If a solicited symptom occurs during the solicitation period (7 days after each dose of vaccination), the investigator will assess the correlation of the solicited adverse event with the investigational vaccine.

In this study, the investigator will assess the occurrences of solicited adverse events through assessment at 30 minutes after each vaccination and 7 consecutive days of daily assessments (safety data collected from Day 0 to Day 7) of subjects. The investigator will provide subjects with diary cards or install e-PRO in their electronic devices to collect solicited adverse events and their severity as well as the use of concomitant medications.

The following solicited adverse events will be noted in this study: nasal obstruction, rhinorrhoea, sore throat, fever, cough, acute allergic reaction, headache, fatigue and asthenia, nausea, vomiting, diarrhea, myalgia, etc.

7.1.4 Unsolicited adverse events

Unsolicited AEs refer to other AEs than the specified solicited AEs, also including solicited AEs of the same term reported after the solicitation period, which are mainly any adverse events reported spontaneously by the subject, learned through inquiry during the study visit, or observed by the investigator, or discovered when consulting medical records or original documents.

7.1.5 Medically-attended adverse event (MAAE)

A medically-attended adverse event (MAAE) refers to an adverse event for which unconventional medical interventions (physical examination or vaccination) have been implemented, including hospitalization, an emergency room visit, or irregular medical treatment for any reason.

7.1.6 AEs of Special Interest (AESI)

Adverse events of special interest (AESI) refer to an AE (severe or non-severe) of special interest to the investigational vaccine that requires ongoing monitoring by the investigator and quick communication with the sponsor. The investigator should complete the report form within the same time as for reporting SAEs for quick reporting to the sponsor (or the CRO

designated by the sponsor). These events require further investigation to understand their characteristics. Depending on the nature of the event, the sponsor will also need to communicate quickly with other relevant parties, such as the regulatory authorities.

The listing of COVID-19 vaccine-related AESIs involved in this study is presented in table 2. This list is not intended to be exhaustive, nor does it exclude the possibility that other diagnoses may be AESI. It is expected that more AESIs may be related to COVID-19 vaccine, and the investigators should update relevant information at any time.

Table 2 The listings of AESIs related to COVID-19 vaccine*

**English name**	**Chinese name**
Acute respiratory distress syndrome	急性呼吸窘迫综合征
Multisystem inflammatory syndrome (children & adults)	多系统炎症综合征
Acute cardiovascular injury<br>*(includes: myocarditis/pericarditis, microangiopathy, heart failure, stress cardiomyopathy, coronary artery disease arrhythmia)*	急性心血管损伤*（包括：微血管病、心力衰竭、应激性心肌病、冠状动脉疾病、心律失常、心肌炎）*
Coagulation disorder<br>*(includes: thrombotic disorders, bleeding disorders)*	凝血障碍*（包括：血栓性疾病、出血性疾病）*
Anosmia, ageusia	嗅觉、味觉缺失症
Chilblain – like lesions	冻疮样病变
Erythema multiforme	多形性红斑
Single Organ Cutaneous Vasculitis	单器官皮肤血管炎
Acute kidney injury	急性肾损伤
Acute liver injury	急性肝损伤
Acute pancreatitis	急性胰腺炎
Rhabdomyolysis	横纹肌溶解
Subacute thyroiditis	亚急性甲状腺炎
Anaphylaxis	过敏反应
Thrombocytopenia	血小板减少症
Generalized convulsion	全身抽搐
Acute disseminated encephalomyelitis	急性播散性脑脊髓炎
Guillain Barré Syndrome	格林-巴利综合征
Acute aseptic arthritis	急性无菌性关节炎
Aseptic meningitis	无菌性脑膜炎
Encephalitis / Encephalomyelitis	脑炎/脑脊髓炎
Idiopathic Peripheral Facial Nerve Palsy	特发性外周面部神经麻痹
Vaccine associated enhanced disease	疫苗相关增强性疾病

*This listing is originated from Brighton Collaboration's Safety Platform for Emergency Vaccines (SPEAC).

7.1.7 Serious adverse event (SAE)

Serious adverse events are all adverse events with the following consequences:

- Resulting in death
- Life-threatening (life-threatening means that the investigator or the sponsor believes that the subject is in danger of death when the event occurs; it does not include the assumption that the event will cause death when it worsens)
- Requiring inpatient hospitalization or prolongation of existing hospitalization
- Permanent or severe disability or loss of normal life function
- Resulting in congenital abnormality or birth defects

Significant medical events that may not result in one of the above consequences but endanger the health of the subject and/or require medical or surgical intervention to prevent one of the above consequences are also considered as serious adverse events.

7.1.8 Suspected unexpected serious adverse reaction (SUSAR)

Suspected unexpected serious adverse reactions (SUSARs) refer to suspected and unanticipated serious adverse reactions whose clinical manifestations are beyond the Investigator's Brochure for the investigational vaccine, summary of product characteristics and other existing information in terms of their nature and severity.

7.2 Reporting Period and Parameters

The solicited adverse events will be assessed 30 minutes after vaccination, and in the next 7 days, the subjects' solicited AEs will be recorded daily. If a solicited AE that occurs within 7 days after vaccination lasts longer than 14 days after vaccination, it will continue to be reported as a solicited adverse event. Unsolicited adverse events will be collected after the first dose of vaccination until 30 days after the last dose of vaccination. SAEs/MAAEs/AESIs will be collected continuously from the day when the study vaccines are administered (Day 0) to 12 months after the second vaccination.

7.3 Severity of Adverse Events (AEs)

The severity of all adverse events will be assessed by the investigator and subject (if applicable). Adverse events will be graded according to the severity classification criteria from mild (grade 1) to life-threatening (grade 4) (see Appendix 2 for details). All adverse events leading to death are grade 5 events.

7.4 Correlation of Adverse Events (AEs)

The investigator will assess the correlation between the study vaccines and adverse events. The correlation assessment is based on the information obtained at the time of the report and can be changed later based on subsequent information. The correlation assessment is based on clinical judgment. The following factors should be considered:

- Is there a chronological relationship between the adverse event and the administration of

the study vaccines?

- Do the study vaccines have a reasonable biological mechanism to cause the adverse event?
- Are there other possible causes of the adverse event, such as concurrent disease or concomitant medication?
- Have there been reports of similar adverse events related to the study vaccines or other vaccines of the same category in the past?

In this study, the investigator must infer the correlation of adverse events based on the following definitions:

- Definitely related: The AE is a temporary result of immunization; and / or it is a known reaction mode of the investigational vaccine, which cannot be induced by the subject's clinical status, intervention therapy or concomitant treatment, but occurs immediately after immunization, or positive reaction develops at the vaccination site;
- Probably related: The AE is a temporary result of immunization; and / or it is a known reaction mode of the investigational vaccine, which cannot be induced by the subject's clinical status, intervention therapy or concomitant treatment;
- Possibly related: The AE is a temporary result of immunization; and / or it is a known reaction mode of the investigational vaccine, which may be induced by the subject's clinical status, intervention therapy and concomitant treatment;
- Possibly unrelated: The AE is most likely related to the subject's clinical status, intervention therapy and concomitant treatment; it is not a known reaction mode of the investigational vaccine;
- Unrelated: The AE is not related to vaccination, which is related to the subject's clinical status, intervention therapy and concomitant treatment.

Definitely related, probably related and possibly related are considered as related to administration of study vaccines, possibly unrelated and unrelated are considered as unrelated to the administration of study vaccines.

7.5 Follow-up of Adverse Events (AEs)

All adverse events reported during the study need to be followed up until the end, stability, subject death, loss to follow-up, or the end of the study. Medications for the treatment of AEs should be recorded in the subject’s source documents and the eCRF. Subjects who experienced serious adverse events related to the study vaccines after the completion of the study or the suspension of the study will be followed up by the principal investigator or designated personnel until the end of the event or the principal investigator confirms that it is irreversible, chronic or stable, or until the subject dies or is lost to follow-up.

The outcome of AEs will be assessed according to the following categories at the last observation:

- Recovered/ended (the adverse event has completely ended and has returned to baseline);

- Recovered/ended with sequelae (the adverse event has stabilized and not fully recovered to baseline, and there is no expected trend of further improvement);
- Recovering/resolving (the adverse event is ongoing and improving);
- Unrecovered/unresolved (the event is ongoing, but has not been improved, and there may be further changes);
- Fatal: if the adverse events are fatal, time of death should be recorded.
- Uncertain: the investigators are unable to understand the adverse events, e.g., loss to follow-up of subjects.

7.6 COVID-19-related AEs for VED Assessment

Based on previous studies of other viral vaccines and other coronavirus vaccines, the possibility of antibody dependent enhancement (ADE) or vaccine-enhanced disease (VED) cannot be ruled out with a SARS-CoV-2 vaccine. This will be evaluated by the difference in the severity of COVID-19 cases (especially severe cases) between the investigational vaccine and placebo.

7.7 General Principles for Recording of Adverse Events (AEs)

In order to improve the quality and accuracy of the adverse event data obtained, the principal investigator should follow the following guidelines:

- When recording adverse events on the Case Report Form, please use recognized medical terms as much as possible;
- If a disease diagnosis is known, the diagnosis (i.e., disease or syndrome) rather than symptoms should be recorded;
- Death is the outcome of an adverse event, and the event leading to death should be recorded and reported on the Serious Adverse Event Report Form;
- For patients who are hospitalized due to surgical procedures or medical procedures, the diseases that lead to the surgical procedures or medical procedures should be recorded as serious adverse events, rather than the diagnosis and treatment itself. Surgery and diagnosis and treatment procedures should be recorded in the event description as measures taken to deal with the disease;

7.8 Reporting of SAEs

The details and methods of the safety report will be provided in the safety management plan and will be briefly described in the following sections.

7.8.1 Investigator's responsibilities

For all SAEs that occur during the study period, regardless of whether they are related to the study vaccines, the investigator should complete, sign and date the SAE report form within 24 hours of knowledge, and immediately report it to the sponsor (or the CRO designed by the sponsor). All SAEs should be also recorded in eCRF forms. Information provided in SAE report forms must be consistent with event data documented in eCRF.

The investigator should report any SAE to the sponsor (or the CRO appointed by the sponsor) before its complete information is collected. Other relevant complete information, when available, can be supplemented subsequently. All the information known at the time of the report should be filled out in the SAE initial report form and include the minimum elements used for the initial assessment:

- The name and contact information of the investigator who submits the serious adverse event report;
- Subject identification number;
- The date when the subject receives the investigational vaccine;
- A description of the serious adverse event and the date when the event starts;
- The investigator's initial assessment of severity and correlation.

If applicable, hospital case records and autopsy reports (including oral autopsy) (without name or personal identification number) should be obtained.

The investigator will be responsible for notifying the Ethics Review Committee and regulatory authorities. The reporting procedures for all serious adverse events will be conducted in accordance with the current regulations and guidelines of specific countries/regions. The safety management plan will contain all detailed information of the regulatory report. Each report and document of the Ethics Review Committee, as well as a copy of the regulatory authority's notice and receipt will be kept in the study files.

7.8.2 Sponsor's responsibilities

Upon receiving the SAE report, the sponsor should immediately determine the "expectedness" and "correlation" of the SAE, so to identify whether it is a SUSAR and report in an expedited manner as required by the local regulatory. The sponsor should also report relevant safety information to the investigators/Ethics Review Committee in accordance with the local requirements.

7.9 Pregnancy-Related Events

The investigator will collect pregnancy-related events from the first dose to 12 months after the second dose, complete the pregnancy-related event report form after knowledge of the subject's pregnancy, and report it to the sponsor (or the CRO designed by the sponsor) via fax or email within 24 hours. Information of this pregnancy event will be collected on the pregnancy monitoring form. All pregnant women in the pregnancy-related event collection period will be followed up until end of pregnancy. The outcomes will be recorded, including the pregnancy outcome, delivery characteristics (duration of pregnancy, outcome, delivery), and conditions of the newborn (sex, weight, height, Apgar score). Pregnancy itself would not be considered as an SAE in this trial. Any complications during pregnancy will be considered as an AE and could be considered as an SAE in some cases such as spontaneous abortion, stillbirth, and congenital anomaly of the newborn.

7.10 Safety Monitoring

Although the principal investigator and/or designated staff are mainly responsible for monitoring the safety of all subjects and alerting the sponsor when unexpected problems occur, a more multi-layered safety monitoring system should be established, including the IDMC.

In this study, attention will be paid to acute respiratory distress syndrome, acute cardiovascular injury, thrombocytopenia and other adverse events that may cause serious consequences during the monitoring of AESI, which will be handled in accordance with the relevant requirements of the local risk control measures, diagnosis and treatment procedures.

7.11 Noncompliance with Regulations or Requirements

Any compliance verification/inspection of this study conducted by local or other government agencies, release of inspection reports, warning letters, or measures taken by regulatory authorities (including legal or medical measures, and serious or continuous violations of relevant laws and regulations) must be immediately reported to the sponsor.

8 Data Management and Data Retention

The investigator should ensure that the collected data are complete, clear, traceable, timely and accurate. The data collection will be carried out by the clinical trial site staff under the supervision of the site PI. All source files and laboratory reports should be reviewed by the clinical team and data entry staff to ensure the accuracy and completeness of the data. For AEs, the severity should be recorded in order to assess their seriousness and correlation, which should be reviewed and confirmed by site investigators.

The CRO is responsible for data management, including quality control, data analysis and research data reporting according to SOPs.

8.1 Data Management

The data management plan and the corresponding database should comply with the requirements of the International Conference on Harmonization of Technical Requirements for Registration of Pharmaceuticals for Human Use (ICH) and Title 21, Volume 11 of the US FDA Code of Federal Regulations, and will be carried out by the data management department of the CRO that the sponsor cooperates with. Data Management will establish, validate and maintain a GCP-compliant EDC system. The eCRF in the EDC system will be developed by Data Management, reviewed and approved by the project team and sponsor. The data system includes password protection and internal quality control, e.g., automatic range check, to identify inconsistent, incomplete or inaccurate data. Only authorized investigators or staff can access the system, and the EDC system will automatically save all entries and revision records in the eCRF.

Data Management will carry out all activities in accordance with its SOPs. The eCRF and any other supporting documents should be available for retrieval or review at any time.

8.1.1 Data coding

Previous medical history and adverse events will be coded by using the latest version of the Medical Dictionary for Regulatory Activities (MedDRA). The latest World Health Organization Drug Dictionary will be used for drug coding. The CRO's medical monitor will review and approve the code list.

8.1.2 Data validity

The CRO will check whether the data entered into the database is complete and consistent with the original data, including generating, tracking and resolving queries generated by the system's automatic verification and manual verification, as well as the differences between the two.

8.1.3 Source data verification

For source data verification, the monitor (on behalf of the sponsor) must be able to directly review the original documents, such as medical records, original laboratory records, and informed consent form (ICF). If the original data is electronic data, the data must be printed, signed and dated by the PI, and stored in the subject's study file. Important documents including ICFs must be always archived and kept in study files.

8.1.4 Relevant definitions

Source data: Source data include all information in original records and certified copies of original records, clinical findings, observations, or other activities in a clinical trial necessary for the reconstruction and evaluation of the trial. The original data is contained in the original documents (original records or certified copies).

Source documents: Source documents include original documents, data, and records (e.g., hospital records, clinical charts, laboratory notes, memos, participants' diary card or review checklists, certified accurate and complete copies or transcriptions, photographic films, participant files, and records kept at the laboratories and at medical technical department involved in the clinical trial).

8.2 Data Collection Method (Compilation and Completion of Case Report Forms)

The clinical data in the source documents will be directly entered into the EDC system by trained and qualified study staff. The data management system includes password protection and internal quality control, e.g., automatic range check, to identify inconsistent, incomplete or inaccurate data. The clinical data of each subject will be directly entered into the eCRF from the source document.

Site PIs are responsible for ensuring the accuracy, completeness and timeliness of the data reported in the subject's eCRF and any supporting documents. All source documents should be filled out in a neat and clear manner to ensure the accuracy of the data. The source documents supporting eCRF data should record the date and details of the study procedures, AEs, and subject status. The site PI/institution should securely store all the information in the eCRF and all source documents supporting the subject's data. Data and documents will be treated as

confidential materials.

8.3 Data Storage

All study documents (informed consent form and other documents that may associate the subject's personal information with the identification number) containing the subject's personal information will be stored securely with restricted access. These documents will only be provided to authorized personnel, including investigators and clinical personnel, who may need such information to treat subjects or for review reasons. In addition, representatives of the sponsor, regulatory authorities, and Ethics Review Committee can have access to the subjects' medical records to verify data.

8.4 Data Locking Procedure

After all subjects have completed all the visits and follow-up specified in the protocol, the seriousness of all AEs will be reviewed and finally determined. After all abnormal data have been resolved and all monitoring has been completed, the final database locking will be performed.

8.5 Study Record Retention

The PI is responsible for keeping study records for at least 5 years, starting from the day when the prequalification application is approved or rejected by the WHO. If no application is filed, the study records will be retained until 5 years after the completion of the clinical study report for the trial.

These records should also be kept in accordance with the local ERC and local medical record keeping requirements, subject to the specified maximum retention time. All documents related to the trial will be kept strictly confidential as required by local laws.

8.6 Protocol Deviation

A protocol deviation refers to any behavior that does not meet the requirements of the clinical trial protocol or GCP. Those who commit the non-compliance may be the subject, the investigator, or the staff of the site institution. If a protocol deviation occurs, the study institution should develop and immediately implement corrective actions. The study institution should remain vigilant to identify and report protocol deviations and deal with these deviations in accordance with corrective actions and preventive actions.

9 General Statistical Consideration

This section summarizes the general considerations for the statistical analysis of this study. The CRO will compile the SAP and conduct statistical analyses under the guidance and supervision of the sponsor.

The primary and secondary efficacy analyses will use Cox proportional hazard regression (the time when the SARS-CoV-2 infection virologically confirmed to be positive meets the clinical diagnostic criteria) for assessments, and be stratified according to country, COVID-19 vaccination (marketed or investigational) or not and age. The efficacy estimate is 1 minus the

estimated hazard ratio, and the confidence interval (CI) is converted accordingly. These hypothesis tests will be supplemented with the two-sided CI for the hazard ratios described below.

- Null hypothesis (H_0) and alternative hypothesis (H_1) for **interim analysis and final analysis** of virologically confirmed (RT-PCR-positive) COVID-19 case are as follows:
    - H_0: The protective efficacy of DelNS1-2019-nCoV-RBD-OPT1 against virologically confirmed (RT-PCR-positive) COVID-19 is ≤30%.
    - H_1: The protective efficacy of DelNS1-2019-nCoV-RBD-OPT1 against virologically confirmed (RT-PCR-positive) COVID-19 is >30%.

9.1 Sample Size

The sample size is dependent on whether sufficient primary endpoint cases could be observed. An interim analysis will be performed when the cumulative number of cases has reached 50% of the target number of primary endpoint cases (i.e., 75 cases). When enrollment is completed or 25% of the required total number of endpoint cases are accumulated, the project team will review the incidence of per protocol cases under the blind state and compare it with the expected value. If the actual incidence is lower than the expected incidence, the sample size may be appropriately increased. The project team will have the right to obtain the accumulation status of the endpoint events of trial sites under the blind state, so as to make a decision about starting or closing a site.

The following assumptions and thresholds are used to obtain the required sample size:

6-month infection rate (control group)	0.85%
Assumed efficacy of the vaccine	60%
Minimum efficacy required to be proven	30%
One-sided α	2.5%
Power	90%
Annual drop-out rate	20%
Interim analysis	1
Subject enrollment	Consistent enrollment date
Randomization ratio	1:1

This design has the following characteristics:

The number of events triggering the interim analysis	75
The number of events triggering the primary endpoint analysis	150
α spending value for interim analysis	0.0015
α spending value for primary endpoint analysis	0.0245

It is planned to enroll 32,000~40,000 subjects. The study population is grouped according to whether they have previously received COVID-19 vaccines: those who have not received any COVID-19 vaccine (marketed or investigational), those who have received at least one dose of

other COVID-19 vaccines (marketed or investigational) with an interval of ≥ 6 months between the last dose and the date when the subjects sign the informed consent for this study, with the number of subjects in each group accounting for about 50% of the total; moreover, subjects are also grouped according to the age of subjects: 18 ~ 59 years old and ≥ 60 years old. The proportion of the subjects aged 60 years and above in each study vaccine group is at least 20%, and the proportion of the Chinese/East Asians is not less than 20% of the total enrolled population. Such sample size is estimated based on all assumptions before the implementation of clinical trial. The actual sample size of enrollment may be adjusted according to the actual morbidity, vaccination rate, and the proportion of subjects that meet the protocol analysis conditions in each country.

The Lan-DeMets O'Brien-Fleming approximation spending function is used for alpha assignment so that the type I error is controlled within 2.5% in this study. In the interim analysis, if the number of events is different from the number of events listed above, the alpha will be adjusted based on the number of events by using Lan-DeMets O'Brien-Fleming approximation spending function.

9.2 Definition of Analysis Population

9.2.1 Enrolled population

The enrolled population is defined as all subjects who have signed and provided the informed consent form and are eligible to participate in the study after screening, regardless of the subjects' randomization and vaccination status in the study.

9.2.2 Safety analysis population

The safety analysis population is defined as all subjects who have been vaccinated in this study and have obtained any safety data. The safety summary will be analyzed based on the actual vaccine group.

9.2.3 Intent-to-treat (ITT) population

The ITT population is defined as all subjects who have been randomized.

Modified intent-to-treat (ITT) population 1 (mITT1)

The mITT1 population is defined as all subjects who are randomized and have received at least one dose of investigational vaccine or placebo. Subjects with vaccination errors will be evaluated for protective efficacy based on the randomized group according to the ITT principle. A second assessment will be performed in the mITT1 population for all efficacy endpoints after the first vaccination.

Modified intent-to-treat (ITT) population 2 (mITT2)

The mITT2 population is defined as all subjects who are randomized and have received full-schedule two doses of investigational vaccine or placebo. Subjects with vaccination errors will be evaluated for protective efficacy based on the randomized group according to the ITT principle. A second assessment will be performed in the mITT2 population for all efficacy

endpoints after the second vaccination.

9.2.4 Per-protocol (PP) population

The PP population is defined as:

- Those who correctly receive the study vaccines within the allowed window period during the entire process;
- Having no major protocol deviations.

Before database locking, potential deviations that need to be removed will be searched for in the database. In addition, protocol deviations will be collected from the monitoring and the medical checklist will be reviewed. The following are the general criteria (including but not limited to) for removal from the PP population:

- Vaccination severely beyond the window period;
- Non-compliance with the inclusion criteria or compliance with exclusion criteria
- Failure to store the vaccines received according to the conditions specified in the instructions;
- Unavailability of baseline virological or confirmatory test results of antibodies;
- Randomization error;
- Vaccination error

Subjects will be included in the PP population until the occurrence of major protocol deviations (e.g., administering other investigational COVID-19 vaccines). The data review meeting report will provide the criteria used to determine the analysis set, and list the subjects who are removed, as well as the time and reason for the removal.

The efficacy analysis will be performed mainly in the PP population.

9.3 Interim Analysis and Risk Monitoring

When the cumulative number of cases has reached 50% of the target number of primary endpoint cases (i.e., 75 cases) in the per-protocol efficacy analysis set, interim analysis will be initiated. The efficacy analysis will be mainly performed in per-protocol efficacy analysis set, modified intent-to-treat population 1 (mITT1) and modified intent-to-treat population 2 (mITT2). The analysis of the PP efficacy data set will be the basis for determining the efficacy of the vaccine. IDMC will review the interim analysis and will suggest terminating the study based on overwhelming confirmatory efficacy data, adequate ineffectiveness data or for safety consideration. Detailed information will be included in the interim analysis plan.

If the efficacy criteria have been met in interim analysis, conditional marketing approval can be applied for. If neither the efficacy nor the ineffectiveness criteria are met in the interim analysis, the trial will continue (if it has not been completed). In both cases, safety follow-up will continue even if the preliminary results are announced.

9.4 Placebo Cross-over Design

In each study site, when the last subject of the study site has been followed up for six months after the second dose (with a window period of +15 days), all the subjects of the study site should be unblinded (if the approval date by the local Ethical Review Committee is later than this date, the unblinding should be carried out within 2 months after the approval as far as possible) and cross-over of the placebo group will be conducted.

COVID-19 case monitoring will be ceased after the unblinding in the site, and SAE/MAAE/AESI will be collected via telephone follow-up or other communication methods at least once every 4 weeks. Subjects can also actively report SAE/MAAE/AESI through the completion of the study. The blinded subjects in other study sites will continue to be followed up for vaccine efficacy and safety as originally specified. Detailed information will be included in the statistical analysis plan.

9.5 Analysis Methods

9.5.1 Descriptive analysis

All collected data will be summarized and/or listed. The SAS software will be used for analysis.

Descriptive statistics generally include the mean, standard deviation, median, minimum, and maximum of continuous variables, as well as the number and proportion of categorical variables in each group. Statistical tests and confidence intervals will be calculated with a two-sided significance level of 5%. The exact CI will be used for univariate summary of dichotomic variables.

For the enrolled population, the medical history will be listed and summarized by category. Concomitant medications will be summarized by the anatomical therapeutic chemical classification, preferred drug name and vaccine grouping according to the WHO Drug Dictionary. Medical history will be summarized according to the MedDRA system organ class, preferred term and vaccine grouping.

The completion status of each link by all subjects will be summarized, including the number and percentage of registration, screening, randomization, and vaccination, as well as Consolidated Standards of Reporting Trials (CONSORT) chart describing study participation and suspension. The reasons for screening failures and withdrawals will be summarized and listed.

In addition to the summary and listing of vaccine management and sample collection, attendance at each visit will also be summarized and listed.

9.5.2 Change to analysis plan

Any deviation or change from the statistical analysis specified in the protocol will be described and explained in the SAP and clinical study report.

9.5.3 Analysis of baseline and demographic characteristics and subject compliance

Demographic characteristics (such as race, gender, age, baseline SARS-CoV-2 antibody test

results, etc.) will be descriptively summarized in the safety analysis data set, modified intent-to-treat population 1 (mITT1) and modified intent-to-treat population 2 (mITT2), per protocol set, and grouping populations based on other baseline characteristics (such as medical history, concomitant diseases, etc.). According to age group (18-59 years, ≥60 years), chi-square test/Fisher's exact test is used for binary variables, and t test or analysis of variance is used for continuous variables.

The subject compliance analysis includes a descriptive summary of dropouts and reasons for the dropouts.

9.5.4 Analysis of primary endpoints

The analysis of the primary endpoint in this trial is based on Stage 1 study, that is, the study phase before placebo crossover.

The results are obtained from nasopharyngeal and/or oropharyngeal swab samples at any time during the case monitoring period of the trial. After the judgment, if it is virologically confirmed (RT-PCR-positive) and accompanied by symptoms consistent with COVID-19, the definition of endpoint case events will be met.

Cox proportional risk regression will be used to assess the efficacy of the vaccine against virologically confirmed symptomatic (RT-PCR-positive) COVID-19 of any severity at least 14 days (⩾15 days) after the second vaccination, with stratification by country, previous COVID-19 vaccination (marketed or investigational) or not and age. VE estimate (1-hazard ratio), 95% CI and *p* value will be calculated with the model. This analysis will be performed in the PP and mITT2 population.

In the PP population, the final hypothesis test for the primary efficacy endpoint will be based on the alpha level and the corresponding CI used in the analysis. Final efficacy analysis will also be performed based on the mITT2 population.

9.5.5 Analysis of secondary endpoints

The assessment method of the secondary efficacy endpoint is the same as that of the primary efficacy endpoint.

The secondary efficacy analysis will be performed mainly in the per protocol population, and repeated in the mITT1 or mITT2 population.

9.6 Safety Analysis

Safety analyses will be performed in the safety analysis data set according to the vaccine received, including overall analysis, age grouping and other subgroup analysis.

(1) Solicited AEs

Solicited events will be summarized and analyzed by calculating the proportion of subjects experiencing any adverse event in the vaccine group and the placebo group (which will be classified according to severity and event type). The analysis will include adverse events within 30 minutes after vaccination and self-recorded adverse events within 7 days. In addition, the

incidence and duration of adverse events that last more than 7 days will be analyzed. The adverse events with the highest severity and longest duration will be summarized and analyzed for each subject. For the incidence of solicited adverse events, the two-sided 95% confidence interval will also be calculated. The two-sided chi-square test/Fisher's exact test of pairwise comparison will be used for the incidence of solicited adverse events, including the overall and inter-layer tests. For serious adverse events, the test and analysis will be repeated.

(2) Unsolicited AEs, SAEs, MAAEs and AESIs

All unsolicited AEs, SAEs, MAAEs and AESIs will be coded, tabulated and summarized. Unless an AE is classified as an SAE, only the records of adverse events that occur within 30 days after vaccination will be used to summarize and analyze unsolicited AEs. All unsolicited AEs will be listed in the appendix, regardless of their severity and onset time. Unsolicited AEs will be summarized mainly at the subject level, that is, for a given adverse event, the subject will be included in the analysis with the highest severity and/or correlation.

Unsolicited AEs will be analyzed for their severity and their correlation to the vaccine; SAEs will be analyzed based on their correlation to the vaccine. In addition, all AEs will be coded using MedDRA and summarized by classification, System Organ Class (SOC), and Preferred Term (PT). The attachment will include the incidence of AEs in subjects who have withdrawn from the trial. A list of all SAEs and related AEs will be presented. Within the age group, a two-sided chi-square test/Fisher's exact test will be used to compare the proportion of subjects with AEs of grade 3 and above, vaccine-related AEs, and SAEs.

(3) Vaccine-enhanced disease (VED)

The likelihood of vaccine-enhanced disease (VED) will be assessed through the severity of adverse respiratory events in different groups (especially COVID-19 cases) and the incidence of serious respiratory adverse events (especially COVID-19 cases) in each group. The severity score will be analyzed by Wilcoxon rank sum test. The proportion of serious and non-serious events in the overall events will be compared. A separate CRF will be used to collect the symptoms and severity of respiratory diseases, and the distribution of the severity in the overall population and different age subgroups will be analyzed.

9.7 Processing of Dropouts and Missing Values

All missing data will be assumed to be missing completely at random, and no value will be assigned. Therefore, subjects who have missing data or cannot be assessed will be excluded from the analysis. If too many data are missing, or a pattern of the missing data has been detected, the SAP can supplement and provide corresponding analysis methods to solve this problem.

10 Quality Assurance and Quality Control

Internal and external processes are guided to ensure effective protocol implementation, study quality, and compliance with the sponsor's and applicable regulatory requirements.

10.1 General Principles

This study will be carried out in accordance with the protocol and ICH GCP to assure the public that the rights, safety and benefits of subjects are protected and that clinical trial data are reliable. In order to ensure quality and standardization, the study will be carried out in accordance with the protocol content or other written guidelines. Regular operational site inspections will also be carried out to verify that all links are carried out correctly and completely. Before the start of the study, the CRO will offer protocol training to study staff, including training on applicable standard operating procedures.

The study site will provide all study-related documents, original data/documents and reports for monitoring by the sponsor and inspections by local/regulatory authorities.

10.2 Clinical Monitoring

The CRO will, on behalf of the sponsor in this study, be responsible for ensuring that the study is carried out in accordance with ICH GCP and related regulations. To this end, the monitor will provide external monitoring for this study, conduct site inspections before the start of the study, and conduct monitoring at the beginning, during and at the end of the study. During the study process, the monitor will regularly have access to the trial site/data management (network virtual access is allowed) to confirm the compliance with the protocol, the integrity, accuracy and consistency of the data and study vaccines, and compliance with ICH GCP and related regulations. According to the actual situation, the monitor can also provide explanations and conduct additional training on the trial site to help site solve the problems found in monitoring process. Under appropriate conditions and with informed risk assessment, remote centralized monitoring can be considered to replace or supplement site monitoring. The content of monitoring may include data quality analysis (e.g., missing or inconsistent data, abnormal data, etc.) and data trends that are difficult to find through site monitoring and various indicators (e.g., screening failure rate and withdrawal rate, subjects' non-compliance with inclusion criteria/compliance with exclusion criteria, timeliness and accuracy of data submission).

The scope and frequency of monitoring visits will be described in the clinical monitoring plan developed before the start of the study. The investigator will be notified before any planned monitoring visit. The monitor should have the right to review the trial site, the medical records of the subjects, the accountability system for study vaccines, and other study-related records required to carry out the monitoring work. The CRO will inform the site PI of the monitoring results and problems found, including any corrective actions. The site PI and the monitor must agree to cooperate to ensure that any problems found during the monitoring can be resolved within the specified time.

10.3 Third Party Audits

The sponsor or its designated institution may conduct audits on this study to ensure that the trial procedures and collected data comply with the protocol requirements and related SOPs, and that the data are correct and complete. The PI will allow inspection and verification of source data of the clinical study for regular monitoring. The auditor will compare the entries in

the eCRFs with the original data, and assess whether the study site complies with the clinical study protocol, GCP guidelines, and applicable regulatory requirements.

10.4 Inspection by Management Agencies

The PI must be aware that regulatory authorities including ERC/IRB may conduct site inspections to verify the validity and integrity of study data and the protection of human study subjects. The PI should notify the CRO within 24 hours after contacting the regulatory authorities. The PI must provide relevant records for inspection which can be used to respond to reasonable request and verification from authorized representatives of regulatory authorities.

10.5 Endpoint Adjudication Committees (EAC)

The EAC will consist of specialists in infectious diseases, internal medicine, or respiratory medicine to review the data related to cases that are positive for RT-PCR test performed by the central laboratory, so as to adjudicate confirmed cases of COVID-19. The EAC will review and assess the severity of confirmed COVID-19 cases according to severity grading criteria issued by the WHO and NMPA (see Appendices 4 and 5).

10.6 Independent Data Monitoring Committee (IDMC)

An Independent Data Monitoring Committee (IDMC) composed of independent vaccinology experts, infectious disease experts and biostatisticians will be established to review accumulated data on a regular basis. The responsibilities and working procedures of the IDMC will be defined in the IDMC charter.

The IDMC will be responsible for safeguarding the rights and interests of trial subjects, assessing safety during the trial period, and monitoring the overall progress of the clinical trial. The IDMC will provide the sponsor with suggestions on continuing, modifying or stopping the trial. The items reviewed by the IDMC include cumulative and demographic information of study subjects, interim/final safety data, suspension of inoculation of investigational vaccines, factors that may affect the study results or destroy the blindness of the trial data, data quality, integrity, and timeliness, and factors other than the interim efficacy/ineffectiveness analysis study, such as scientific or therapeutic progress that may affect the safety of subjects or study ethics.

The IDMC will hold a meeting before the start of the study. In addition to regular general meetings, if serious safety hazards are found, the IDMC will hold a conference call to jointly review the data. The IDMC review will summarize the information and suggest to the sponsor whether the study should be continued, modified or terminated.

11 Ethical Considerations (and Informed Consent)

11.1 Ethical Standards

This study will follow ICH GCP, the Declaration of Helsinki and all applicable laws and regulations. The study protocol, informed consent forms for different study sites, subject training and recruitment materials, as well as other required documents (including any subsequent revisions) should be reviewed and approved by the Ethical Review Committee

(responsible for supervision of the study conducted at the study site). After preliminary review and approval, the Ethical Review Committee should review the study at least once a year.

11.2 Ethical Review

The principal investigator is responsible for ensuring that the protocol, informed consent form and other relevant documents are approved by the local Ethical Review Committee before the start of the study. Any revisions made to the protocol, informed consent form or other relevant documents must be approved by the Ethical Review Committee. The protocol, samples of the informed consent form or written information of other subjects and copies of any samples of recruitment materials should be submitted to the Ethical Review Committee for written approval. The investigator should submit all subsequent revisions to the protocol or informed consent form to the Ethical Review Committee to obtain approval when necessary. The investigator should report the occurrence of SAEs and protocol deviations to the Ethical Review Committee in accordance with the requirements of the local regulatory authority and the Ethical Review Committee. The study should be carried out in full accordance with the protocol.

Without the written approval of the sponsor, the content of the protocol may not be modified. All revisions to the protocol must be submitted to the relevant Ethical Review Committee for approval before the modified protocol is implemented at each study site.

11.3 Informed Consent Process

Before any procedures or interventions stipulated in the protocol are conducted, the principle of informed consent in the Declaration of Helsinki (current version) should be followed. The informed consent process should begin before an individual agrees to participate in the study and continue throughout the entire process of participating in the study. Before conducting any study-related procedures, the investigator should ensure that the subject fully understands the objectives, procedures, potential risks and benefits of the study. Subjects should obtain the written informed consent form and have sufficient time to read and understand the content. Subjects will be encouraged to raise relevant questions of this study and get detailed answers, and have enough time to consider whether to participate in this study or not. Subjects should voluntarily participate in the study, and have the right to decide whether to refuse to participate in or withdraw from the study at any time during the trial.

Before conducting any study-related procedures, the investigator must obtain an informed consent form voluntarily signed and dated by the subject. The investigator must record the informed consent process. The original signed informed consent form should be kept in the site study file. The subject should obtain a copy of the informed consent form. Informed consent-related records should be kept in the original documents of the subject.

11.4 Confidentiality

The investigator, sponsor, and all staff involved in the trial must ensure that the subjects' information is kept confidential. No personally identifiable information is included in any study-related reports. All study records should be kept confidential within the limits prescribed

by national and local laws. For the sake of protection of subjects, the representative of the sponsor may provide medical records containing identification information for review. In addition, regulatory authorities may require verification of study records when reviewing and approving vaccines for marketing. The verification content may include inspecting, analyzing, verifying and copying any records and reports that are critical to the assessment and study. The study results may be published in medical books or journals, or used for academic conference exchanges, etc., but no personally identifiable information about the subjects will be listed.

Under appropriate conditions, the study procedures will protect the privacy and confidentiality of the subjects as much as possible.

All study-related information should be kept securely at the study site. All subjects' information should be stored in a locked file storage room, and only designated investigators have permission to enter. In order to protect the privacy of subjects, data collection, study procedures, related management forms, laboratory samples and other reports should use ID to identify subjects. All records containing names or other personal identifications, such as informed consent form, should be stored separately from other study documents identified by ID numbers. All local databases should adopt a password-protected access system. Forms, lists, work logs, and any other files that associate subject ID numbers with other identification information should be stored in a separately locked folder with restricted access. Without the written permission of subjects, their study information should not be published, unless for monitoring purposes.

11.5 Compensation

Generally speaking, unless there are relevant guidelines for a specific country, no compensation will be made for the time consumed by the subject during the study period, lost wages, or any inconvenience. For diseases diagnosed during the study that are not related to the study vaccines, the trial team will not pay the cost of long-term treatment.

11.6 Risks and Benefits

Subjects may benefit from the protection of this candidate vaccine (DelNS1-2019-nCoV-RBD-OPT1). However, this candidate vaccine may also pose a risk to subjects infected with SARS-CoV-2 after vaccination or cause aggravation of COVID-19. It is hoped that participating in this study will help apply the COVID-19 vaccine more widely to solve global public health problems.

Allergic reactions may occur after vaccination (including marketed vaccines). In rare cases, these reactions may be life-threatening. The investigator should be informed of this possibility through the protocol and Investigator's Brochure. Through the informed consent form, all subjects should be informed that they should be observed for at least 30 minutes after receiving the study vaccines. As with all immunizations, if there is a severe immediate reaction (such as anaphylactic shock), appropriate emergency medical measures are allowed. Subjects known to be allergic to any ingredient of the investigational vaccine should be excluded.

The risks associated with blood draws and venipuncture may include minor bleeding or

bruising at the venous access, minor discomfort, stomach discomfort, dizziness and vertigo, fainting, or a very small number of infections. All staff using aseptic technique to take blood samples should have been trained. If any complication occurs, the investigator will provide medical assistance. Subjects should be informed of the risk of blood draws during the informed consent process, and take a sitting or supine position during blood draws.

The risks associated with nasopharyngeal/oropharyngeal swabs are low. After swab collection, some subjects may experience short-term coughing and sneezing, and a few subjects may experience irritation or slight bleeding in the nasal cavity.

VED is a theoretical risk (see Section 1.5.2). During the informed consent process, subjects must be reminded that the vaccine has a potential risk of inducing respiratory diseases with SARS-CoV-2 infections that are more severe than expected.

11.6.1 Risks Faced by Investigators

The main risk faced by investigators is that they may be infected with blood-borne pathogens (including HBV, HCV, and HIV) when handling medical wastes that may be contaminated by blood, body fluids, nasopharyngeal and/or oropharyngeal secretions, and during blood collection. Compliance with the standard operating procedures when exposed to infectious pathogens and the adoption of general precautions will reduce the risk of exposure.

Study staff may have direct contact with participants infected with COVID-19. Study staff will receive special training and take proper personal protection on site to fully prevent and control the risk of COVID-19 infection.

11.7 Reporting of Infectious Diseases

The reporting will be completed in accordance with the relevant requirements of the trial site.

11.8 Study-related Injury Treatment and Compensation Methods

Subjects with AEs during the study period should be assessed and treated by clinicians of the study institution as far as possible. When the study doctor is unable to provide appropriate assessment and medical intervention, the subjects should be referred to other medical institutions based on the characteristics of the chief complaint. Any costs of treatment of vaccine-related AEs in the study in accordance with local good medical practices will be borne by the trial team.

According to legal requirements, the sponsor and the vaccine manufacturer should purchase study-induced injury insurance for subjects, and clinical trial insurance will be used to pay for study-related injury treatment. If any injury occurs, relevant compensation (including expenses for long-term and future medical needs) should be provided. Subjects should be informed that accepting compensation is not a waiver of their legal rights.

12 Publication and data sharing policy

The information generated in this study will be used by the sponsor in the work related to product development. Therefore, reports may be published to various government regulatory

authorities and global public health organizations (such as WHO). The study results will be announced to the public in accordance with the timetable for the publication of clinical trial results prescribed by the WHO. The member journals of the International Committee of Medical Journal Editors have adopted the clinical trial registration policy as a condition for publication. The sponsor or its designee will prepare a clinical study report in accordance with the ICH-E3 guidelines.

While publishing study results in well-known scientific journals or seminars or conferences, R&D institutions should protect the integrity of ongoing trials. The publications, lectures or manuscripts of any study results of any individual participating in the study will be governed by the procedures prescribed by the relevant parties in the clinical trial protocol. In any presentation or publication, the subject’s personal information will be kept confidential, and subject numbers and initials will be used to identify subjects if necessary.

Any new information in this study that may affect the safety, benefit, or willingness to continue participating in this study will be communicated to subjects. If requested by the subject, the study results will be notified thereto, and the information will be explained in detail through a notification letter approved by the Ethics Review Committee.

Appendix 1 Visit Schedule

Visit Schedule

**Study Phase**	**Screening stage[1]**	**Vaccination**		**Follow-up period (30 days after the second vaccination)**	**Until the unblinding in the site**
**Visit time**	**D-7~D-1**	**D0[1]**	**D14 （+14d）**	**V3+30d （+14d）**	**Follow-up during the study**
**Visit**	**1**	**2**	**3**	**4**	**-**
Site visit	X	X	X	X	
Regular telephone/short message/email follow-up					X
Informed consent[2]	X				
Height and weight	X				
Physical examination[3]	X	X	X	X	
Vital signs[4]	X	X	X	X	
Review of inclusion/exclusion criteria	X	X	X		
Collecting demographic and contact information[5]	X				
Medical history, treatment history, medication history, and vaccination history [6]	X				
RT-PCR test for SARS-CoV-2[7]	X	To be done as indicated			
Detection of SARS-CoV-2 specific antibody (fingertip blood)[8]	X				
Randomization		X			
Laboratory test[9]	clinically indicated at the investigator’s discretion				
Collecting information of concomitant medications.	X	X	X	X	X
Pregnancy test[11]	X	X[11]	X		
HIV screening[12]	X				
Vaccination[13]		X	X		
Site observation for 30min following vaccination		X	X		
Distribution/recovery of subjects’ diary card[14]		X	X	X	
Dispensing thermometer[15]		X			
Adverse events[16]		X	X	X	
Protective efficacy follow-up[17]		From after the first vaccination to			After V3+30

		V3+30d (+14 days), the subject will complete e-PRO or Diary Card daily, and the study staff will inquire them every week.	days (with a window period of +14 days), the subject will complete e-PRO, if applicable, and the study staff will inquire them every week.
Collecting SAEs/MAAEs/AESIs[18]		From after the first vaccination to V3+30d (+14 days), the subject will complete e-PRO or Diary Card daily, and the study staff will inquire them every week.	After V3+30 days (with a window period of +14 days), the subject will complete e-PRO, if applicable, and the study staff will inquire them at least once every 4 weeks.

Notes:

1. If the screening period and the first dose occur on the same day, the two visits can be combined into one visit.
2. Written informed consent should be obtained prior to any study-related examinations or procedures.
3. Physical examination: a comprehensive physical examination will be performed at screening, including general conditions (including weight and height), head and neck, lymph nodes, skin, chest, abdomen, and musculoskeletal system, etc.; only a simple physical examination will be required for other visits, including but not limited to general conditions, skin, and any abnormal signs that, at the investigator's discretion, require attention.
4. Vital signs examination includes blood pressure and body temperature. Body temperature measurement such as axillary temperature should be accurate to 0.1℃.
5. Demographics include age, sex, race and ethnicity. Each subject will also need to provide their current contact information including telephone number and/or email; any changes to the demographic information of the subjects should be updated at V3 and V4.
6. Obtain subjects' medical history, treatment history and prior medication information by inquiry and/or reviewing the medical records, and record subjects' health status; the medical history and health status of subjects should be updated at V3 and V4 if necessary; for subjects with vaccination history of COVID-19 vaccines (marketed or investigational) obtained by inquiry, it is necessary to provide the relevant supporting documents for the vaccination and record in detail the time of vaccination, manufacturer and dose of the vaccine.
7. The nasopharyngeal swab should be collected at screening whenever possible for RT-PCR test for SARS-CoV-2 by the study institution of the country where the study is conducted. During the trial, for subjects meeting the criteria for triggering SARS-CoV-2 nucleic acid test, their nasopharyngeal and/or oropharyngeal swabs will receive RT-PCR testing for SARS-CoV-2. RT-PCR re-testing for SARS-CoV-

2 and influenza virus will be performed by the central laboratory.

8. Fingertip blood is collected from the subjects for the detection of SARS-CoV-2 specific antibody, and the results are recorded.
9. As clinically indicated, the investigator will decide whether to perform the laboratory tests including blood routine, blood biochemistry, urine routine, and coagulation function, etc.
10. Record any medications that the subject has used from signing ICF to 30 days after the second dose; beyond 30 days after the second dose, treatments for SAEs/MAAEs/AESIs, if any, should also be recorded.
11. Pregnancy test: required only for women of childbearing potential. Urine or blood pregnancy test can be performed as appropriate for the site. For blood pregnancy test, a result obtained within 7 days before vaccination is acceptable; for urine pregnancy, only the result obtained on the day of test is acceptable. On the day of the first dose vaccination, pregnancy test can be determined according to the actual testing conditions of the study institution to facilitate operation. In this protocol, women of childbearing age are defined as sexually mature women: 1) had not undergone hysterectomy or bilateral oophorectomy; 2) (⩾45 years of age) had not experienced spontaneous menopause (without medication or other influencing factors) for 12 consecutive months.
12. It will be done depending on the relevant policy of the country where the trial is conduct, if qualification for HIV testing is required in the country, this information will be obtained mainly by inquiry while protecting the candidate's privacy.
13. Before vaccination, clean the vaccination site (both nasal cavities) with a wet cotton swab and let it dry.
14. The subject will receive the Subject Diary Card (electronic and/or paper) after each vaccination. Solicited AEs reported 0-7 days following each vaccination and unsolicited AEs reported within 30 days following the first and last doses will be recorded. The investigator will collect the previously dispensed Diary Cards and dispense new Diary Cards at site visits. At the last site visit (visit 4), all Diary Cards will be collected and no new card will be dispensed.
15. Thermometer will be dispensed after the first inoculation.
16. Adverse events should be recorded after the first vaccination of subjects. Solicited AEs should be collected from 0 to 7 days after each vaccination as well as unsolicited AEs from the first vaccination to 30 days after the last vaccination.
17. Through an e-PRO installed on a mobile phone or other electronic device or diary card, the subject will record/report any symptoms that may be related to COVID-19 from after the first vaccination to *V3 +30 days* (with a window period of +14 days) on a daily basis, and the study staff will weekly make inquiries via telephone or other communication methods. After *V3 +30 days* (with a window period of +14 days) until the unblinding in the site, subjects are required to complete e-PRO, if applicable, and the study staff will make inquiries every week. The window period for the above remote visits is ±3 days.
18. Subjects will record/report whether they have experienced any AEs including SAE/MAAE/AESI daily after the first vaccination to *V3 +30 days* (with a window period of +14 days) using e-PRO installed on their mobile phone or other electronic devices or the Diary Card. Meanwhile, the study staff will make inquiries every week via telephone or in other ways. After *V3 +30 days* (with a window period of +14 days) until the unblinding in the site, subjects are required to complete e-PRO, if applicable, and the study staff will make inquiries at least once every 4 weeks to record SAEs, MAAEs, and AESIs. The window period for the above remote visits is ±3 days.

Appendix 2 Table of severity grading of adverse events

Severity of AEs will be determined based on NMPA *Guidelines for the Grading Criteria of Adverse Events in Clinical Trials of Prophylactic Vaccines*. The severity grading is shown in the table below:

Appendix 2-Table 1 Grading of AEs

Symptoms/ Signs	Grade 1	Grade 2	Grade 3	Grade 4
Pyrexia* [axillary temperature (°C)]	37.3~<38.0	38.0~<38.5	38.5~<39.5	⩾39.5, persisting over 3 days
Diarrhea	Mild or transient, 3~4 stools/day, abnormal stool appearance, or mild diarrhea persisting for less than 1 week	Moderate or persistent, 5~7 stools/day, abnormal stool appearance, or diarrhea >1 week	>7 stools/day, abnormal stool appearance, or hemorrhagic diarrhea, orthostatic hypotension, electrolyte imbalance, requiring intravenous infusion >2L	Hypotensive shock, requiring hospitalization
Constipation**	Requiring stool softener and diet adjustment	Requiring laxatives	Stubborn constipation requiring manual dredge or use of enema	Toxic megacolon or intestinal obstruction
Dysphagia	Mild discomfort during swallowing	Diet is restricted	Diet and conversation are severely restricted; cannot eat solid food	Cannot eat fluid food; requiring parenteral nutrition
Anorexia	Decreased appetite, but food intake is not reduced	Decreased appetite, food intake reduced but no significant loss of weight	Decreased appetite and significant loss of weight	Requiring intervention measures (e.g., tube feeding, parenteral nutrition)
Vomiting	1~2 times/24h and not limiting activity	3~5 times/24h or limiting activity	>6 times/24h or requiring intravenous rehydration	Require hospitalization or nutrition obtained from other routes due to hypotensive shock
Nausea	Transient (<24 hours) or intermittent, food intake generally normal.	Persistent nausea leading to reduced food intake (24 to 48 hours).	Persistent nausea leading to almost no food intake (>48 hours) or requiring intravenous rehydration.	Threatening life (e.g., hypotensive shock)
Myalgia	Not limiting ADL	Slightly limiting ADL	Severe muscle pain, severely limiting ADL	Emergency treatment or hospitalization

Arthritis	Mild pain with inflammation, erythema or joint swelling causing no interference with function	Moderate pain with inflammation, erythema or joint swelling impairing function but not limiting ADL	Severe pain with inflammation, erythema or swelling of the joint limiting ADL	Permanent and/or disabling joint injury
Arthralgia	Mild pain, not interfering with functioning	Moderate pain, requiring analgesics and/or pain interfering with functioning, but not limiting ADL	Severe pain, requiring analgesics and/or pain limiting ADL	Disabling pain
Headache	Not limiting ADL, not requiring treatment	Transient, slightly limiting ADL, may require treatment or intervention	Severely limiting ADL, requiring treatment or intervention	Refractory, requiring emergency treatment or hospitalization
Syncope	Near syncope without loss of consciousness (e.g., pre-syncope)	Loss of consciousness with no intervention indicated	Loss of consciousness AND Hospitalization or intervention required	NA
New Onset Seizure	NA	NA	1 to 3 seizures	Prolonged and repetitive seizures (e.g., status epilepticus) OR Difficult to control (e.g., refractory epilepsy)
Cough	Transient, no treatment is required	Persistent cough, treatment is effective	Paroxysmal cough, which cannot be controlled through treatment	Need for emergency treatment or hospitalization
Acute Bronchospasm	Transient, no treatment is required; FEV1% of 70% to 80%	Treatment required; normalization by bronchodilator therapy; FEV1% of 50% to 70%	Failure to return to normal with bronchodilator therapy; FEV1% of 25% to 50%, OR persistent intercostal depression	Cyanosis; FEV1% <25%; OR need for intubation
Dyspnea	Dyspnea during exercise	Dyspnea during normal activities	Dyspnea at rest	Dyspnea, requiring oxygen therapy, hospitalization or assisted respiration
Pruritus (without skin lesions)	Mild itching, not limiting or slightly limiting	Itching leading to limiting ADL	Itching leading to incapability to perform ADL	NA

	ADL			
Abnormal cutaneous mucosa	Erythema/pruritus/color changed	Diffuse skin rash/maculo-papular rash/dryness/desquamation	Pustuliform/exudation/desquamation/ulcers	Exfoliative dermatitis involving mucous membrane, or erythema multiforme or suspected Stevens-Johnsons syndrome
Insomnia**	Mild difficulty in falling asleep, causing no or minimal interference with ADL	Moderate difficulty in falling asleep, causing more than minimal interference with ADL	Severe difficulty in falling asleep, causing inability to perform ADL, requiring intervention or hospitalization	NA
Agitation or inhibition	Mild agitation or mild inhibition	Agitation or drowsiness	Inability to soothe or low reaction	NA
Psychiatric disorders (including anxiety, depression, mania, and psychosis) Specify disorders	Mild symptoms with intervention not indicated OR behavior causing no or minimal interference with ADL	Symptoms with intervention indicated OR behavior causing greater than minimal interference with ADL	Symptoms with hospitalization indicated OR behavior causing inability to perform ADL	Threatens harm to self or others OR acute psychosis OR behavior causing inability to perform basic self-care ability
Acute allergic reactions&	Localized urticaria (wheals) with no medical intervention indicated	Localized urticaria with intervention indicated, OR mild angioedema with no intervention indicated	Generalized urticaria or angioedema with intervention indicated, or symptoms of mild bronchospasm	Anaphylactic shock or life-threatening bronchospasm or laryngeal edema
Fatigue/asthenia	Not limiting ADL	Limiting ADL	Severely limiting ADL, inability to work	Emergency treatment or hospitalization
Pain# (the site will be specified during reporting)	Mild pain, not limiting or slightly limiting ADL	Pain leading to limiting ADL	Pain leading to incapability to perform ADL	Disability pain, incapability of self-care

Note: *axillary temperature is generally used in China, oral temperature and anal temperature should be used if necessary. Generally, ear temperature = oral temperature = axillary temperature + 0.2°C; anal temperature=axillary temperature+ (0.3-0.5°C). When persistent hyperpyrexia occurs, the cause of hyperpyrexia should be identified as soon as possible.

**Attention should be paid to changes before and after vaccination for constipation and insomnia.

& refers to Type I hypersensitivity.

# refers to pain other than myalgia, arthralgia, and headache.

The adverse events which are not included in the above grading table will be assessed according to the following severity criteria.

Appendix 2-Table 2 General Rules for Severity Grading of Other Adverse Events

Grade 1	Grade 2	Grade 3	Grade 4	Grade 5
**Mild:** short term (<48h) or slight discomfort, not limiting activity, not requiring treatment	**Moderate:** mildly or moderately limiting activity, may require hospital visit, not requiring or only requiring mild treatment	**Severe:** significantly limiting activity, requiring hospital visit and treatment, may require hospitalization	**Critical:** may be life-threatening, severely limiting activity, requiring intensive care	Death

Appendix 3 Placebo Cross-over Design

In each study site, when the last subject of the study site has been followed up for six months after the second dose (with a window period of +15 days), all the subjects of the study site should be unblinded (if the approval date by the local Ethical Review Committee is later than this date, the unblinding should be carried out within 2 months after the approval as far as possible) and cross-over of the placebo group will be conducted. That is, subjects in the placebo group could choose to cross over to receive the investigational vaccine (DelNS1-2019-nCoV-RBD-OPT1), or they could choose to discontinue vaccination with the investigational vaccine and withdraw from the study, and such subjects would be considered to have completed the study.

The cross-over design is as follows:

After the trial meets the condition for cross-over, subjects in the placebo group who are willing to cross over to the investigational vaccine will be unblinded by designated independent personnel, and will be notified by the investigator to perform the vaccination visits.

➢ **First vaccination visit**:

After unblinding, the investigator will arrange the visits as early as possible (the specific visit time is to be determined by the investigator). If the investigator considers that the subject's conditions on the day of the visit would temporarily not allow inoculation, vaccination can be rescheduled within 7 days:

- Physical examination;
- Vital signs;
- Urine/blood pregnancy test;
- Review of inclusion/exclusion criteria;
- Vaccination;
- Site observation for 30min following inoculation;
- Recording concomitant medications;
- Collecting SAEs/MAAEs/AESIs;

➢ **Second vaccination visit:**

To be performed 14 (±14) days after the first dose

- Physical examination;
- Vital signs;
- Urine/blood pregnancy test;
- Reviewing of inclusion/exclusion criteria;
- Vaccination;

- Site observation for 30min following inoculation;
- Recording concomitant medications;
- Collecting SAEs/MAAEs/AESIs;

Note: COVID-19 case monitoring will be ceased after the unblinding in the site, and SAE/MAAE/AESI will be collected via telephone follow-up or other communication methods at least once every 4 weeks. Subjects can also actively report SAE/MAAE/AESI through the completion of the study. The blinded subjects in other study sites will continue to be followed up for vaccine efficacy and safety as originally specified.

Subject Data Analysis after Cross-over

Data collected after cross-over will only be used for safety analysis, as a supplementary analysis in this study.

Appendix 4 WHO Criteria for Grading of COVID-19 Cases [1]

Status:	Definition	Score
Uninfected	Not infected, nucleic acid negative	0
Mild	Asymptomatic, nucleic acid positive;	1
	Mild symptoms, not requiring treatment	2
	Mild symptoms, requiring treatment	3
General cases requiring hospitalization	Hospitalization; no oxygen therapy required*	4
	Hospitalization; oxygen therapy through a mask or nasal cavity	5
Severe cases requiring hospitalization	Treatment by non-invasive ventilation or high flow oxygen inhalation	6
	Intubation and mechanical ventilation, $pO_2/FiO_2 \geq 150$, or $SpO_2/FiO_2 \geq 200$	7
	Mechanical ventilation $pO_2/FiO_2 < 150$ ($SpO_2/FiO_2 < 200$) or use of vasopressors	8
	Mechanical ventilation $pO_2/FiO_2 < 150$ and use of vasopressors, dialysis, or extracorporeal membrane oxygenation (ECMO))	9
Death	Death	10

FiO_2=fraction of inspired oxygen, pO_2=partial pressure of oxygen. SpO_2=oxygen saturation.* If the hospitalization is due to quarantine alone, the status is to be recorded as a non-bedridden patient.

[1] Marshall JC, Murthy S, Diaz J, et al. A minimal common outcome measure set for COVID-19 clinical research. Lancet Infet Dis 2020;20:e192-97.

Appendix 5 Clinical Classification of COVID-19 issued by National Health Commission of the People's Republic of China

(I) Mild

The clinical symptoms are mild, and there are no radiological findings of pneumonia.

(II) General

Showing fever and respiratory symptoms with radiological findings of pneumonia.

(III) Severe

Adult cases meeting any of the following criteria:

(1) Respiratory distress, RR≥30/min;

(2) Oxygen saturation ≤93% at resting;

(3) Arterial partial pressure of oxygen (PaO2)/fraction of inspired oxygen (FiO_2)≤ 300mmHg (l mmHg=0.133kPa); in high-altitude areas (at an altitude of over 1,000 meters above sea level), PaO_2/FiO_2 shall be corrected by the following formula: PaO_2/FiO_2×[760/atmospheric pressure (mmHg)].

(4) Cases with progressive severe clinical symptoms and chest imaging that shows obvious lesion progression within 24-48 hours >50%.

(IV) Critical

Cases meeting any of the following criteria:

(1) Respiratory failure requiring mechanical ventilation;

(2) Shock;

(3) Organ failures requiring ICU care.

Appendix 6 Country-specific requirements

1、Requirement in South Africa

Concerning the "STUDY POPULATION"

In line with the local regulatory requirements in South Africa, this study (COVID-19-PRO-003) will only enroll those subjects who have received at least one dose of other COVID-19 vaccines (marketed or investigational) with an interval of ≥ 6 months between the last dose and the date when the subjects sign the informed consent.

2、Requirement in Vietnam

Concerning the "STUDY POPULATION"

Based on the current actual vaccination situation for the COVID-19 vaccine in Vietnam (most subjects are within 3 months after the last dose) and the Vietnamese health ministry has suggested that booster shots can be eligible after three months from second COVID-19 vaccine dose. This study (COVID-19-PRO-003) will enroll those subjects who have received at least one dose of other COVID-19 vaccines (marketed or investigational) with an interval of ⩾ 3 months between the last dose and the date when the subjects sign the informed consent for this study (reference to inclusion criteria no #3). This is only applicable to sites in Vietnam.

This addendum is to be performed in addition to all procedures required by protocol COVID-19-PRO-003 or any subsequent amendments.

List of Changes

Revised in Section:

Revised Section	Original Text	Revised Text	Reason for Revision
Section 4.2 Inclusion criteria (no #3)	Subjects who have not received any COVID-19 vaccine (marketed or investigational), those who have received at least one dose of other COVID-19 vaccines (marketed or investigational) with an interval of ⩾ 6 months between the last dose and the date when the subjects sign the informed consent for this study.	Subjects who have received at least one dose of other COVID-19 vaccines (marketed or investigational) with an interval of ⩾ 3 months between the last dose and the date when the subjects sign the informed consent for this study.	To comply with the current actual vaccination situation for the COVID-19 vaccine in Vietnam.

Rationale for Addendum

This study COVID-19-PRO-003 is a Global, Multi-center, Randomized, Double-blind, Placebo-controlled study. The objective of this addendum is to facilitate the enrollment of subjects in Vietnam.
